# Finite element modeling of sarcoplasmic reticular intraluminal Ca^2+^ diffusional fluxes during amphibian striated muscle excitation–contraction coupling

**DOI:** 10.3389/fphys.2026.1774977

**Published:** 2026-04-28

**Authors:** Katherine J.-X. Lin, Marco D. Rodriguez, Joshua A. Morris, Oliver J. Bardsley, Hugh R. Matthews, Christopher L.-H. Huang

**Affiliations:** 1 Physiological Laboratory, University of Cambridge, Cambridge, United Kingdom; 2 Cambridge University School of Clinical Medicine, Cambridge, United Kingdom; 3 Department of Veterinary Medicine, University of Cambridge, Cambridge, United Kingdom; 4 Department of Biochemistry, University of Cambridge, Cambridge, United Kingdom

**Keywords:** Ca2+ diffusion, calsequestrin, excitation-contraction coupling, ryanodine receptor, sarcoplasmic reticular Ca2+-ATPase, sarcoplasmic reticulum, triad junctions

## Abstract

**Introduction:**

Excitation–contraction coupling involves sarcoplasmic reticular (SR) ryanodine receptor (RyR)-mediated Ca^2+^ release. The latter is driven by cisternal SR (CSR) [Ca^2+^], which, in turn, is determined by SR intraluminal Ca^2+^ diffusion patterns over timescales and spatial features precluding direct experimental study.

**Materials and methods:**

We modeled these diffusive fluxes and consequent total, [Ca^2+^]_total_, and free Ca^2+^ concentration, [Ca^2+^]_free_, patterns. This utilized established amphibian skeletal muscle SR anatomy, initial resting [Ca^2+^]_total_, [Ca^2+^]_free_, calsequestrin concentrations [Casq] and dissociation constants, and their related Ca^2+^ diffusion coefficients. Step increases in CSR membrane Ca^2+^ permeabilities were modeled to give initial CSR Ca^2+^ release rates compatible with previous reports. The latter were either held constant or permitted to decay with the consequent CSR [Ca^2+^]_free_ depletion.

**Results:**

SR anatomy was quantifiable as a CSR giving rise to multiple longitudinal SR (LSR), with a radius of 15 nm and length of 1,800 nm, extensions. The CSR Ca^2+^ release produced time-dependent spatial SR Ca^2+^ color maps. These showed time-evolving, axial SR [Ca^2+^]_free_ and [Ca^2+^]_total_ gradients over 0–20 ms. Calsequestrin increased the absolute [Ca^2+^]_free_ and [Ca^2+^]_total_ values, slowed their temporal decays, and reduced their axial gradients. There were no significant radial LSR [Ca^2+^] gradients. These findings implicated both calsequestrin and LSR geometry in determining the Ca^2+^ diffusion patterns. Calsequestrin also slowed the decays of the resulting T–SR membrane Ca^2+^ fluxes with time. Background SERCA activity levels contrastingly contributed negligibly to either [Ca^2+^] patterns or these diffusional fluxes. Patterns of declining [Ca^2+^]_free_ and [Ca^2+^]_total_ and SR Ca^2+^ effluxes and effects or otherwise of calsequestrin and/or SERCA activity also occurred over timescales longer than <2 s. However, both longitudinal and radial [Ca^2+^] gradients were now undetectable, suggesting that diffusional equilibrium had now been reached along the SR axis.

**Conclusion:**

Available electron microscopic evidence permitted a quantitative description of SR geometry. This underpinned the simplified modeling of SR intraluminal Ca^2+^ diffusion, Ca^2+^–calsequestrin buffering and background SERCA activity following the CSR Ca^2+^ release initiating excitation–contraction coupling. This predicted one-dimensional SR axial, but not radial, diffusional gradients over milliseconds, approximating equilibration over second timescales. These and the consequent SR Ca^2+^ effluxes were significantly sustained by calsequestrin buffering, but not background SERCA activity.

## 1 Introduction

Striated muscle excitation–contraction coupling involves the release of sarcoplasmic reticular (SR) store Ca^2+^ into the remaining cell cytosol. This is triggered through allosteric or Ca^2+^-induced interactions (Huang, 1996) between voltage-sensing transverse (T) tubular membrane dihydropyridine receptors (DHPRs) and SR cisternal membrane ryanodine receptor (RyR) Ca^2+^ release channels [(Chawla et al., 2002; Huang 1998a, b); (see review in Huang et al. (2011))]. This coupling occurs at discrete T–SR junctions between anatomically close T-tubular and SR cisternal membranes (see review in Martin et al. (2003)). The Ca^2+^ release itself is likely driven by free transmembrane Ca^2+^ concentration gradients between the cisternal SR (CSR) lumen and the remaining cytosol. However, key to the latter process are the baseline Ca^2+^ diffusive properties within the SR lumen in resting muscle, itself characterizable following step changes in CSR membrane Ca^2+^ permeability. The latter would determine the resulting spatial and temporal SR intraluminal Ca^2+^ diffusion and concentration patterns.

The sarcoplasmic reticular (SR) luminal Ca^2+^ store in skeletal muscle contains an overall background [Ca^2+^]_total_ of ~3.6 mM (Volpe and Simon, 1991). This is five orders of magnitude greater than the resting ~50 nM cytosolic [Ca^2+^]. However, CSR Ca^2+^ release then potentially produces rapid alterations in its own driving CSR free Ca^2+^ concentration, [Ca^2+^]_free_ (Baylor and Hollingworth, 1998, 2007). This, in turn, produces Ca^2+^ diffusional changes in the remaining longitudinal SR (LSR) Ca^2+^ store. The latter comprises a complex tubular network permitting the development of time-dependent and spatially dependent Ca^2+^ gradients (Birks and Davey, 1969; Mobley and Eisenberg, 1975; Peachey, 1965). Furthermore, multiple SR proteins, particularly calsequestrin (Casq), variously buffer, store, and release Ca^2+^ (Reviews: (Beard et al., 2004; Dulhunty, 2006)). Finally, the SR membrane Ca^2+^–ATPase (SERCA) actively pumps cytosolic Ca^2+^ into and thereby replenishes SR lumen calcium levels (MacLennan and Wong, 1971). All three factors interact to contribute to the SR Ca^2+^ intraluminal gradients.

Direct experimental fluorescence imaging of intra-SR calcium loaded membrane-permeable acetoxymethyl ester derivatives of synthetic fluorimetric dyes or targeted specific protein-based Ca^2+^ sensors to the SR lumen (Robin and Allard, 2013; Rudolf et al., 2006; Sztretye et al., 2011; Ziman et al., 2010). These probes were of low affinity to avoid saturation by the high resting free SR Ca^2+^ concentrations. However, in relationship to detailed intra-SR diffusional Ca^2+^ concentration gradients early in the excitation–contraction coupling, they are currently limited in their response times and spatial localization and potentially themselves perturb the processes under study. Even rapid fluorescent Ca^2+^ indicators, exemplified by jGCaMP8, only offer ≥~2 ms temporal resolution (e.g., Zhang et al., 2023). Their visualization techniques that include two-photon confocal microscopy have ~1-µm spatial resolutions. Finally, many probes are themselves derivatives of actual *in vivo* Ca^2+^ buffers such as calsequestrin itself that would therefore perturb the normal Ca^2+^ diffusion patterns. These techniques nevertheless invaluably visualized overall as opposed to mapping localized SR Ca^2+^ over relatively *prolonged* intervals (Robin and Allard, 2013; Sobie and Lederer, 2012; Somlyo et al., 1981; Ziman et al., 2010) as following repetitive as opposed to single stimulations (Rudolf et al., 2006) or during slow wave phenomena (Chawla et al., 2001; Cully et al., 2014). Additionally, recent diffusional–compartmental analyses modeled calcium diffusional transients *between* cytosolic, mitochondrial, and SR subcompartments located in 2D space and the role in these of membrane transporters and Ca^2+^ buffering in mammalian skeletal myocytes (Marcucci et al., 2018). Related studies were made in mammalian cardiac myocytes (Bers and Shannon, 2013; Hatano et al., 2011; Picht et al., 2011).

Modeling methods could additionally provide useful limiting indications particularly of *intra*-SR diffusion patterns *early* following the initiation of excitation–contraction coupling. The present studies analyze Ca^2+^ diffusion patterns, quantified as free ([Ca^2+^]_free_) and total ([Ca^2+^]_total_), including calsequestrin (Casq)-bound, [Ca^2+^], *within* the SR lumen itself. This diffusional modeling of the resulting spatial and temporal SR [Ca^2+^] changes required a quantitative description of SR geometry. The latter could be reconstructed from previous detailed and quantitative morphological data specifically available for amphibian skeletal, as opposed to mammalian skeletal or cardiac, muscle (Birks and Davey, 1969; Mobley and Eisenberg, 1975; Peachey, 1965). Each half SR element was dimensioned, using established electron microscopy results, as a cylinder with cisternal (CSR) and longitudinal ends (LSR). These permitted a generation and time evolution of longitudinal and radial [Ca^2+^] gradients following the initiation of CSR Ca^2+^ release. The computations were initiated at the onset of RyR-Ca^2+^ release channel opening. The latter was modeled to give initial rates of cytosolic free [Ca^2+^] increase previously associated with excitation–contraction coupling (Bardsley et al., 2021; Kovacs et al., 1983). The computational studies compared the effects of constant rates of that Ca^2+^ release with those of rates decaying with the consequent CSR Ca^2+^ depletion. The differential equation solutions extended both over subsequent 0–20-ms and more prolonged 0–2-s intervals corresponding, respectively, to single or sustained SR Ca^2+^ release activity. We also examined the consequences of additionally including immobile previously reported calsequestrin buffer concentrations [Casq] on the longitudinal and radial SR Ca^2+^ diffusion rates. These would exert effects on the effective Ca^2+^ diffusion coefficients and on the overall amounts of Ca^2+^ eventually diffused through its effect on Ca^2+^ storage. Finally, inclusion of a SERCA-mediated Ca^2+^ influx into the SR Ca^2+^ store might alter longitudinal and radial [Ca^2+^] changes with time.

These explorations complemented a previous analysis similarly performed for cytosolic as opposed to SR [Ca^2+^] patterns early in amphibian skeletal muscle excitation–contraction coupling. These had modeled the binding and diffusion of Ca^2+^ after its release at the Z line in response to an action potential within a half sarcomere (Baylor and Hollingworth, 1998; Baylor et al., 1983; Cannell and Allen, 1984). More recent studies have also modeled patterns of Ca^2+^ diffusion and the effects of buffering following Ca^2+^ release into the T–SR junction itself (Bardsley et al., 2021; Rodríguez et al., 2024).

## 2 Theory

### 2.1 Overview

**Figure 1:**
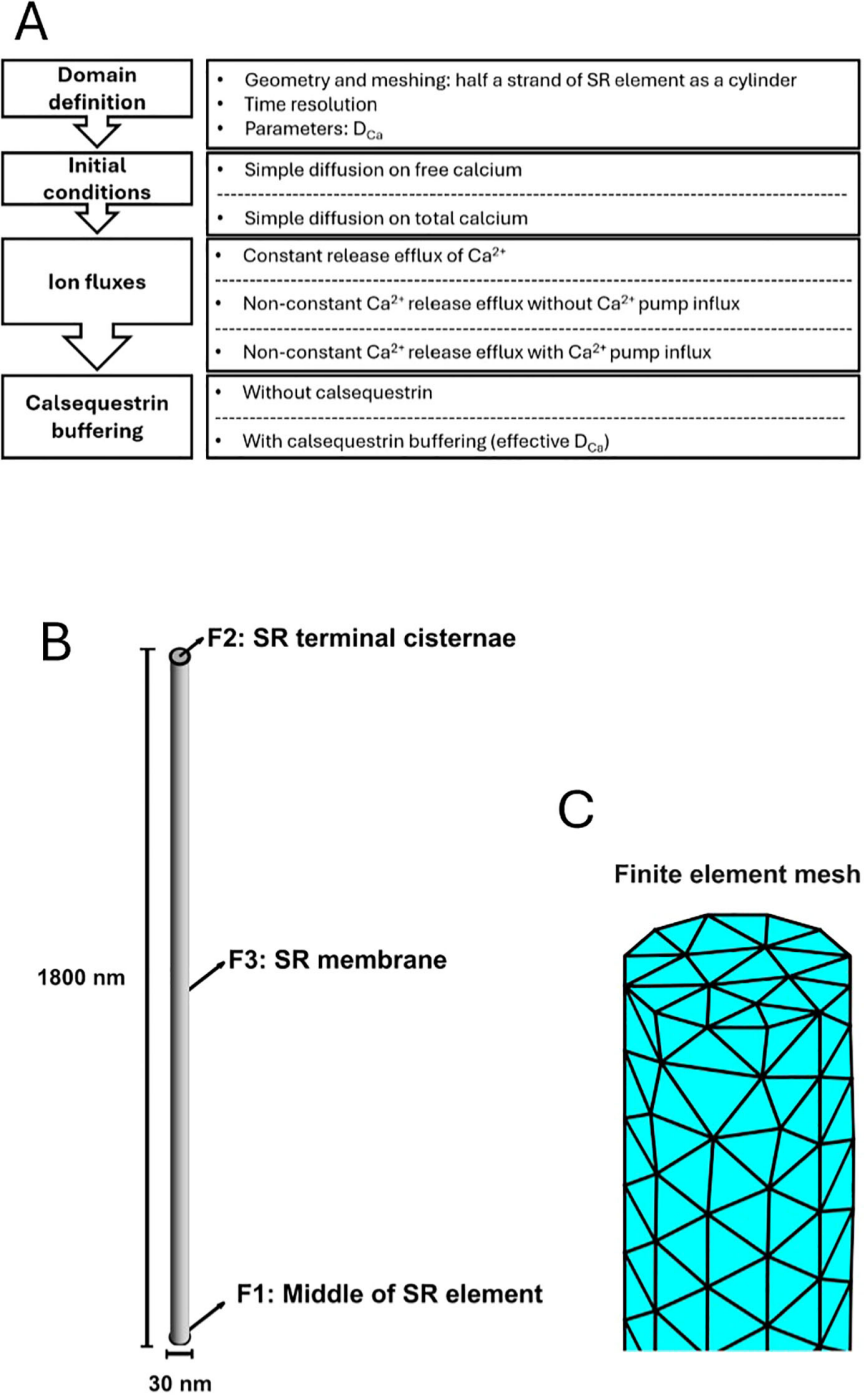
Basic modeling strategy and geometry of the sarcoplasmic reticular (SR) element forming the diffusion volume. **(A)** Summary of computational strategy. **(B)** Representation of the geometry of each modeled SR element as a cylinder joining the midpoint of the longitudinal SR (LSR) to the cisternal region (CSR) from which RyR-mediated Ca^2+^ release into the T–SR junction takes place. This structure is, in turn, **(C)** meshed into finite elements. In **(B)**, face F1 represents the middle of the LSR element or the end of the half LSR element and is the closed end. F2 is the terminal cisterna forming the opposite CSR end. F3 is the lateral membrane of the SR element.

The computations (**Figure 1A**) first modeled SR Ca^2+^ diffusion early in excitation–contraction coupling. They utilized an SR anatomy comprising a CSR associated with multiple component longitudinal SR strands. The latter were each assigned lengths, *l*, and diameters, *a*_ΔSR_, quantified (**Figure 1B**; **Table 1**) in previous electron microscopy studies on amphibian skeletal muscle (Peachey, 1965). The finite element analysis (**Figure 1C**) utilized initial conditions with established resting values for free Ca^2+^, [Ca^2+^]_free_, and total calsequestrin concentrations [Casq]_total_ (Volpe and Simon, 1991). Through known calsequestrin–Ca^2+^ binding constants *K_d_*, this further yielded the initial as well as the subsequent total Ca^2+^, [Ca^2+^]_total_, and bound and free calsequestrin concentrations [Casq]_free_. Of the boundary conditions, the RyR-mediated cisternal membrane Ca^2+^ efflux densities from the cisternal, CSR, of each strand, $J_{\text{efflux}}^{\left(t\right)}$ were defined from established initial (*t* = 0) cisternal efflux densities $J_{\text{TSR}}^{\left(\text{o}\right)}$ associated with maximum voltage-dependent rates of increase in cytosolic Ca^2+^ (Bardsley et al., 2021; Kovacs et al., 1983; Rodríguez et al., 2024), morphometric ratios between SR and the whole fiber volumes *V*_SR_*, and the T-tubular membrane area, *A*_TSR_, participating in TSR junctions within the half sarcomere in a muscle of diameter *a.* Background SERCA-mediated influx densities $J_{\Delta \text{SR influx}}^{\left(t\right)}$ were similarly derived from previously reported TSR Ca^2+^ release rates corresponding to resting background [Ca^2+^]_i_ levels (Bardsley et al., 2021; Blatter and Blinks, 1991; Harkins et al., 1993; Konishi and Watanabe, 1995; Kurebayashi et al., 1993). The subsequent diffusive modeling then reconstructed the spatial and temporal patterns of both [Ca^2+^]_total_ and [Ca^2+^]_free_ and their cisternal transmembrane Ca^2+^ fluxes into the TSR junctions. This assumed the established free Ca^2+^ diffusion coefficient, *D*_Ca_^2+^, and calsequestrin affinity for Ca^2+^*, K*_d_. Solutions were obtained under conditions severally including and excluding calsequestrin and SERCA activity, with the proviso that in the absence of calsequestrin, [Ca^2+^]_total_ ≈ [Ca^2+^]_free_. They were obtained through early (0–20 ms) and more prolonged (0–2 s) intervals following the onset of Ca^2+^ release to permit comparisons to be made.

**Table 1:** Parameters used for modeling the amphibian skeletal muscle fibers and sarcoplasmic reticulum.

Variables	Symbol	Value (physiological units)	Dimension (physiological units)	Value (SI units)	Dimension (SI units)	Source
(i) Sarcoplasmic reticulum geometry						
Length of sarcomere	*l*	3.6	µm	3.6 × 10^-6^	m	(Gordon et al., 1966)
Diameter of fiber	*a*	100	µm	100 × 10^-6^	m	(Adrian and Peachey, 1973)
Surface membrane capacitance	*C*_C_	1.0	μF cm^-2^	1.00 × 10^–2^	F m^-2^	(Adrian and Peachey, 1973)
Transverse (T-) tubular membrane capacitance	*C*_T_	5.0	μF cm^-2^	5.00 × 10^–2^	F m^-2^	(Falk and Fatt, 1964)
Sarcomere tubular membrane area	*A*_T_ * = (C*_T_ */C*_S_ *)πal*	5,733	μm^2^	5.733 × 10^–9^	m^2^	(Bardsley et al., 2021)
Proportion of T-tubular membrane area apposed to triad junctions	*ξ*	0.3		0.3		(Franzini-Armstrong, 1970)
Tubular membrane area abutted by T–SR junction	*A*_TSR_ *= ξA_T_*	1,720	μm^2^	1.720 × 10^–9^	m^2^	(Bardsley et al., 2021)
Ratio between the SR volume and the whole fiber volume	*V*_SR_ *	12%		12%		(Mobley and Eisenberg, 1975; Peachey, 1965)
Diameter of SR element	*a_Δ_*_SR_	0.03	µm	3.00 × 10^-8^	m	(Peachey, 1965; Somlyo et al., 1981)
(ii) Diffusion coefficients and Ca^2+^ concentrations						
Ca^2+^ diffusion coefficients	*D*_Ca_^2+^	(free) 3 × 10^-6^	cm^2^ s^-1^	(free) 3 × 10^-10^	m^2^ s^-1^	(Baylor and Hollingworth, 2007; Bers and Shannon, 2013; Picht et al., 2011; Sobie and Lederer, 2012)
SR concentration of total calsequestrin	[Casq]_total_	6.1	mM	6.1	mol m^-3^	(Volpe and Simon, 1991)
Initial (*t* = 0) free SR Ca^2+^ concentration	${\left[{\text{Ca}}^{2+}\right]}_{\text{free}}^{\left(\text{x},0\right)}$	3.6	mM	3.6	mol m^-3^	(Volpe and Simon, 1991)
Initial (*t* = 0) total SR Ca^2+^ concentration	${\left[{\text{Ca}}^{2+}\right]}_{\text{total}}^{\left(\text{x},0\right)}$	8.2723	mM	8.2723	mol m^-3^	(Volpe and Simon, 1991)
Calsequestrin affinity for Ca^2+^	*K*_d_	1,100	µM	1.1	mol m^-3^	(Volpe and Simon, 1991)
(iii) Ca^2+^ flux densities						
Initial flux density of Ca^2+^ release efflux out of a cisternal sarcoplasmic reticular (CSR) element	$J_{\text{efflux}}^{\left(t\right)}$	-2.7375 × 10^-24^	mol nm^-2^s^-1^	-2.7375 × 10^-6^	mol m^-2^s^-1^	(Bardsley et al., 2021)
Flux density of Ca^2+^–ATPase (SERCA)-mediated Ca^2+^ influx into a SR element	$J_{\Delta \text{SR influx}}^{\left(t\right)}$	3.6880 × 10^-28^	mol nm^-2^s^-1^	3.6880 × 10^-10^	mol/m^-2^s	(Bardsley et al., 2021)
(iv) Temporal and spatial computational resolutions						
Number of time points calculated for each run		1,000		1,000		
Maximal edge length for meshing	*H*_max_	10	nm	1 × 10^-8^	m	

### 2.2 MATLAB partial differential equation toolbox implementation

The partial differential diffusional equations (PDEs) exploring the diffusional ion fluxes and total Ca^2+^ concentrations, [Ca^2+^]_total_, were adapted to the format in Equation 1 solvable by the MATLAB PDE Toolbox:

(1) $$\lambda_{1}\frac{\partial^{2}c}{\partial t^{2}}+\lambda_{2}\frac{\partial c}{\partial t}-\nabla .(\lambda_{3}\nabla c)+\lambda_{4}c-\lambda_{5}=0$$

The equation solution is the concentration term *c*. The latter is a function of radial and longitudinal position within the SR element, and time *t* following initiation of the Ca^2+^ effluxes and influxes. The coefficients $\lambda_{1}$- $\lambda_{5}\;$ are functions of location, and can also be functions of *c* or its gradients, at time *t*. The product rule gives Equation 2:

(2) $$\nabla .(\lambda_{3}\nabla c)=\nabla \lambda_{3}.\nabla c+\lambda_{3}\nabla^{2}c$$

Given that $\lambda_{3},\text{ whic}$h represents the diffusion coefficient, is uniform, $\nabla \lambda_{3}=0$. This gives Equation 3:

(3) $$\nabla .(\lambda_{3}\nabla c)=\lambda_{3}\nabla^{2}c$$

This gives:

(4) $$\lambda_{1}\frac{\partial^{2}c}{\partial t^{2}}+\lambda_{2}\frac{\partial c}{\partial t}-\lambda_{3}\nabla^{2}c+\lambda_{4}c-\lambda_{5}=0$$

The adopted diffusive flux equation for Fick’s second law in three dimensions is:

(5) $$\frac{\partial c}{\partial t}=D\nabla^{2}c$$

with $\frac{\partial c}{\partial t}$ the rate of concentration change (in mol.m^-3^ s^-1^), *D* the diffusion coefficient (in m^2^ s^-1^) and *c* the concentration (in mol m^-3^). Comparing terms in Equations 4 and 5 then yields Equation 6:

(6) $$\begin{array}{c} \lambda_{1}=0\\ \lambda_{2}=1\\ \lambda_{3}=D\\ \lambda_{4}=0\\ \lambda_{5}=0 \end{array}$$

### 2.3 Geometrical definition of the SR element

Previous electron microscopic characterizations (Peachey, 1965) described a amphibian skeletal muscle longitudinal SR comprising a network of parallel strands surrounding the muscle microfibrils (**Figure 1B**). Each strand can be formalized as a cylinder, radius, $\frac{a_{\text{ΔSR}}}{2}=15\;\text{nm},\;$ symmetrical about the midpoint, designated the LSR, of its total length, *l*, extending through its containing sarcomere. This permitted a numerical MATLAB PDE toolbox modeling of a geometric element of length half that of the sarcomere, $\frac{l}{2}=1.8\;\text{μm}$ extending between the CSR and LSR ( (Gordon et al., 1966); **Table 1**). This could be meshed into elements for finite element analysis with a resolution of $H_{\text{max}}=10\;\text{nm}$. (magnified portion shown in **Figure 1C**). The formalized element has surfaces F1 representing the end of the half SR element, LSR, F2 the SR terminal cisternal CSR membrane releasing Ca^2+^ into the tubular-sarcoplasmic reticular (TSR) junction and F3 the lateral SR membrane (**Figure 1B**).

### 2.4 Initial conditions

Each computational run assumed each SR element initially (time, *t* = 0) entirely filled with a uniform total Ca^2+^ concentration ${\left[{\text{Ca}}^{2+}\right]}_{\text{total}}$. If at the CSR end, longitudinal position *x* = 0, ${\left[{\text{Ca}}^{2+}\right]}_{\text{total}}^{\left(x,t\right)}={\left[{\text{Ca}}^{2+}\right]}_{\text{total}}^{\left(0,0\right)}$, then, along the entire length 0 < *x* <*l*/2 extending to the center of the longitudinal, LSR, Equation 7 gives:

(7) $${\left[{\text{Ca}}^{2+}\right]}_{\text{total}}^{\left(x,t\right)}={\left[{\text{Ca}}^{2+}\right]}_{\text{total}}^{\left(x,0\right)}=\;{\left[{\text{Ca}}^{2+}\right]}_{\text{total}}^{\left(0,0\right)}$$

On RyR opening at *t* = 0, Ca^2+^ diffuses across the CSR membrane into the T–SR space and thence to the remaining cytosolic [Ca^2+^] space with total Ca^2+^ concentration, ${\left[{\text{Ca}}^{2+}\right]}_{\text{cyt}}^{\left(0,t\right)},$ at all times *t*, much smaller than the total SR cisternal Ca^2+^ concentration. Thus:

(8) $${\left[{\text{Ca}}^{2+}\right]}_{\text{total}}^{(x,t)\;}\gg \{{\left[{\text{Ca}}^{2+}\right]}_{\text{cyt}}^{\left(0,t\right)}\approx 0\}$$

The ${\left[{\text{Ca}}^{2+}\right]}_{\text{total}}^{\left(0,0\right)}$ was estimated from previously experimentally determined resting concentrations of free SR Ca^2+^, ${\left[{\text{Ca}}^{2+}\right]}_{\text{free}}=3.6\text{ mM}=3.6{\text{ mol m}}^{-3}$, and of total calsequestrin, [Casq]_total_ = 6.1 mM = 6.1 mol m^-3^, along with its dissociation constant for Ca^2+^, $K_{\text{d}}=1.1\text{ mM}=1.1{\text{ mol m}}^{-3}.$ The derivation in Equations 9–17 assumes a 1:1 Ca^2+^-Casq binding (Volpe and Simon, 1991). Since [Ca^2+^]_total_ and ${\left[\text{Casq}\right]}_{\text{total}}\;$ are the sums respectively of the [Ca^2+^]_free_ and free [Casq]_free_, and the bound calsequestrin, concentration [Casq-Ca^2+^]:

(9) $${\left[{\text{Ca}}^{2+}\right]}_{\text{total}}={\left[{\text{Ca}}^{2+}\right]}_{\text{free}}+[\text{Casq}-{\text{Ca}}^{2+}]$$

This gives:

(10) $$[\text{Casq}-{\text{Ca}}^{2+}]={\left[{\text{Ca}}^{2+}\right]}_{\text{total}}-{\left[{\text{Ca}}^{2+}\right]}_{\text{free}}$$

And:

(11) $$\begin{array}{l} {\left[\text{Casq}\right]}_{\text{total}}={\left[\text{Casq}\right]}_{\text{free}}+[\text{Casq}-{\text{Ca}}^{2+}]\\ ={\left[\text{Casq}\right]}_{\text{free}}+{\left[{\text{Ca}}^{2+}\right]}_{\text{total}}-{\left[{\text{Ca}}^{2+}\right]}_{\text{free}} \end{array}$$

Together these give:

(12) $${\left[\text{Casq}\right]}_{\text{free}}={\left[\text{Casq}\right]}_{\text{total}}-{\left[{\text{Ca}}^{2+}\right]}_{\text{total}}+{\left[{\text{Ca}}^{2+}\right]}_{\text{free}}$$

Substituting the above into the expression for *K*_d,_

(13) $$K_{d}=\frac{{\left[{\text{Ca}}^{2+}\right]}_{\text{free}}{\left[\text{Casq}\right]}_{\text{free}}}{\left[\text{Casq}-{\text{Ca}}^{2+}\right]}$$

Gives:

(14) $$K_{\text{d}}=\frac{{\left[{\text{Ca}}^{2+}\right]}_{\text{free}}({\left[\text{Casq}\right]}_{\text{total}}-{\left[{\text{Ca}}^{2+}\right]}_{\text{total}}+{\left[{\text{Ca}}^{2+}\right]}_{\text{free}})}{{\left[{\text{Ca}}^{2+}\right]}_{\text{total}}-{\left[{\text{Ca}}^{2+}\right]}_{\text{free}}}$$

Rearranging the [Ca^2+^]_total_ on one side:

(15) $$\begin{array}{l} K_{\text{d}}{\left[{\text{Ca}}^{2+}\right]}_{\text{total}}={\left[{\text{Ca}}^{2+}\right]}_{\text{free}}({\left[\text{Casq}\right]}_{\text{total}}-{\left[{\text{Ca}}^{2+}\right]}_{\text{total}}+{\left[{\text{Ca}}^{2+}\right]}_{\text{free}})\\ +K_{\text{d}}{\left[{\text{Ca}}^{2+}\right]}_{\text{free}} \end{array}$$

Factorizing:

(16) $$\begin{array}{l} (K_{\text{d}}+{\left[{\text{Ca}}^{2+}\right]}_{\text{free}}){\left[{\text{Ca}}^{2+}\right]}_{\text{total}}\\ ={\left[{\text{Ca}}^{2+}\right]}_{\text{free}}({\left[\text{Casq}\right]}_{\text{total}}+{\left[{\text{Ca}}^{2+}\right]}_{\text{free}})+K_{d}{\left[{\text{Ca}}^{2+}\right]}_{\text{free}} \end{array}$$

We obtain the expression:

(17) $$\begin{array}{c} {\left[{\text{Ca}}^{2+}\right]}_{\text{total}}=\frac{{\left[{\text{Ca}}^{2+}\right]}_{\text{free}}({\left[\text{Casq}\right]}_{\text{total}}+{\left[{\text{Ca}}^{2+}\right]}_{\text{free}}+K_{\text{d}})}{K_{\text{d}}+{\left[{\text{Ca}}^{2+}\right]}_{\text{free}}}\\ =8.2723\;\text{mM}=\;8.2723\;\times 10^{-3}\;\text{M},\text{ from Table }1 \end{array}$$

### 2.5 Flux boundary conditions

The initial conditions for the modeling process assumed uniform resting ion concentrations equal to their corresponding bulk cytosolic concentrations (**Table 1**). Neumann boundary conditions (BCs) described free Ca^2+^ fluxes in and out of the geometry at the edges of the domain, entering and leaving the junction. These set the derivative at each boundary to be equal to a constant. In MATLAB, Neumann BCs are defined by Equation 18:

(18) $$\vec{n}\;\cdot (h\nabla \text{c})+pc=g$$

with $\vec{n}$ the outward unit normal, *h* a constant coefficient, *c* the solution, *p* the transfer coefficient, and *g* the flux density. The sections that follow determined the value *g* for the free Ca^2+^ release efflux and pump influx. Of the modeled SR membrane components, the F1 surface represented the Ca^2+^ impermeant LSR end of the half SR element. The F2 surface represented the CSR generating a SR efflux of free Ca^2+^ into the TSR space. The F3 surface represented the lateral SR membrane forming the site for SERCA-mediated Ca^2+^ entry.

### 2.6 The RyR-mediated SR Ca^2+^ release efflux at the F2 surface

The RyR-mediated SR Ca^2+^ release efflux at the F2 surface was derived as a release efflux density $J_{\text{efflux}}^{\left(t\right)}$. This combined the SR elements within each half sarcomere into an equivalent cylinder. Equations 19 and 20 derive its effective diameter $a_{SR}$. We assume a muscle fibre of diameter *a* = 100 μm (Adrian and Peachey, 1973) and previously reported ratios between the SR and the whole fibre volumes *V*_SR_^*^=12% (Birks and Davey, 1969; Mobley and Eisenberg, 1975; Peachey, 1965):

(19) $$V_{\text{SR}}^{*}=\frac{\pi a_{\text{SR}}^{2}/4}{\pi a^{2}/4}=\frac{a_{\text{SR}}^{2}}{a^{2}}$$

Thus, $a_{SR}$ is given by:

(20) $$a_{\text{SR}}=a\sqrt{V_{\text{SR}}^{*}}$$

The total free Ca^2+^ flux out of this lumped SR was obtained from the initial free Ca^2+^ efflux through unit CSR membrane area from the whole sarcomere. This was set to a value, $J_{\text{TSR}}^{\left(o\right)}$ = -2.7375 × 10^–24^ mol nm^-2^ s^-1^ = -2.7375 × 10^–6^ mol m^-2^ s^-1^ adopted by the previous modeling of Ca^2+^ release into the TSR space (Bardsley et al., 2021). The latter matches previously reported maximum rates of increase in cytosolic Ca^2+^ concentration during a voltage-sensitive excitation–contraction coupling (Kovacs et al., 1983). Equation 21 accordingly gives the initial total efflux from all the available T-tubular and therefore the CSR junctional membrane area *A*_TSR_ = 1720 μm^2^ = 1.720 × 10^–9^ m^2^ participating in TSR junctions within the half sarcomere::

(21) $$\Phi_{\text{efflux}}^{\left(\text{o}\right)}=-\frac{J_{\text{TSR}}^{\left(o\right)}A_{\text{TSR}}}{2}$$

The minus sign denotes Ca^2+^ as leaving the SR space. Briefly, the previously derived (Bardsley et al., 2021) *A*_TSR_ term was determined from previously reported values (summarized in **Table 1**) of the proportion of T-tubular membrane area apposed to triad junctions *ξ* = 0.3 (Franzini-Armstrong, 1970), and the sarcomere tubular membrane area *A*_T_ = 5733 μm^2^ = 5.733 × 10^–9^ m^2^; hence, *A*_TSR_ = *ξA*_T._ (Bardsley et al., 2021). In turn, the *A*_T_ term, $A_{\text{T}}=(\frac{C_{T}}{C_{S}})\pi al$, utilized values of the ratio of tubular to surface membrane capacitances, $(\frac{C_{\text{T}}}{C_{\text{S}}})=5$, compatible with previous electrophysiological reports (Falk and Fatt, 1964). Equations 22 and 23 summarize the simpler of the two considered cases assuming a constant free Ca^2+^ efflux through the computational interval $J_{\text{efflux}}^{\left(t\right)}$ obtained by dividing the cross-sectional area of lumped SR:

(22) $$A_{\text{SR}}=\frac{\pi a_{\text{SR}}^{2}}{4}=\frac{\pi a^{2}V_{\text{SR}}^{*}}{4}$$

The free Ca^2+^ efflux across the unit cisternal SR cross-sectional area $J_{\text{efflux}}^{\left(t\right)}$ proceeding from the CSR lumen through the TSR junctional region to the remaining cytosol is then (Bardsley et al., 2021):

(23) $$\begin{array}{l} J_{\text{efflux}}^{\left(t\right)}=J_{\text{efflux}}^{\left(0\right)}=-\frac{2J_{\text{TSR}}^{\left(o\right)}A_{\text{TSR}}}{\pi a^{2}V_{\text{SR}}^{*}}=-2.7375\times 10^{-24}{\text{mol nm}}^{-2}{\text{s}}^{-1}\\ =-2.7375\times 10^{-6}{\text{mol m}}^{-2}{\text{s}}^{-1} \end{array}$$

Equations 24-32 derive the more physiologically realistic case, in which $J_{\text{efflux}}^{\left(t\right)}$ decreases with falls in the driving CSR free SR Ca^2+^ concentration ${\left[{\text{Ca}}^{2+}\right]}_{\text{free}}^{\left(0,\;t\right)}\;$ from its initial value ${\left[{\text{Ca}}^{2+}\right]}_{\text{free}\;}^{\left(0,0\right)}$ over time *t*. Fick's First Law then relates $J_{\text{efflux}}^{\left(t\right)}$ to effective diffusion coefficient, $\;D_{\text{TSR}}$, and concentration gradient, $\frac{\text{d}c^{\left(t\right)}}{\text{d}x},\;$terms:

(24) $$J_{\text{efflux}}^{\left(t\right)}=-\;D_{\text{TSR}}\frac{\text{d}c^{\left(t\right)}}{\text{d}x}$$

Approximating $\frac{dc^{\left(t\right)}}{dx}$ to the cisternal–cytosolic free Ca^2+^ concentration difference across equivalent diffusion distance Δx, 

(25) $$\frac{dc^{\left(t\right)}}{dx}\approx \;\frac{\text{Δ}c^{\left(t\right)}}{\text{Δ}x}=\frac{\left\{{\left[{\text{Ca}}^{2+}\right]}_{\text{free}}^{\left(0,t\right)}-{\left[{\text{Ca}}^{2+}\right]}_{\text{cyt}}^{\left(0,t\right)}\right\}}{\text{Δ}x}$$

Gives:

(26) $$J_{\text{efflux}}^{\left(t\right)}=-\;D_{\text{TSR}}\frac{{\left[{\text{Ca}}^{2+}\right]}_{\text{free}}^{\left(0,t\right)}-{\left[{\text{Ca}}^{2+}\right]}_{\text{cyt}}^{\left(0,t\right)}}{\Delta x}$$

In the case that *t* = 0,

(27) $$J_{\text{efflux}}^{\left(o\right)}=-\;D_{\text{TSR}}\frac{{\left[{\text{Ca}}^{2+}\right]}_{\text{free}}^{\left(0,0\right)}-{\left[{\text{Ca}}^{2+}\right]}_{\text{cyt}}^{\left(0,0\right)}}{\Delta x}$$

This gives an expression for $J_{efflux}^{\left(t\right)}\;$ in which values for all of the required terms are available:

(28) $$J_{\text{efflux}}^{\left(t\right)}=-\;J_{\text{efflux}}^{\left(o\right)}\frac{\left\{{\left[{\text{Ca}}^{2+}\right]}_{\text{free}}^{\left(0,t\right)}-{\left[{\text{Ca}}^{2+}\right]}_{\text{cyt}}^{\left(0,t\right)}\right\}}{\left\{{\left[{\text{Ca}}^{2+}\right]}_{\text{free}}^{\left(0,0\right)}-{\left[{\text{Ca}}^{2+}\right]}_{\text{cyt}}^{\left(0,t\right)}\right\}}$$

Thus, the values of $J_{\text{efflux}}^{\left(o\right)}$and ${\left[{\text{Ca}}^{2+}\right]}_{\text{free}}^{\left(0,0\right)}$ are defined by the initial conditions. Furthermore, the cytosolic free Ca^2+^ concentration,

(29) $${\left[{\text{Ca}}^{2+}\right]}_{cyt}^{\left(0,t\right)}\ll {\left[{\text{Ca}}^{2+}\right]}_{\text{free}}^{\left(x,t\right)},\text{ and so}$$

(30) $${\left[{\text{Ca}}^{2+}\right]}_{\text{cyt}}^{\left(0,t\right)}\approx 0$$

This gives:

(31) $$J_{\text{efflux}}^{\left(t\right)}=-\;J_{\text{efflux}}^{\left(o\right)}\frac{\left\{{\left[{\text{Ca}}^{2+}\right]}_{\text{free}}^{\left(0,t\right)}\right\}}{\left\{{\left[{\text{Ca}}^{2+}\right]}_{\text{free}}^{\left(0,0\right)}\right\}}$$

The release efflux density is then:

(32) $$J_{\text{efflux}}^{\left(t\right)}=-\frac{2J_{\text{TSR}}^{\left(o\right)}A_{\text{TSR}}}{\pi a^{2}V_{\text{SR}}^{*}}\;\frac{\left\{{\left[{\text{Ca}}^{2+}\right]}_{\text{free}}^{\left(0,t\right)}\right\}}{\left\{{\left[{\text{Ca}}^{2+}\right]}_{\text{free}}^{\left(0,0\right)}\right\}}$$

### 2.7 SERCA-mediated background Ca^2+^ influx across the F3 surface

Two cases were considered. First, there was the situation in which SERCA-mediated Ca^2+^ pump activity returning free Ca^2+^ to the SR was absent. Here $\Phi_{\text{influx}}^{\left(t\right)}=0$. Secondly, Equations 33–37 derive the case for the constant background Ca^2+^ refilling of SR strands that normally occurs in resting muscle fibres. This would alter SR Ca^2+^ over timescales more prolonged than those shown early in the activation of SR Ca^2+^ release. This equals its total background (*t* = ∞)  steady TSR Ca^2+^ release flux, for which (Bardsley et al., 2021):

(33) $$\Phi_{\text{influx}}^{\left(t\right)}=-\frac{J_{\text{TSR}}^{\left(\infty \right)}A_{\text{TSR}}}{2}$$

The $\Phi_{influx}^{\left(t\right)}$ is divided up into the large number, *n*, of elemental, ΔSR, cylinders making up the SR. Each has equal diameter *a*_ΔSR_ and end-cross-sectional area $\frac{\pi {\left(a_{\Delta \text{SR}}\right)}^{2}}{4}$. The number, *n*, of such elements making up the entire SR is then:

(34) $$n\approx \;{\left(\frac{a_{\text{SR}}}{a_{\Delta \text{SR}}}\right)}^{2}=\frac{a^{2}V_{\text{SR}}^{*}}{a_{\text{ΔSR}\;}^{2}}$$

The total SERCA-mediated SR Ca^2+^ influx into each element ΔSR is then the total SERCA-mediated SR Ca^2+^ influx into the entire SR divided by the number of elements:

(35) $$\Phi_{\Delta \text{SR influx}}^{\left(t\right)}=\;+\frac{\Phi_{\text{influx}}^{\left(\infty \right)}}{n}=\;+\frac{J_{\text{TSR}}^{\left(\infty \right)}A_{\text{TSR}}}{2n}$$

Note: The right-hand expression has the divisor 2*n* because we are considering half sarcomeres.

Then, dividing $\Phi_{\Delta \text{SR influx}}^{\left(t\right)}$ by the lateral membrane area of each SR strand gives the expression for $J_{\Delta \text{SR influx}}^{\left(t\right)}$:

(36) $$J_{\Delta \text{SR influx}}^{\left(t\right)}=+\frac{J_{\text{TSR}}^{\left(\infty \right)}A_{\text{TSR}}}{\left(\frac{2n\pi a_{\text{ΔSR}}l}{2}\right)}=\frac{J_{\text{TSR}}^{\left(\infty \right)}A_{\text{TSR}}}{n\pi a_{\text{ΔSR}}l}$$

Note: The right-hand expression now does not have the divisor 2 because we are considering the lateral surface area of half sarcomeres.

Substituting the value of *n* into the expression (Equation 34) and adopting a resting background Ca^2+^ flux density into the T–SR junctions, $J_{\text{TSR}}^{\left(\infty \right)}$ = 9.70 × 10^–26^ mol nm^-2^s^-1^ = 9.70 × 10^–8^ mol m^-2^ s^-1^ (Bardsley et al., 2021), corresponding to resting cytosolic free Ca^2+^concentration, [Ca^2+^]_cyt_ **=** 0.01 μmol dm^-3^ = 1.0 × 10^–5^ mol m^-3^ (Blatter and Blinks, 1991; Harkins et al., 1993; Konishi and Watanabe, 1995; Kurebayashi et al., 1993), then gives:

(37) $$\begin{array}{l} J_{\Delta \text{SR influx}}^{\left(t\right)}=\frac{J_{\text{TSR}}^{\left(\infty \right)}A_{\text{TSR}}}{(\frac{a^{2}V_{\text{SR}}^{*}}{a_{\text{ΔSR}\;}^{2}})\pi a_{\text{ΔSR}}l}=+\frac{J_{\text{TSR}}^{\left(\infty \right)}A_{\text{TSR}}a_{\Delta \text{SR}}}{a^{2}V_{\text{SR}}^{*}\pi l}\\ =3.6880\times 10^{-28}{\text{mol nm}}^{-2}{\text{s}}^{-1}\\ =3.688\times 10^{-10}{\text{mol m}}^{-2}{\text{s}}^{-1} \end{array}$$

### 2.8 Effects of calsequestrin buffering on the effective Ca^2+^ diffusion coefficient

Equations 38–51 model the consequent temporal and spatial [Ca^2+^]_total_ and [Ca^2+^]_free_ alterations were then modeled by applying Equation 5 relating partial derivatives of [Ca^2+^]_total_ and an effective diffusion coefficient.

(38) $$\frac{\partial {\left[{\text{Ca}}^{2+}\right]}_{\text{total}}}{\partial t}=D_{{\text{Ca}}^{2+}}^{*}\nabla^{2}{\left[{\text{Ca}}^{2+}\right]}_{\text{total}}$$

D*_Ca2+_ was derived from the weighted average diffusion coefficient obtained from the relative concentrations of free and bound Ca^2+^. This resulting non-constant coefficient, dependent on the value of [Ca^2+^]_total_ within each finite element, was used in the MATLAB program.

(39) $$D_{{\text{Ca}}^{2+}}^{*}=\frac{D_{{\text{Ca}}^{2+}}{\left[{\text{Ca}}^{2+}\right]}_{\text{free}}+D_{\text{Casq}}[\text{Casq}-{\text{Ca}}^{2+}]}{{\left[{\text{Ca}}^{2+}\right]}_{\text{total}}}$$

An anchored rather than mobile calsequestrin (Volpe and Simon, 1991) indicates a calsequestrin diffusion coefficient *D*_Casq_ = 0. Expressing [Ca^2+^]_free_ in terms of total [Ca^2+^]_total_ and bound calcium [Casq-Ca^2+^] gives (compare: (Rodríguez et al., 2024)):

(40) $$D_{{\text{Ca}}^{2+}}^{*}=\frac{D_{{\text{Ca}}^{2+}}({\left[{\text{Ca}}^{2+}\right]}_{\text{total}}-[\text{Casq}-{\text{Ca}}^{2+}])}{{\left[{\text{Ca}}^{2+}\right]}_{\text{total}}}$$

Using known values of [Ca^2+^]_total_ and [Casq]_total_ can solve for the value of [Casq-Ca^2+^] utilizing the expression for *K*_d_ given above:

(41) $$\begin{array}{l} K_{\text{d}}=\frac{{\left[{\text{Ca}}^{2+}\right]}_{\text{free}}{\left[\text{Casq}\right]}_{\text{free}}}{\left[\text{Casq}-{\text{Ca}}^{2+}\right]}\\ =\frac{\left({\left[{\text{Ca}}^{2+}\right]}_{\text{total}}-[\text{Casq}-{\text{Ca}}^{2+}])({\left[\text{Casq}\right]}_{\text{total}}-[\text{Casq}-{\text{Ca}}^{2+}]\right)}{\left[\text{Casq}-{\text{Ca}}^{2+}\right]} \end{array}$$

Rearranging the equation above gives:

(42) $$\begin{array}{l} K_{\text{d}}[\text{Casq}-{\text{Ca}}^{2+}]\\ =({\left[{\text{Ca}}^{2+}\right]}_{\text{total}}-[\text{Casq}-{\text{Ca}}^{2+}])({\left[\text{Casq}\right]}_{\text{total}}-[\text{Casq}-{\text{Ca}}^{2+}]) \end{array}$$

Hence:

(43) $$\begin{array}{l} K_{\text{d}}[\text{Casq}-{\text{Ca}}^{2+}]\\ =({\left[{\text{Ca}}^{2+}\right]}_{\text{total}}{\left[\text{Casq}\right]}_{\text{total}}-{\left[{\text{Ca}}^{2+}\right]}_{\text{total}}[\text{Casq}-{\text{Ca}}^{2+}]-[\text{Casq}\\ -{\text{Ca}}^{2+}]{\left[\text{Casq}\right]}_{\text{total}}+{\left[\text{Casq}-{\text{Ca}}^{2+}\right]}^{2}) \end{array}$$

This yields the quadratic equation:

(44) $$\begin{array}{l} ({\left[{\text{Ca}}^{2+}\right]}_{\text{total}}{\left[\text{Casq}\right]}_{\text{total}}-\{{\left[{\text{Ca}}^{2+}\right]}_{\text{total}}+{\left[\text{Casq}\right]}_{\text{total}}+K_{\text{d}}\}[\text{Casq}\\ -{\text{Ca}}^{2+}]+[\text{Casq}-{\text{Ca}}^{2+}{]}^{2})\\ =0 \end{array}$$

For the purpose of solving the quadratic equation of the form $ax^{2}+bx+c=0$,

(45) $$\begin{array}{c} a=1\\ b=\;-\{{\left[{\text{Ca}}^{2+}\right]}_{\text{total}}+{\left[\text{Casq}\right]}_{\text{total}}+K_{\text{d}}\}\\ c={\left[{\text{Ca}}^{2+}\right]}_{\text{total}}{\left[\text{Casq}\right]}_{\text{total}} \end{array}$$

The solution of the quadratic equation is then:

(46) $$[\text{Casq}-{\text{Ca}}^{2+}]=\frac{K_{\text{d}}+{\left[{\text{Ca}}^{2+}\right]}_{\text{total}}+{\left[\text{Casq}\right]}_{\text{total}}-\sqrt{{\left(K_{\text{d}}+{\left[{\text{Ca}}^{2+}\right]}_{\text{total}}+{\left[\text{Casq}\right]}_{\text{total}}\right)}^{2}-4{\left[{\text{Ca}}^{2+}\right]}_{\text{total}}{\left[\text{Casq}\right]}_{\text{total}}}}{2}$$

Taking the discriminant with the negative square root since a positive *D*_Ca_^2+^ requires [Casq-Ca^2+^] ≤ [Casq]_total_ and substituting the root into the equation for $D_{{\text{Ca}}^{2+}}^{*}$:


$$D_{{\text{Ca}}^{2+}}^{*}=\frac{D_{{\text{Ca}}^{2+}}({\left[{\text{Ca}}^{2+}\right]}_{\text{total}}-\frac{K_{\text{d}}+{\left[{\text{Ca}}^{2+}\right]}_{\text{total}}+{\left[\text{Casq}\right]}_{\text{total}}-\sqrt{{\left(K_{\text{d}}+{\left[{\text{Ca}}^{2+}\right]}_{\text{total}}+{\left[\text{Casq}\right]}_{\text{total}}\right)}^{2}-4{\left[{\text{Ca}}^{2+}\right]}_{\text{total}}{\left[\text{Casq}\right]}_{\text{total}}}}{2})}{{\left[{\text{Ca}}^{2+}\right]}_{\text{total}}}$$

$$=\frac{D_{{\text{Ca}}^{2+}}(2{\left[{\text{Ca}}^{2+}\right]}_{\text{total}}-(K_{\text{d}}+{\left[{\text{Ca}}^{2+}\right]}_{\text{total}}+{\left[\text{Casq}\right]}_{\text{total}}-\sqrt{{\left(K_{\text{d}}+{\left[{\text{Ca}}^{2+}\right]}_{\text{total}}+{\left[\text{Casq}\right]}_{\text{total}}\right)}^{2}-4{\left[{\text{Ca}}^{2+}\right]}_{\text{total}}{\left[\text{Casq}\right]}_{\text{total}}}))}{2{\left[{\text{Ca}}^{2+}\right]}_{\text{total}}}$$

(47) $$=\frac{{\text{D}}_{{\text{Ca}}^{2+}}({\left[{\text{Ca}}^{2+}\right]}_{\text{total}}-K_{\text{d}}-{\left[\text{Casq}\right]}_{\text{total}}+\sqrt{{\left(K_{\text{d}}+{\left[{\text{Ca}}^{2+}\right]}_{\text{total}}+{\left[\text{Casq}\right]}_{\text{total}}\right)}^{2}-4{\left[{\text{Ca}}^{2+}\right]}_{\text{total}}{\left[\text{Casq}\right]}_{\text{total}}}))}{2{\left[{\text{Ca}}^{2+}\right]}_{\text{total}}}$$

Equation 14 gives the corresponding value of [Ca^2+^]_free_ in terms of [Ca^2+^]_total_, *K*_d_, and [Casq]_total_:

(14a) $$K_{\text{d}}=\frac{{\left[{\text{Ca}}^{2+}\right]}_{\text{free}}({\left[\text{Casq}\right]}_{\text{total}}-{\left[{\text{Ca}}^{2+}\right]}_{\text{total}}+{\left[{\text{Ca}}^{2+}\right]}_{\text{free}})}{{\left[{\text{Ca}}^{2+}\right]}_{\text{total}}-{\left[{\text{Ca}}^{2+}\right]}_{\text{free}}}$$

Expanding this to:

(48) $$\begin{array}{l} K_{d}{\left[{\text{Ca}}^{2+}\right]}_{\text{total}}-K_{d}{\left[{\text{Ca}}^{2+}\right]}_{\text{free}}\\ ={\left[{\text{Ca}}^{2+}\right]}_{\text{free}}({\left[\text{Casq}\right]}_{\text{total}}-{\left[{\text{Ca}}^{2+}\right]}_{\text{total}})+[{\text{Ca}}^{2+}{]}_{\text{free}}^{\;2} \end{array}$$

Gives the quadratic equation:

(49) $${\left[{\text{Ca}}^{2+}\right]}_{\text{free}}^{2}+{\left[{\text{Ca}}^{2+}\right]}_{\text{free}}({\left[\text{Casq}\right]}_{\text{total}}-{\left[{\text{Ca}}^{2+}\right]}_{\text{total}}+K_{\text{d}})-K_{\text{d}}{\left[{\text{Ca}}^{2+}\right]}_{\text{total}}=0$$

With coefficients:

(50) $$\begin{array}{c} a=1\\ b=({\left[\text{Casq}\right]}_{\text{total}}-{\left[{\text{Ca}}^{2+}\right]}_{\text{total}}+K_{\text{d}})\\ c=-K_{\text{d}}{\left[{\text{Ca}}^{2+}\right]}_{\text{total}} \end{array}$$

Taking the discriminant with the positive square root, in order for [Ca^2+^]_free_ to be positive, yields the following expression for ${\left[{\text{Ca}}^{2+}\right]}_{\text{free}}$:

(51) $$\begin{array}{l} {{\text{[Ca}}^{2+}]}_{\text{free}}\\ =\frac{-K_{\text{d}}+{{\text{[Ca}}^{2+}]}_{\text{total}}-{\left[\text{Casq}\right]}_{\text{total}}+\sqrt{{\left(K_{\text{d}}-{\text{ [Ca}}^{2+}{]}_{\text{total}}+{\left[\text{Casq}\right]}_{\text{total}}\right)}^{2}+4{\text{ [Ca}}^{2+}{]}_{\text{total}}K_{\text{d}}}}{2} \end{array}$$

The consistency of the computations above could be checked by two complementary determinations. The first determined [Ca^2+^]_total_ where the SR Ca^2+^ store contained no calsequestrin for which the total Ca^2+^ is exclusively free Ca^2+^: ${\left[{\text{Ca}}^{2+}\right]}_{\text{total}}^{\left(0,\text{ t}\right)}$ = ${\left[{\text{Ca}}^{2+}\right]}_{\text{free}}^{\left(0,\text{ t}\right)}$. The second determined [Ca^2+^]_free_ in the situation in which calsequestrin was present so that the total Ca^2+^ comprised both bound and free Ca^2+^, for comparisons with the derived values obtained above.

## 3 Results

### 3.1 Color map portrayals of spatial and temporal patterns of free and total SR [Ca^2+^] over <20-ms intervals following the onset of SR Ca^2+^ efflux

**Table 1** summarizes the employed, referenced, parameter values defining (i) the geometry of the sarcoplasmic reticulum (SR) element, (ii) the initial and (iii) the boundary conditions established in the “Theory” section, and (iv) the time and spatial resolutions used in the MATLAB computations. The computations first determined diffusional free, [Ca^2+^]_free_, and total, [Ca^2+^]_total_, Ca^2+^ concentration patterns, at early, <20 ms, times. These were followed from the onset of both constant $J_{\text{efflux}}^{\left(t\right)}=\;J_{\text{efflux}}^{\left(0\right)}\;$and decaying CSR Ca^2+^ release fluxes $J_{\text{efflux}}^{\left(t\right)}$ driven by and accordingly falling from previously reported experimental values (Bardsley et al., 2021; Kovacs et al., 1983) with the consequently declining free cisternal (CSR) [Ca^2+^]_free_. The simulation conditions included the absence and presence, respectively, of Ca^2+^ buffering by established mean total SR calsequestrin concentrations, [Casq] (Volpe and Simon, 1991). They also investigated the effects of background SR Ca^2+^–ATPase (SERCA)-mediated Ca^2+^ re-uptake (Bardsley et al., 2021).

**Figure 2:**
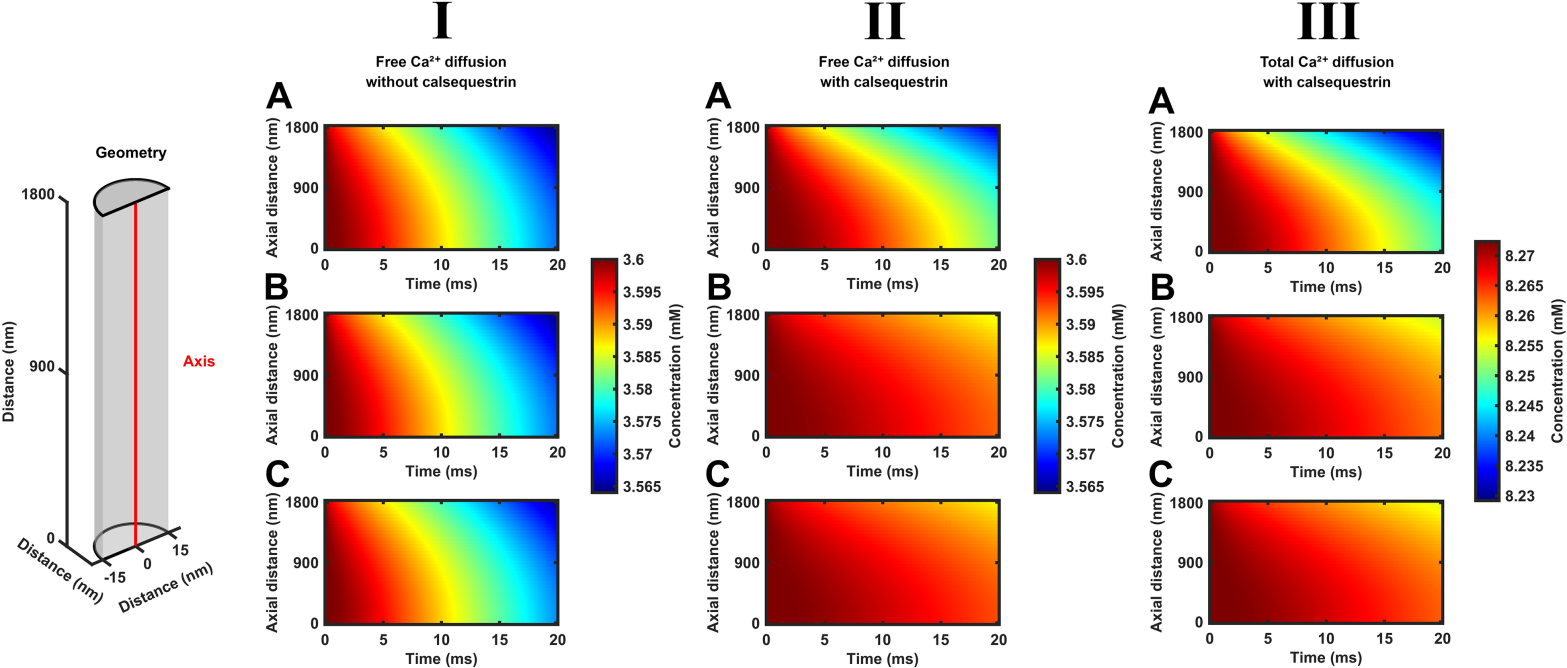
Time-dependent free and total Ca^2+^ diffusion concentration gradients along the length of the SR element through early times during SR Ca^2+^ efflux. Color maps summarizing computational results displaying SR free, [Ca^2+^]_free_, and total Ca^2+^ concentrations, [Ca^2+^]_total_, at different, equally spaced, longitudinal positions along the red line in the formalized SR geometry (inset) at different early times, *t* < 20 ms, following the onset of SR RyR1-mediated Ca^2+^ effluxes and SERCA-mediated influxes. These run from the center of the longitudinal SR, LSR at *l* = 0 nm, to the cisternal SR, CSR, *l* = 180 nm. Studies performed for modeled SR in the absence (I) and the presence (II, III) of previously reported SR calsequestrin concentrations uniformly distributed through the SR, [Casq], and its Ca^2+^ binding, *K*_d_, properties. Results are shown for [Ca^2+^]_free_ (I, II) and [Ca^2+^]_total_ (III) under conditions where the RyR1-mediated SR efflux density $J_{\text{efflux}}^{\left(t\right)}$ remains constant through **(A)** or decays from its initial value $J_{\text{efflux}}^{\left(0\right)}$with the consequent cisternal Ca^2+^ depletion **(B, C)** in the absence **(A, B)** and presence of SERCA-mediated Ca^2+^ influx $J_{\Delta \text{SR influx}}^{\left(t\right)}$ **(C)**.

**Figure 3:**
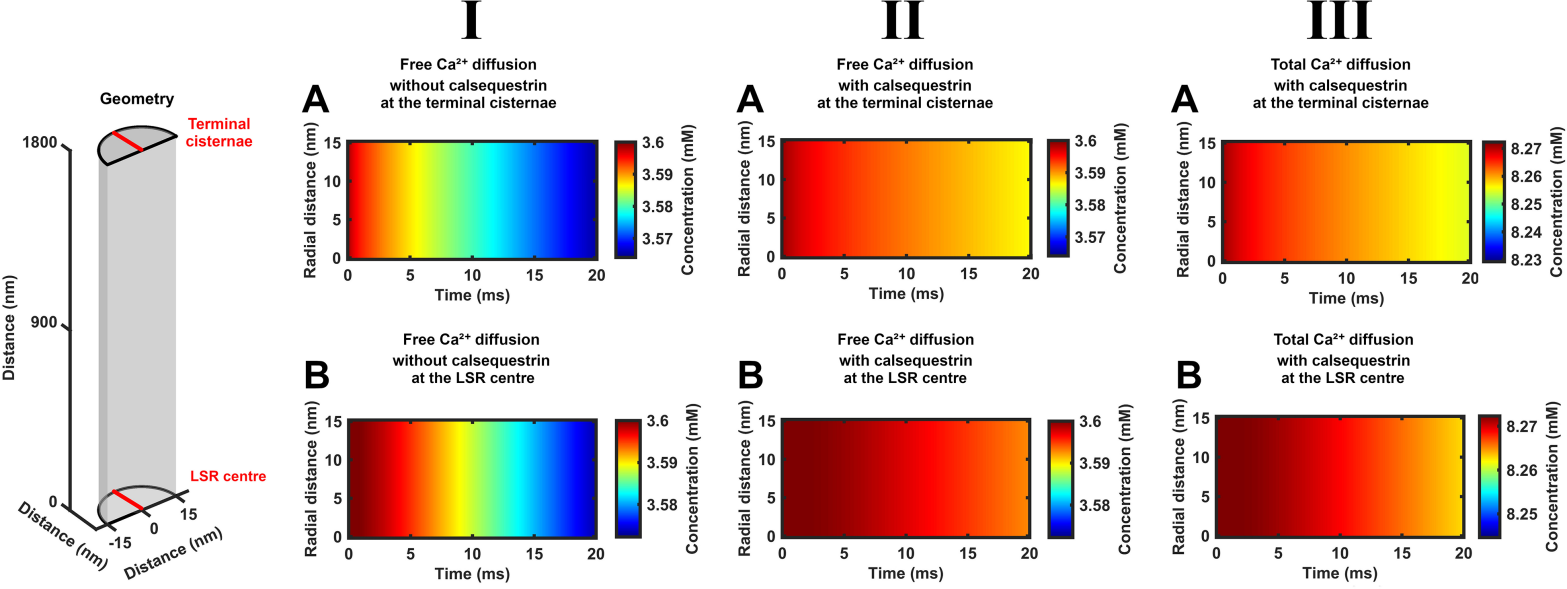
Time-dependent free and total Ca^2+^ concentrations through the radius of each SR element at both the CSR and LSR during SR Ca^2+^ efflux. Color maps summarizing computational results showing SR free, [Ca^2+^]_free_, and total Ca^2+^ concentrations, [Ca^2+^]_total_, through the radius (vertical axis) of the cisternal, CSR **(A)** and center of the longitudinal, LSR, element **(B)** indicated by red lines in the formalized SR geometry (inset) at different early times, *t* < 20 ms (horizontal axis), following the onset of CSR Ca^2+^ effluxes and the presence of SERCA-mediated background Ca^2+^ influxes. The results shown are for [Ca^2+^]_free_ (I, II) and [Ca^2+^]_total_ (III) under conditions where Ca^2+^ efflux at the CSR $J_{\text{efflux}}^{\left(t\right)}$ decays from its initial value $J_{\text{efflux}}^{\left(0\right)}$ with the consequent cisternal Ca^2+^ depletion in the presence of SERCA-mediated Ca^2+^ influx $J_{\Delta \text{SR influx}}^{\left(t\right)}$.

**Figure 4:**
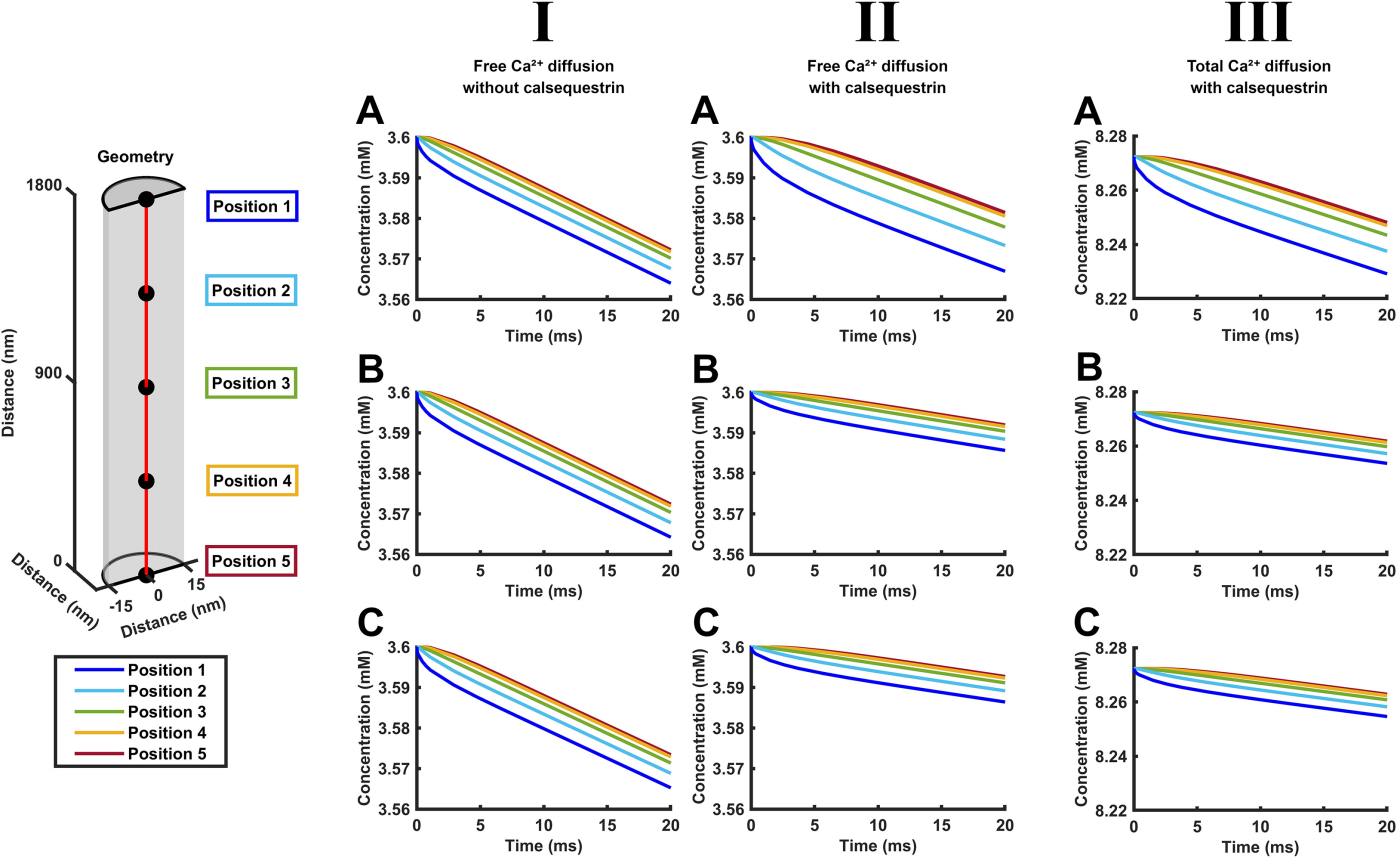
Quantification of the time courses of free and total Ca^2+^ concentration changes along the length of the SR element through early times during SR Ca^2+^ release. Time dependences of free [Ca^2+^]_free_ (I, II) and total Ca^2+^ concentrations [Ca^2+^]_total_ (III) in the absence (I) and the presence (II, III) of calsequestrin. These are shown under conditions where CSR Ca^2+^ efflux $J_{\text{efflux}}^{\left(t\right)}$ remains constant through **(A)** or decays from its initial value $J_{\text{efflux}}^{\left(0\right)}$ with the consequent cisternal Ca^2+^ depletion **(B, C)** in the absence **(A, B)** and presence of SERCA-mediated Ca^2+^ influx $J_{\Delta \text{SR influx}}^{\left(t\right)}$ **(C)**. The plots in each panel are color-coded to denote [Ca^2+^]_free_ and [Ca^2+^]_total_ at equally spaced axial positions along the red line in the formalized SR geometry (inset) at different early times, *t* < 20 ms following the onset of CSR Ca^2+^ efflux $J_{\text{efflux}}^{\left(t\right)}$ in the presence and absence of calsequestrin-mediated Ca^2+^ buffering and of background SERCA-mediated Ca^2+^ influxes $J_{\Delta \text{SR influx}}^{\left(t\right)}$.

**Figure 5:**
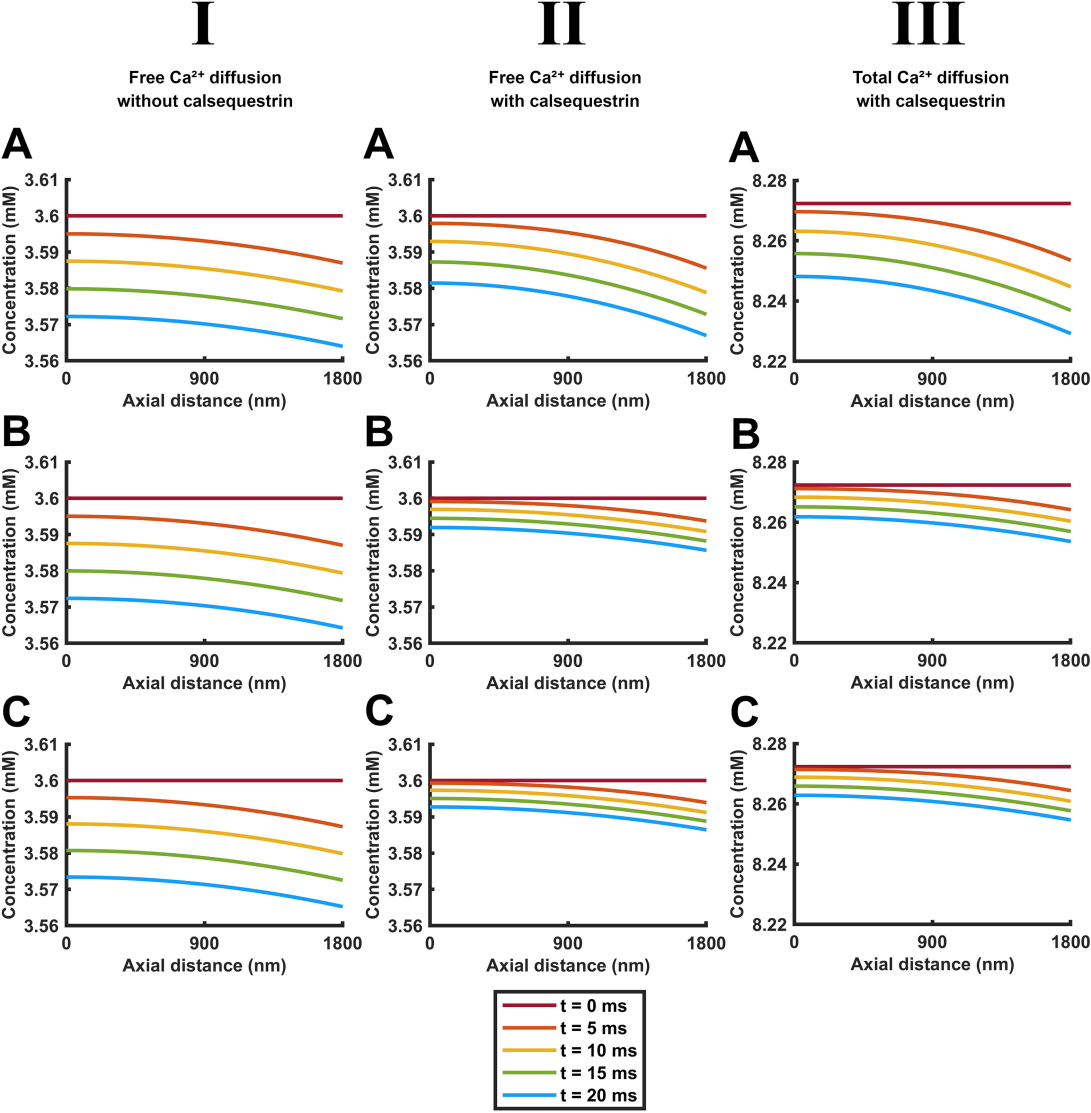
Quantification of the positional dependence of free and total Ca^2+^ concentrations along the length of the SR element at different early times during SR Ca^2+^ release. Spatial dependences of free, [Ca^2+^]_free_, (I, II), and total Ca^2+^ concentrations [Ca^2+^]_total_ (III). These are shown under conditions of constant SR Ca^2+^ efflux $J_{\text{efflux}}^{\left(t\right)}$ **(A)** or one that decays from its initial value $J_{\text{efflux}}^{\left(0\right)}$ with the consequent cisternal Ca^2+^ depletion **(B, C)** in the absence **(A, B)** and presence of SERCA-mediated Ca^2+^ influx $J_{\Delta \text{SR influx}}^{\left(t\right)}$ **(C)**. The plots in each panel are color-coded to denote [Ca^2+^]_free_ and [Ca^2+^]_total_ at equally spaced longitudinal positions along the red line along the SR axis (inset) at different early times, *t* < 20 ms following the onset of CSR Ca^2+^ effluxes $J_{\text{efflux}}^{\left(t\right)}\;$ and SERCA-mediated influxes $J_{\Delta \text{SR influx}}^{\left(t\right)}$.

The color maps in **Figures 2** and **3** represent SR [Ca^2+^]_free_ and [Ca^2+^]_total_ findings with position, *x*, along the SR axial length (**Figure 2**) and radius (**Figure 3**), along the vertical axis and in time *t*, along the horizontal axis. The color code variations along the vertical axis accordingly represent spatial and those along the horizontal axis represent temporal [Ca^2+^]_free_ or [Ca^2+^]_total_ variations. All of the color maps demonstrate a development of significant gradients in both [Ca^2+^]_free_ and [Ca^2+^]_total_ running from the LSR center to the CSR. The [Ca^2+^] color map portraits along the SR axis (**Figure 2**), and their detailed graphical plots (**Figures 4**, **5**) are shown for both constant Ca^2+^ flux (A) and decaying (B, C) Ca^2+^ fluxes in the absence and presence of calsequestrin ((I) and (II)) or of SERCA background activity ((A, B) and (C)). This display demonstrated relatively similar results *within* each *column* (I–III) at each position in **Figure 2**. This suggested similar results from modeling constant or decaying CSR Ca^2+^ effluxes or of including or excluding background SERCA activity within the examined time interval. Contrastingly, differing color maps between rows (A–C) suggested significant effects of including or excluding calsequestrin with the maps. These indicated more persistent high levels of both [Ca^2+^]_free_ and [Ca^2+^]_total_ when including calsequestrin.

**Figure 3** explores for the presence or absence of SR [Ca^2+^]_free_ and [Ca^2+^]_total_ heterogeneities through the radius rather than the length of each SR element. It illustrates computational findings following the onset of declining CSR Ca^2+^ fluxes through an SR cross-section located at the cisternal and the center of the longitudinal SR, CSR and LSR. It compares [Ca^2+^]_free_ ((I), (II)) and [Ca^2+^]_total_, (III) in the absence (I) and the presence of calsequestrin ((II) and (III), through the radius of the CSR and LSR ((**A**) and (B) respectively) in the presence of SERCA activity.

The observed [Ca^2+^]_free_ and [Ca^2+^]_total_ corroborate the corresponding findings along the axial SR with time at the appropriate SR positions—for example, they confirm the more rapid declines in both [Ca^2+^]_free_ and [Ca^2+^]_total_ at the CSR compared to the LSR. However, the color maps indicate no radial nonuniformities in either [Ca^2+^]_free_ and [Ca^2+^]_total_ with time. Quantifications of these radial [Ca^2+^] maps suggested that axial exceeded radial concentration differences by >3 orders of magnitude. This is consistent with expectations for the small SR radial element (30 nm) diameters relative to the SR lengths. Thus, both SR Ca^2+^ diffusion within and its efflux from the SR are essentially a one-dimensional diffusion process. Hence, the axial [Ca^2+^] portrayals represented a homogeneous [Ca^2+^] through the cross-section of each SR element in the absence of radial as opposed to axial [Ca^2+^] gradients.

### 3.2 Quantification of [Ca^2+^]_free_ and [Ca^2+^]_total_ at selected axial SR positions <no><20</no> ms following the onset of CSR Ca^2+^ efflux

**Figures 4** and **5** quantify the abovementioned color map portrayals of diffusional [Ca^2+^]_free_ and [Ca^2+^]_total_ patterns under the different conditions adopted for SR Ca^2+^ efflux, intra-SR Ca^2+^ diffusion, calsequestrin-mediated Ca^2+^ buffering, and SERCA-mediated Ca^2+^ re-uptake over the <20-ms interval adopted above. By displaying a smaller Ca^2+^ concentration range, the detailed [Ca^2+^] plots more closely demonstrate the [Ca^2+^]_free_ and [Ca^2+^]_total_ differences. **Figure 4** provides detailed plots of axial [Ca^2+^]_free_ and [Ca^2+^]_total_ versus time at different color-coded points along the SR axis (inset). These likely represent concentrations along a homogeneous diffusion front in view of the absence of demonstrable radial heterogeneities in either [Ca^2+^]_free_ or [Ca^2+^]_total_ (**Figure 3**). All of the plots showed similar initial respective [Ca^2+^]_free_ and [Ca^2+^]_total_ values at all of the SR positions as expected from the initial conditions. Both [Ca^2+^]_free_ and [Ca^2+^]_total_ then declined with time as expected from the Ca^2+^ depletion resulting from SR Ca^2+^ efflux. Whether under conditions of both constant (**Figure 4A**) and decaying SR Ca^2+^ efflux (**Figures 4B, C**), calsequestrin attenuated the decline in [Ca^2+^]_free_ at all of the points along the SR axis (compare I and II). It also reduced the fall in [Ca^2+^]_free_ with distance from LSR to the CSR. As expected from a simple and instantaneous Ca^2+^ buffering, the [Ca^2+^]_total_ profiles resembled those of [Ca^2+^]_free_, allowing for a normalization of these values reflecting Ca^2+^ binding to calsequestrin (compare (II) and (III)). Calsequestrin buffering exerted concordant but more marked effects under conditions of a decaying as opposed to constant SR Ca^2+^ efflux. Calsequestrin-mediated Ca^2+^ buffering thus conserves not only [Ca^2+^]_total_ but also [Ca^2+^]_free_ at all points along the length of the SR. In contrast, the presence or absence of background SERCA activity made little difference to the [Ca^2+^]_free_ and [Ca^2+^]_total_ patterns.

However, detailed time courses of the [Ca^2+^] plots varied with SR position. The CSR showed the most initially (<5 ms) rapid [Ca^2+^]_free_ and [Ca^2+^]_total_ depletion, giving downward convex curves. Toward the LSR end, closer to the sarcomere center (position 5), the falls in [Ca^2+^]_free_ and [Ca^2+^]_total_ were delayed, giving a downward concave curve. These early differences were followed by steady relatively linear and parallel declines in [Ca^2+^]_free_ and [Ca^2+^]_total_ within the 20-ms time course. However, their slopes were reduced in the presence of calsequestrin.

The [Ca^2+^]_free_ and [Ca^2+^]_total_ time courses at different SR positions thus demonstrate declines in [Ca^2+^]_free_ and [Ca^2+^]_total_ from the expected constant initial values. These concentrations show sharper drops in [Ca^2+^]_free_ and [Ca^2+^]_total_ at the CSR than toward the LSR where the latter concentrations persisted through an initial shoulder phase. However, the axial SR [Ca^2+^]_free_ and [Ca^2+^]_total_ gradients then all showed roughly parallel declines in [Ca^2+^]_free_ and [Ca^2+^]_total_, reflecting the length of the SR acting like a diffusional delay line. These general features applied whether either calsequestrin buffering or SERCA activity was absent or present. Nevertheless, the presence of calsequestrin reduces the gradients of the decays, with greater effects in the presence of decaying rather than constant SR Ca^2+^ effluxes. However, the presence or absence of background SERCA activity does not affect either the delays or the slopes.

### 3.3 Quantification of [Ca^2+^]_free_ and [Ca^2+^]_total_ with axial SR position at selected <20-ms times following the onset of CSR Ca^2+^ efflux

**Figure 5** complements **Figure 4** in plotting [Ca^2+^]_free_ (I) and [Ca^2+^]_total_ (II; vertical axis) along the SR axis (horizontal) at different early, <20 ms, color-coded times following the onset of CSR Ca^2+^ efflux. It similarly compares results from constant (A) and decaying SR Ca^2+^ effluxes (B,C) in the absence (I) or presence of calsequestrin (II, III) in the absence (B) or presence of background SERCA activity (C). All these conditions yielded families of close to parallel [Ca^2+^]_free_ and [Ca^2+^]_total_ plots against position through the different timesteps. The plots successively declined from constant initial [Ca^2+^]_free_ and [Ca^2+^]_total_ along the SR length. Each curve displayed constant and parallel [Ca^2+^]_free_ and [Ca^2+^]_total_ toward the LSR end, reflecting a buffering effect of the elongated SR. But it then fell with distance toward the CSR reflecting the diffusive changes following the SR Ca^2+^ efflux. This gave profiles with a concave downward distribution. As expected from the adopted calsequestrin buffering properties, the buffered [Ca^2+^]_free_ and [Ca^2+^]_total_ curves were similar in form, apart from the latter showing greater absolute magnitudes. The presence of calsequestrin buffering also reduced the successive decline in value of the [Ca^2+^]_free_-SR distance curves with time and resulted in expectedly overall higher values of [Ca^2+^]_total_. The presence of background SERCA activity showed little effects on these families of [Ca^2+^]_free_ and [Ca^2+^]_total_–SR distance curves.

These complementary plots thus demonstrate that the SR length acts like a buffer, maintaining relatively constant values of [Ca^2+^]_free_ and [Ca^2+^]_total_ particularly toward the LSR, at the different times. [Ca^2+^]_free_ and [Ca^2+^]_total_ only dip toward the CSR, doing so with roughly parallel declines. This is the case whether calsequestrin was absent or present and whether SERCA activity was absent or present. However, the presence or absence of calsequestrin affected the values and the closeness of these levels at different times; those of background SERCA activity did not make any difference to either the delays or the slopes.

### 3.4 Color map portrayals of spatial and temporal patterns of free and total SR [Ca^2+^] over prolonged <2-s intervals following the onset of SR Ca^2+^ efflux

**Figure 6:**
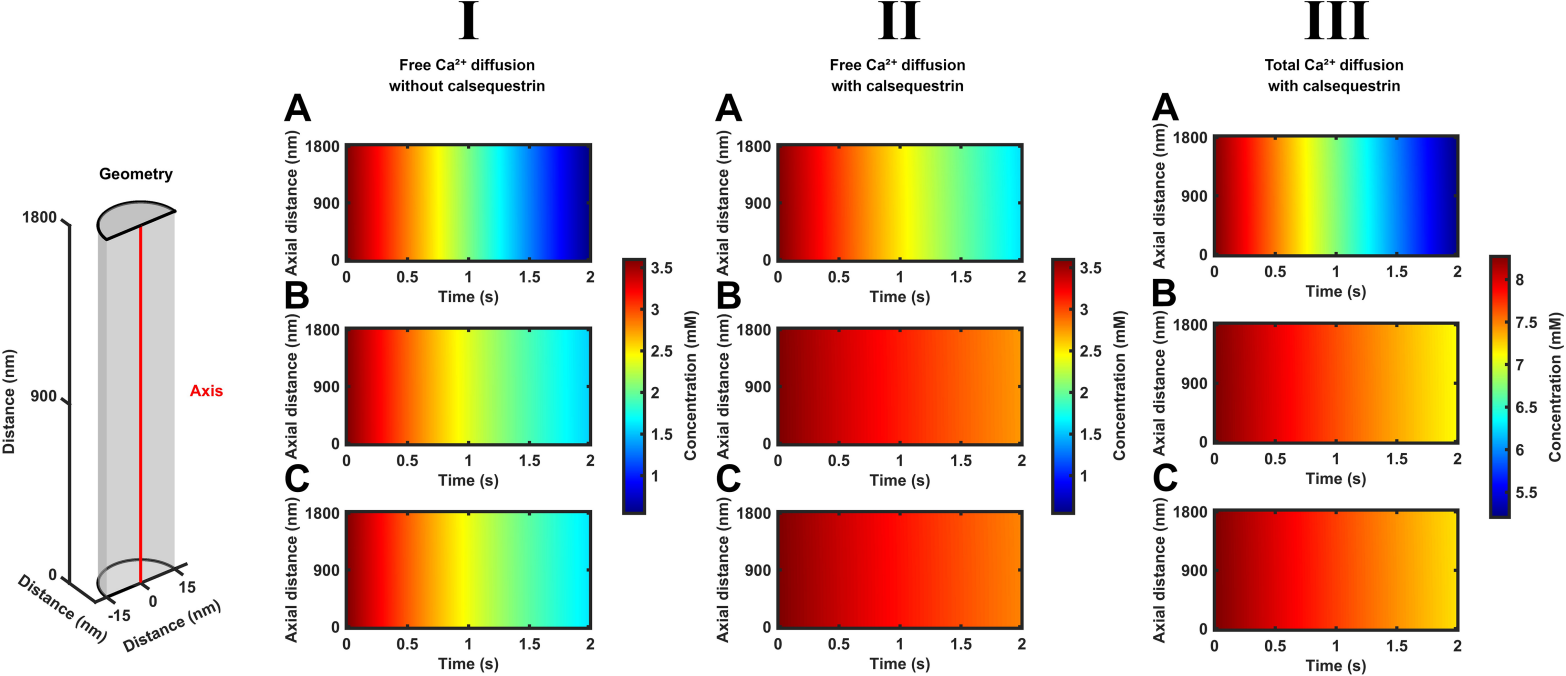
Free and total Ca^2+^ time-dependent diffusion gradients along the SR axis through prolonged times during CSR Ca^2+^ efflux. Color maps of [Ca^2+^]_free_ and [Ca^2+^]_total_ along the SR axis indicated by the red line in the formalized SR geometry (inset) at late times, *t* < 2 s following the onset of the CSR Ca^2+^ efflux $J_{\text{efflux}}^{\left(t\right)}$. Studies in the absence (I) and presence of calsequestrin (II,III). Results shown are for [Ca^2+^]_free_ (I, II) and [Ca^2+^]_total_ (III) under conditions of constant CSR Ca^2+^ efflux $J_{\text{efflux}}^{\left(t\right)}$ = $J_{\text{efflux}}^{\left(0\right)}$ **(A)** and one that decays from its initial value $J_{\text{influx}}^{\left(0\right)}$ with the consequent cisternal Ca^2+^ depletion **(B, C)** in the absence **(A, B)** and presence of SERCA-mediated Ca^2+^ influx $J_{\Delta \text{SR influx}}^{\left(t\right)}$ **(C)**.

**Figure 7:**
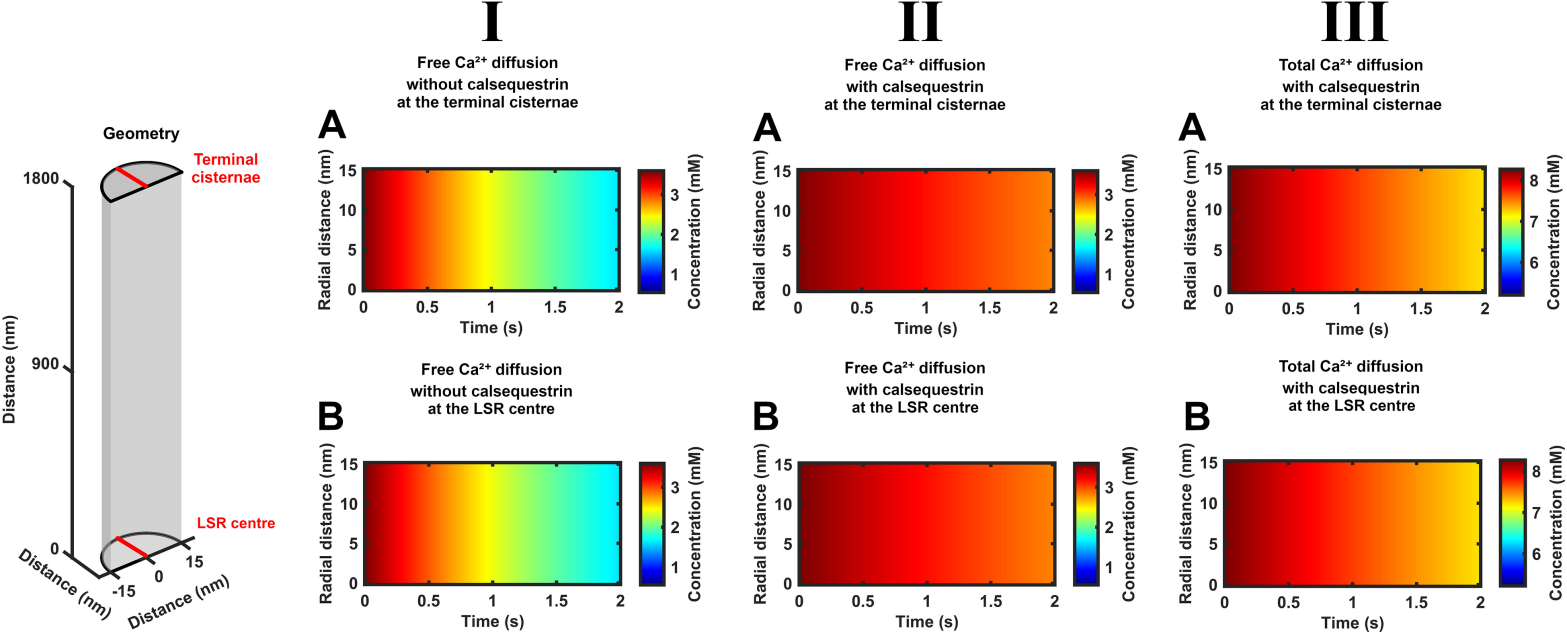
Uniform free and total Ca^2+^ concentrations through the radius of each SR element during SR Ca^2+^ release through prolonged times during CSR Ca^2+^ efflux. Color maps of SR Ca^2+^ concentrations through the radius of the cisternal CSR **(A)** and center of the longitudinal LSR **(B)** indicated by red lines in the formalized SR geometry (inset) at late times, *t* < 2 s following the onset of the CSR Ca^2+^ efflux $J_{efflux}^{\left(t\right)}$. Studies in the absence (I) and presence of calsequestrin (II, III). Results shown are for free [Ca^2+^]_free_ (I, II) and total Ca^2+^ concentrations [Ca^2+^]_total_ (III) under conditions where the CSR efflux $J_{efflux}^{\left(t\right)}$ decays from its initial value $J_{\text{efflux}}^{\left(0\right)}$ with the consequent [Ca^2+^]_free_ and [Ca^2+^]_total_ depletion in the presence of SERCA-mediated Ca^2+^ influx $J_{\Delta \text{SR influx}}^{\left(t\right)}$.

Color map portraits were also obtained exploring the same conditions of SR Ca^2+^ efflux, calsequestrin-mediated SR Ca^2+^ buffering, and SERCA-mediated Ca^2+^ re uptake through longer <2-s intervals, seeking to explore a fuller approach of the system to a steady state. Such durations encompass more prolonged physiological situations such as tetanus (**Figures 6**, **7**). These contrastingly suggested SR Ca^2+^ fluxes occurring in a presence of relatively dissipated diffusional [Ca^2+^]_free_ and [Ca^2+^]_total_ gradients. The portrayals along the SR axis (vertical axis) with time (horizontal axis) now showed vertical rather than sloping color boundaries. This was the case under all of the tested conditions of constant or decaying SR Ca^2+^ efflux and the absence or presence either of calsequestrin or of SERCA activity (**Figure 6**). This indicates spatially uniform [Ca^2+^]_free_ and [Ca^2+^]_total_ diffusion fronts throughout the entire SR length. Similarly, the [Ca^2+^]_free_ and [Ca^2+^]_total_ color maps demonstrated no radial [Ca^2+^]_free_ and [Ca^2+^]_total_ heterogeneities. Furthermore, the images obtained at the two CSR and LSR end positions along the SR axis then were similar (**Figure 7**). This suggested an absence of diffusional concentration gradients along the length of the SR.

**Figure 8:**
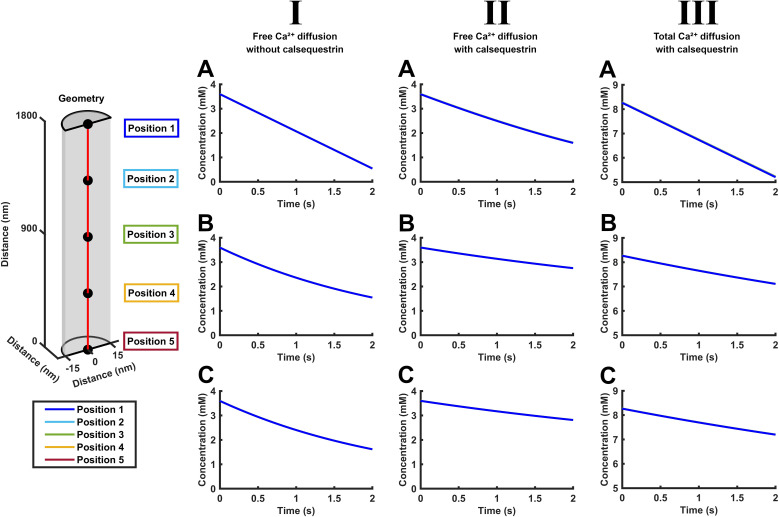
Free and total Ca^2+^ concentrations with time through prolonged intervals (<2 s) following SR Ca^2+^ release at different points along the length of the SR element. Studies in the absence (I) and presence of calsequestrin (II, III). Time dependences of free [Ca^2+^]_free_ (I, II) and total Ca^2+^ concentrations [Ca^2+^]_total_ (III) where CSR Ca^2+^ efflux remains constant $J_{\text{efflux}}^{\left(t\right)}$ = $J_{\text{efflux}}^{\left(0\right)}$ **(A)** or decays from its initial value $J_{\text{efflux}}^{\left(0\right)}$ with the consequent [Ca^2+^]_free_ and [Ca^2+^]_total_ **(B, C)** in the absence **(A, B)** and presence of SERCA-mediated Ca^2+^ influx $J_{\Delta \text{SR influx}}^{\left(t\right)}$**(C)**. Note the overlapping decays at the different SR positions, suggesting diffusion equilibration through the SR axis.

**Figure 9:**
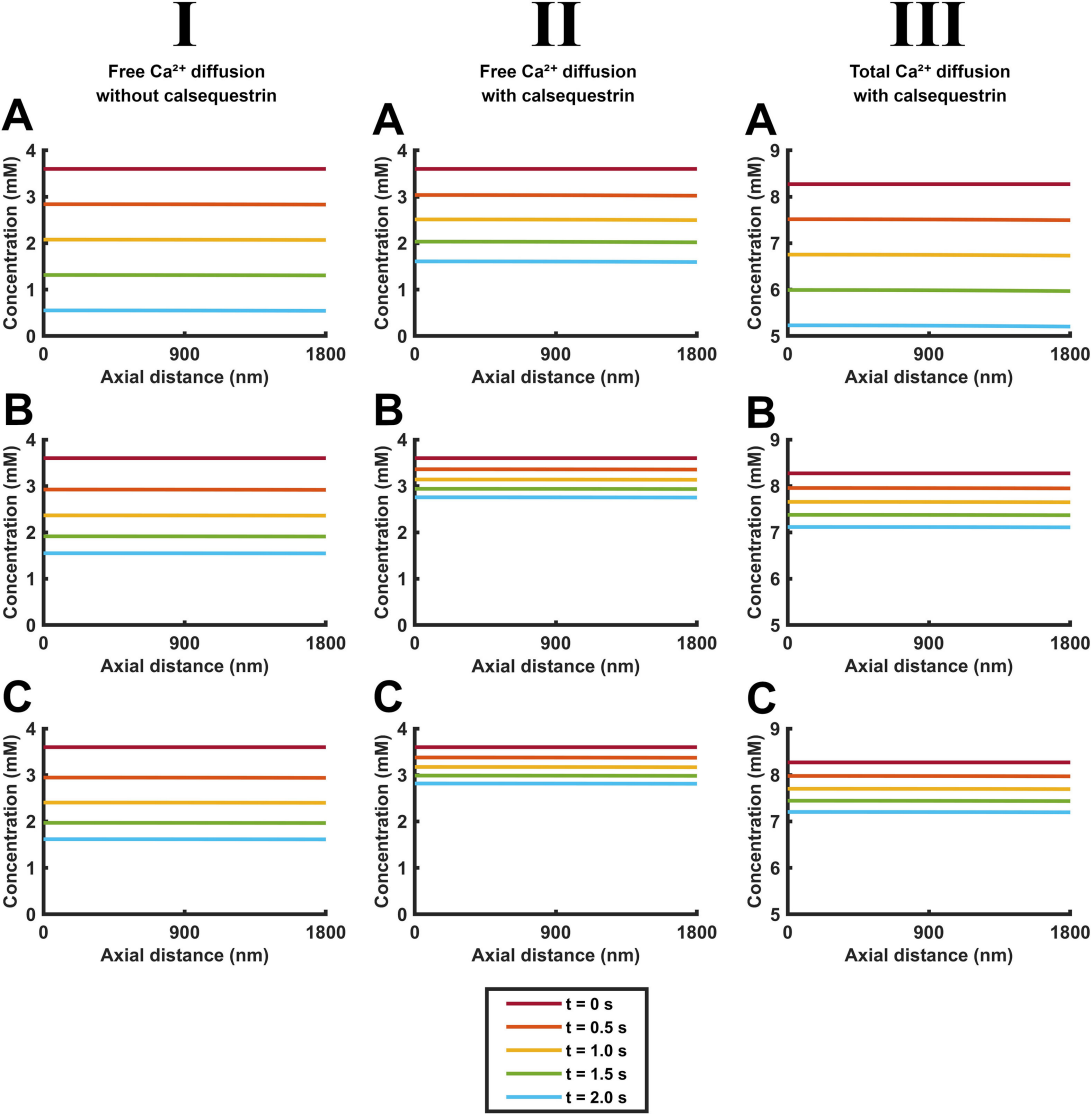
Free and total Ca^2+^ concentrations along the length of the SR element at different late times during SR Ca^2+^ release. Studies in the absence (I) and presence of calsequestrin (II, III). Spatial dependences of free [Ca^2+^]_free_ (I, II) and total Ca^2+^ concentrations [Ca^2+^]_total_ (III) with a constant **(A)** or decaying **(B, C)** CSR Ca^2+^ efflux $J_{\text{efflux}}^{\left(t\right)}$ in the absence **(A, B)** and presence of SERCA-mediated Ca^2+^ influx $J_{\Delta \text{SR influx}}^{\left(t\right)}$ **(C)**. The plots in each panel were color-coded to denote SR Ca^2+^ concentrations at equally spaced axial positions along the red line in the formalized SR geometry (inset) at different late times, *t* < 2 s following the onset of CSR Ca^2+^ effluxes and SERCA-mediated influxes. Note the greater time spacing of the successive plots compared to **Figure 5**.

The detailed plots of both [Ca^2+^]_free_ and [Ca^2+^]_total_ against time obtained at different positions along the SR length were superimposable. Thus, there were similar initial values of [Ca^2+^]_free_ and [Ca^2+^]_total_ at all of the SR positions as well as through their subsequent declines with time. The latter declines were more marked with decaying than with constant SR effluxes. These features suggest minimal diffusion gradients along the SR axis (**Figure 8**). As before, these declines were more rapid at a constant than a decaying SR Ca^2+^ efflux rate. Calsequestrin expectedly resulted in increased values of [Ca^2+^]_total_. It similarly slowed the declines in [Ca^2+^]_free_ and [Ca^2+^]_total_. Plots against distance now demonstrated uniform [Ca^2+^]_free_ and [Ca^2+^]_total_ along the SR length falling in value at successive times, suggesting minimal diffusion gradients (**Figure 9**). It similarly shows that calsequestrin gives rise to expectedly overall higher values of [Ca^2+^]_total_ and slows the declines in [Ca^2+^]_free_ and [Ca^2+^]_total_. These plots were all unaffected by the presence or absence of background SERCA activity.

### 3.5 Quantification of the SR free Ca^2+^ efflux and overall temporal and spatial variations in [Ca^2+^]_free_

**Figure 10:**
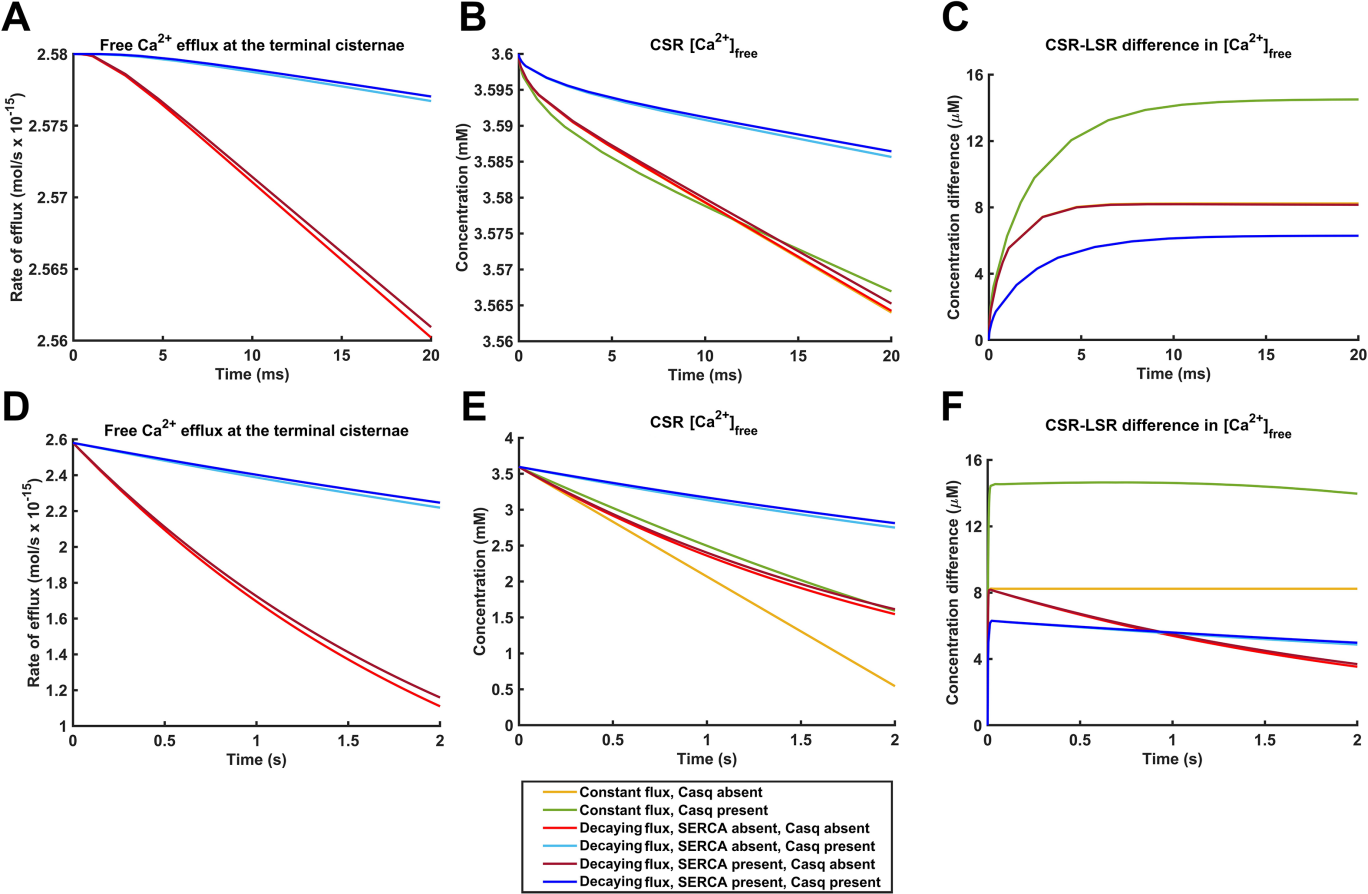
Consequences of the SR diffusion patterns for TSR rates of Ca^2+^ release, its driving CSR free Ca^2+^ concentrations, and axial heterogeneities in free Ca^2+^ concentrations. **(A, D)** Rates of release of free Ca^2+^ into the T–SR junction from the CSR, $J_{\text{efflux}}^{\left(t\right)}\;$and **(B, E)** their driving cisternal [Ca^2+^]_free_ ( ${\left[{\text{Ca}}^{2+}\right]}_{\text{free}}^{\left(0,t\right)}$) and **(C, F)** the consequent differences between CSR and LSR [Ca^2+^]_free_ ( ${\left[{\text{Ca}}^{2+}\right]}_{\text{free}}^{\left(0,t\right)}-{\left[{\text{Ca}}^{2+}\right]}_{\text{cyt}}^{\left(l/2,t\right)}$) with time following the onset of CSR Ca^2+^ efflux. Findings over **(A–C)** the shorter < 20 ms and **(D–F)** the longer < 2 s interval. The findings were compared for conditions of constant Ca^2+^ flux in the presence and absence of calsequestrin and of declining Ca^2+^ flux in the respective presence and absence of calsequestrin and of SERCA activity (color-coded). Note the overlapping lines obtained in the respective cases with and without SERCA activity.

**Figure 10** surveys the consequent SR effluxes of free Ca^2+^ and their corresponding CSR [Ca^2+^]_free_ with time over both the shorter <20-ms (A, B) and the longer <2-s intervals (D, E) following the onset of CSR Ca^2+^ efflux. In addition, the concentration or time increments (mM and s, respectively) in the previous plots of [Ca^2+^]_free_ and [Ca^2+^]_total_ against time (**Figure 8**) or SR distance (**Figure 9**) were insufficient to resolve the small [Ca^2+^]_free_ and [Ca^2+^]_total_ heterogeneities along the SR axis. Accordingly, **Figure 10C, F** estimate the latter as CSR–LSR [Ca^2+^]_free_ differences with time with an expanded (μM) scale. **Figure 10** compares the results of constant and declining SR Ca^2+^ effluxes in the respective presence and absence of calsequestrin and of SERCA activity. Under all such conditions, the free SR Ca^2+^ fluxes (A, D) and CSR [Ca^2+^]_free_ declined with time (B, E) following the onset of CSR Ca^2+^ efflux. However, these declines were less marked in the presence than in the absence of calsequestrin over both the long and the short time intervals. Over the shorter <20-ms interval, the CSR Ca^2+^ efflux declined by ~0.8% in the absence and a markedly reduced 0.15% in the presence of calsequestrin. These parallelled reductions in CSR [Ca^2+^]_free_ of ~0.9% and 0.13%, respectively. These built up positive overall LSR–CSR [Ca^2+^]_free_ differences of ~14, 8, and 6 μM under conditions, respectively, of a constant SR Ca^2+^ efflux in the presence of calsequestrin and declining SR Ca^2+^ effluxes in the absence and presence of calsequestrin. Overlapping plots were obtained in the presence and absence of SERCA activity.

Over the longer <2-s time interval, the CSR Ca^2+^ efflux showed more marked ~54% and 15% reductions in the absence and presence of calsequestrin, with respective reductions of ~57% and 22% in CSR [Ca^2+^]_free_. These built up small positive overall LSR–CSR [Ca^2+^]_free_ differences of ~8, 8, and 6 μM under conditions, respectively, of a constant SR Ca^2+^ efflux in the presence of calsequestrin and declining SR Ca^2+^ effluxes in the absence and presence of calsequestrin. Overlapping plots were obtained again in the presence and absence of SERCA activity. When calsequestrin was absent, there was a more noticeably declining LSR–CSD difference with time.

## 4 Discussion

The present study uses modeling methods to explore the limiting properties of *intra*-SR Ca^2+^ diffusion, under conditions simulating those of the physiological SR release of activator Ca^2+^. It complements studies of diffusional fluxes and the effects on these of calmodulin-mediated Ca^2+^ buffering following Ca^2+^ release into the TSR junction itself during amphibian skeletal muscle excitation–contraction coupling (Bardsley et al., 2021; Rodríguez et al., 2024). It developed a simplified model of SR intraluminal Ca^2+^ diffusion and Ca^2+^–calsequestrin buffering following the TSR Ca^2+^ release initiating excitation–contraction coupling. This modeling process first required a realistic, nevertheless computationally implementable, formal representation simplifying the structure of the intricate SR membrane network. This provided geometric boundaries within which the simulated diffusion processes took place. The required quantitative anatomical data were derived from previous electron microscopy quantifications in amphibian skeletal muscle (Birks and Davey, 1969; Mobley and Eisenberg, 1975; Peachey, 1965). The latter had provided a formalized but nevertheless anatomically realistic division of the SR into individual parallel longitudinal, LSR, elements with directly measured individual diameters (Peachey, 1965). In the present analysis, their summed individual cross-sectional areas equaled the observed effective SR diameter, itself derived from the previously reported SR fractional, normalized to the total, cell volume. It emerged with a geometry comprising regions of cisternal SR adjacent to the TSR spaces and T-tubular membrane (Franzini-Armstrong, 1970) connected to strands of elongated 15-nm radius, 1,800-nm, longitudinal SR (LSR), extending along the fiber axis to join its opposite symmetric component at the center of the sarcomere length (Peachey, 1965).

The boundary conditions for the conducted computational runs involved step CSR membrane permeability constant changes for free Ca^2+^ corresponding to reported experimental maximum rates of RyR-mediated cytosolic [Ca^2+^] increases following imposed voltage steps (Bardsley et al., 2021; Kovacs et al., 1983; Rodríguez et al., 2024). Such SR efflux rates, localized to the CSR, were either held constant or decayed with loss of their driving forces arising from the consequent SR [Ca^2+^]_free_ depletion. The computations adopted previously reported initial values of SR [Ca^2+^]_free_ and its free diffusion coefficients *D*_Ca2+_. They were performed by comparing findings from a presence or absence, respectively, of previously reported total concentrations, [Casq]_total_, of calsequestrin and its Ca^2+^ buffering constant *K*_d_ (Volpe and Simon, 1991; Ziman et al., 2010). The latter incorporated modified previously developed Ca^2+^ buffering formulations, including buffering effects on the effective Ca^2+^ diffusion coefficients (Rodríguez et al., 2024). Computations were performed in the presence and absence of background levels of SERCA-mediated Ca^2+^ transport taking place along the length of the longitudinal SR from the cytosol back to the SR lumen. The latter were derived from previously calculated SERCA fluxes corresponding to established values of resting cytosolic free [Ca^2+^] (Bardsley et al., 2021; Blatter and Blinks, 1991; Harkins et al., 1993; Konishi and Watanabe, 1995; Kurebayashi et al., 1993). The consequent fluxes within the SR leading to patterns of both buffered and total [Ca^2+^]_total_ and free [Ca^2+^]_free_ concentrations of Ca^2+^ were then modeled in a finite element analysis employing a classical Fick diffusion equation.

The computational analysis applied to the experimentally characterized SR geometry adopted here yielded color maps and detailed quantifications that yielded a number of physical insights concerning SR Ca^2+^ diffusion and buffering in response to a step function of SR membrane permeability giving rise to SR Ca^2+^ release of SR. Firstly, the modeling (1) predicted an overall rapid establishment of significant diffusional gradients over single-twitch, ms, timescales. Here the underlying Ca^2+^ SR fluxes would have reflected both the Ca^2+^ diffusion and buffering within the SR and the SR Ca^2+^ effluxes. However, (2) the diffusive Ca^2+^ fluxes reached a diffusional equilibration over longer, tetanic, s, timescales. Here the fluxes would have reflected only Ca^2+^ buffering and the SR fluxes. These and the consequent time course of the SR Ca^2+^ effluxes were both (3) significantly modified by calsequestrin buffering but (4) not by the presence or absence of background levels of SERCA activity.

Secondly, the detailed color map representations specifically within the 0–20-ms interval following the onset of CSD SR Ca^2+^ efflux demonstrated (1) an early generation of time evolving [Ca^2+^]_free_ and [Ca^2+^]_total_ axial gradients between the LSR and CSR but (2) an absence of significant radial [Ca^2+^] gradients within such SR elements. Accordingly, (3) the Ca^2+^ movement within the SR during CSR free Ca^2+^ release was therefore essentially a one-dimensional diffusive process along the SR axis. In the detailed graphical quantifications, (4) both [Ca^2+^]_free_ and [Ca^2+^]_total_ expectedly assumed similar initial values through the different SR axial positions as expected for initial axially uniform [Casq] and SERCA activity. However, (5) both [Ca^2+^]_free_ and [Ca^2+^]_total_ at each SR axial position then decayed ultimately with similar slopes with the onset of SR Ca^2+^ efflux. (6) The inclusion of [Casq] terms markedly increased the initial absolute values of both [Ca^2+^]_free_ and [Ca^2+^]_total_. However, importantly, (7) it markedly reduced their parallel rates of decay. (8) Similar results arose under conditions of a constant or a declining SR efflux at such <20-ms intervals or in the presence or absence of background SERCA activity.

Thirdly, modeling extended to longer <2 s, tetanic, timescales similarly revealed declining SR [Ca^2+^]_free_, [Ca^2+^]_total_, and SR Ca^2+^ fluxes in the presence or absence of calsequestrin and/or SERCA activity. However, in contrast to findings at the shorter <20-ms intervals, (1) both axial and radial SR [Ca^2+^]_free_, [Ca^2+^]_total_ gradients were now greatly reduced or absent. Thus, Ca^2+^ fluxes through the SR were accordingly now determined primarily by the Neumann boundary condition for release efflux. Hence, diffusional equilibrium within the SR was achieved at these later time intervals. Furthermore, (2) conditions of non-constant SR Ca^2+^ efflux now resulted in slower Ca^2+^ depletion rates than did a constant SR Ca^2+^ efflux. Nevertheless, (3) inclusion of calsequestrin buffer, expected to reduce the effective Ca^2+^ diffusion coefficient and slow down Ca^2+^ diffusion along the length of the SR, continued to attenuate the decays in [Ca^2+^]_free_ and [Ca^2+^]_total_. This would be consistent with the Ca^2+^ buffering effectively acting as a Ca^2+^ store tending to preserve the SR Ca^2+^ efflux. (4) These findings were not significantly altered in computational runs incorporating SERCA-mediated influx into the SR despite an expected action in slowing down the decline of Ca^2+^ along the length of the SR.

Fourthly, surveys of the consequent TSR free Ca^2+^ release, declines in CSR [Ca^2+^]_free_, and the overall nonuniformities in axial SR [Ca^2+^]_free_ demonstrated the expected declining TSR membrane Ca^2+^ fluxes with time at both the shorter <20-ms and longer <2-s intervals. The TSR free Ca^2+^ release expectedly paralleled falls in CSR [Ca^2+^]_free_. Under conditions of decaying SR Ca^2+^ release, calsequestrin reduced the decline to extents unaffected by background SERCA activity, therefore tending to maintain a high release efflux. Over short intervals compatible with single-twitch activity, the respective declines (0.8% and 0.15%) and the corresponding fall in CSR [Ca^2+^]_free_ were relatively small. This accompanied progressive increases in CSR–LSR differences in [Ca^2+^]_free_ of ~14, 8, and 6 μM under conditions, respectively, of a constant SR Ca^2+^ efflux in the presence of calsequestrin and a declining SR Ca^2+^ efflux in the absence and presence of calsequestrin. These effects of the Ca^2+^ diffusion along a relatively long SR diffusion distance and calsequestrin-mediated Ca^2+^ buffering effectively provided a SR Ca^2+^ store and thus contributed to sustaining free Ca^2+^ release into the TSR junction through such timescales. Over the longer <2-s time interval which corresponded to a diffusional equilibration of axial SR [Ca^2+^]_free_ and [Ca^2+^]_total_, CSR Ca^2+^ efflux had fallen by ~54% in the absence and by 15% in the presence of calsequestrin, with corresponding reductions in CSR [Ca^2+^]_free_. Calsequestrin buffering continued to sustain the SR Ca^2+^ effluxes which were little affected by background SERCA activity.

These simple characterizations thus provided physically interpretable limiting properties of diffusion patterns within the SR during TSR Ca^2+^ release. They are also useful as a basis for further, more detailed studies as further experimental findings emerge. In addition to gaining more detailed insights into the expected features of the physical features of the intra-SR diffusional processes involved, they could test the predictive features of the model following incorporation of more complex *in vivo* features of the processes of SR Ca^2+^ diffusion and release. Firstly, they could incorporate further details on ion and other transporter distributions. The present description appropriately formalized the respective RyR2–Ca^2+^ release channels and SR Ca^2+^ ATPases to CSR and LSR membrane localizations (Dulhunty et al., 2002; Bravo-Sagua et al., 2020). Secondly, further explorations could model heterogeneous rather than homogeneous calsequestrin distributions and calsequestrin anchoring at junctional SR membranes (Barone et al., 2015; Junket and Sommer, 1979). They could extend the 1:1 calsequestrin–Ca^2+^ dissociation to fuller, Hill equation, co-operative models of calsequestrin binding up to 50 Ca^2+^ ions (Aaron et al., 1984; Beard et al., 2004; Dulhunty, 2006) and include other known SR Ca^2+^ buffers (Conte et al., 2023) and how local junctional SR [Ca^2+^] changes might influence RyR–Ca^2+^ release channels (Wang and Michalak, 2020). The skeletal muscle also expresses inositol 1,4,5-trisphosphate receptors near the neuromuscular junction nicotinic acetylcholine receptors and peripheral SR membrane (Volpe et al., 2022). However, these are likely involved with regulatory effects rather than direct excitation–contraction coupling (Blaauw et al., 2012). Thirdly, the flux computations could adopt more detailed electrochemical as opposed to simple diffusion models, including, albeit small, potential electric fields built through the flux processes (Bardsley et al., 2021; Rodríguez et al., 2024).

Fourthly, future availabilities of more detailed geometric quantifications of comparative SR geometry could permit extended analyses to other slow oxidative and fast glycolytic skeletal as well as cardiac muscle types (Picht et al., 2011), including mammalian (Rudolf et al., 2006; Ziman et al., 2010) in addition to amphibian muscle types (Baylor and Hollingworth, 2007). It could then be possible to relate the findings to their varying capacities for both short-term bursts and longer-term sustained releases of Ca^2+^.

## 5 Materials and method*s*

This study used MATLAB (Version 24.2, R2024b, update 1), Partial Differential Equation Toolbox (later referred as PDE Toolbox), to solve the differential equations. The program (see Supplementary Material) was run on MacBook Pro 2021 with Apple M1 Pro chip and a 16 GB memory. The detailed equations underlying the model and adaptations of the software to solve the resulting differential equations are described in the “Theory” section. Computational runs were performed to model diffusional events following step initiations of CSR Ca^2+^ efflux over intervals of <20 ms encompassing the typical time course of a single twitch and <2 s similar to timescales of a tetanus (2 s).

## Data Availability

The raw data supporting the conclusions of this article will be made available by the authors, without undue reservation.
