# Supplementary material for: Finite element modeling of sarcoplasmic reticular intraluminal Ca2+ diffusional fluxes during amphibian striated muscle excitation–contraction coupling
Source: Front Physiol. 2026 Apr 28;17:1774977. doi: 10.3389/fphys.2026.1774977 (PMC13160737; doi:10.3389/fphys.2026.1774977)
Supplement: Supplementary file 1 [file DataSheet1.pdf]

SPECIFY PARAMETERS

```
clear
clear global
tic
% Get the current date and time (for file storage)
CurrentDateAndTime = datestr(now,'yyyy-mm-dd HH-MM')
warning('off', 'MATLAB:declareGlobalBeforeUse');
```

Constants

```
% Diffusion Coefficients (m2/s)
%D_Ca_high = 7e-10;
%D_Ca_low = 4e-11;
D_Ca = 3e-10; %(Balyor)
```

```
Kd = 1.1; %(Volpe and Simon, 1991)
```

```
%global D_Ca_high;
%global D_Ca_low;
global D_Ca;
global Kd;
```

Timings

```
% Modelling End Time (s)
% T_End = 0.02; %20ms
T_End = 2; %2s
% Number of Time Points to Sample
T_Resolution = 1000;
% Calculate Sampling Interval
T_Spacing = T_End / T_Resolution;
```

```
global T_Spacing;
global T_End;
global T_Resolution;
```

Geometry

```
% Sarcomere length (m)
l = 3.6e-6;
% Fibre diameter (m)
a = 100e-6;
% Ratio between the SR volume an the whole fibre volume
V_SR_ratio = 0.12;
% Area of total available T membrane
A_TSR = 1.720e-9;
```

```
% diameter of SR element (m)
a_dSR = 3e-8;
% Raduys of SR element (m)
r_dSR = a_dSR/2
```

```
% end-cross sectional area of entire muscle (m^2)
A_mus = pi * a^2 / 4
% half SR length (m)
Half_SR_Length = l/2
% diameter of lumped SR (m)
a_SR = a * sqrt(V_SR_ratio)
% area of lumped SR
A_SR =  pi * a_SR^2 / 4
% SR Radius (m)
SR_Radius =  a_SR/2
% number of SR elements
n = (a_SR/a_dSR)^2
```

```
Mesh_Hmax = 1e-8;
```

```
global l;
global A_TSR;
global a;
global V_SR_ratio;
global a_dSR;
global r_dSR;
```

Definition of Constant Boundary Conditions

```
% only considering Ca2+
% Initial Ca2+ concentration in the SR
u_0 = 3.6;  %mol/m3 1e-3mol/L %(Volpe and Simon, 1991) this is free calcium
% u_total = 8.27;  %(Volpe and Simon, 1991)
u_cyt = 0;
u_Casq = 6.1; %(Volpe and Simon, 1991)
```

```
% flux density of efflux through TST (mol/m^2s)
J_TSR_0_rest = 9.70e-8;
J_TSR_0_high = 3.00e-6;
```

```
% deriving total concentration from u_0 and Kd
u_total = u_0*(u_Casq+u_0+Kd)/(u_0+Kd)
```

Defining the functions for flux calculations:

```
% Total flux leaving SR space
function f = Phi_0_efflux(J_TSR_0)
global A_TSR;
f = - (J_TSR_0 * A_TSR) / 2;
end
```

```
%for constant efflux
Phi_F2_rest = Phi_0_efflux(J_TSR_0_rest)
Phi_F2_high = Phi_0_efflux(J_TSR_0_high)
```

```
J_F2_rest = Phi_F2_rest/A_SR
J_F2_high = Phi_F2_high/A_SR
Phi_F2_high_dSR = J_F2_high * pi * a_dSR^2 / 4
```

```
global J_F2_rest;
global J_F2_high;
global Phi_F2_rest;
global Phi_F2_high;
```

Derive permeability term P_TSR (not used)

```
% calculating P_TSR (not used)
function f = P_TSR(J_TSR_0)
global A_TSR;
global a_SR;
global u_0;
global u_cyt;
f = 2*J_TSR_0*A_TSR/(pi*(a_SR)^2*(u_0-u_cyt));
end
```

Ca efflux at time t (not used)

```
% calculating total efflux at time t (not used)
function f = Phi_t_efflux(~,state,J_TSR_0)
global A_TSR;
global u_0;
global u_cyt;
f = - J_TSR_0*A_TSR/2 * (state.u-u_cyt)/(u_0-u_cyt);
end
```

Ca efflux density at time t (modifed to [Ca2+]_free)

```
% calculating efflux density at time t
function effluxMatrix = J_t_efflux(~, state, J_TSR_0, u_cyt, u_0, a, V_SR_ratio)
global A_TSR;
```

```
% Extract the dependent variable u from the state
u = state.u;
```

```
% Compute the boundary condition
effluxMatrix = -2 * J_TSR_0 * A_TSR * (u - u_cyt) / ((u_0 - u_cyt) * (pi * a^2 * V_SR_ratio));
end
```

```
% calculating efflux density at time t
function effluxMatrix = J_t_efflux_withcasq(~, state, J_TSR_0, u_cyt, u_0, a, V_SR_ratio, u_Casq)
global A_TSR;
global Kd;
```

```
% Extract the dependent variable u from the state
u_total = state.u;
```

```
% Convert to [Ca2+] free
u = (-Kd + u_total - u_Casq + sqrt((Kd - u_total + u_Casq).^2 + 4 .* u_total .* Kd)) / 2;
```

```
% Compute the boundary condition
effluxMatrix = -2 * J_TSR_0 * A_TSR * (u - u_cyt) / ((u_0 - u_cyt) * (pi * a^2 * V_SR_ratio));
end
```

Total replenishing Ca flux (not used)

```
% Replenishing Ca
% total replenishing Ca flux
function f = Phi_t_influx(J)
global A_TSR;
f = -J*A_TSR/2;
end
```

Total replenishing Ca flux into each dSR (not used)

```
% total replenishing Ca flux into each dSR
global A_TSR;
global n;
global l;
global a_dSR;
global V_SR_ratio;
Phi_t_influx_dSR = J_TSR_0_rest*A_TSR*a_dSR^2/(2*a^2*V_SR_ratio)
```

Total replenishing Ca flux into each dSR

```
% replenishing Ca flux density into each dSR
```

```
global A_TSR;
global n;
global l;
global a_dSR;
global V_SR_ratio;
```

```
J_t_influx = J_TSR_0_rest*A_TSR*a_dSR/(pi*a^2*V_SR_ratio*l)
```

```
global J_t_influx
```

Calculating the time for SR to empty with constant flux

```
t_empty_high = pi * a^2 * V_SR_ratio * l * u_0 /(4 * J_TSR_0_high * A_TSR)
t_empty_rest = pi * a^2 * V_SR_ratio * l * u_0 /(4 * J_TSR_0_rest * A_TSR)
```

Adding calsequestrin buffer (modified dissociation cnst)

```
% check that DCa*  is positive for range of [Ca2+] total
state_u = linspace(0, u_total, 1000)
D = D_Ca .* (state_u - (Kd + u_Casq - sqrt((Kd + state_u + u_Casq).^2 - 4 .* state_u .* u_Casq))) ./ (2 .*
state_u);
figure;
plot(state_u, D, 'b', 'LineWidth', 2);
xlabel('state.u');
ylabel('D');
title('Plot of D_{Ca^{2+}}^* as a function of state.u');
grid on;
```

```
% Define Nonlinear Diffusion Coefficient
function cmatrix = nonlinearDiffusionCoeff(location,state,D_Ca, Kd, u_Casq)
    N = 1;
    nr = length(location.x);
    cmatrix = zeros(N,nr);
    try
        cmatrix(1,:) = D_Ca .* (state.u - (Kd + u_Casq - sqrt((Kd + state.u + u_Casq).^2 - 4 .*
        state.u .* u_Casq))) ./ (2 .* state.u);
        catch
        cmatrix(N,nr)=0;
    end
end
```

Geometry and Mesh

```
% Produce a cylindrical geometry with dimensions specified above
```

```
gm = multicylinder(r_dSR,Half_SR_Length);
% Display geometry as assigned to PDE Model
model = createpde(1);
model.Geometry = gm;
```

```
% Produce a model simply for plotting in scale of nm
gm_nm = multicylinder(r_dSR*1e9,Half_SR_Length*1e9);
% Display geometry as assigned to PDE Model
model_nm = createpde(1);
model_nm.Geometry = gm_nm;
```

```
% Figure display options
figure('Position', [100, 100, 800, 600]);  % Set figure size to 800x600
pdegplot(model_nm, 'FaceLabels', 'off');
grid on;
hold on
```

```
% Choose a point on the side of the cylinder
arrowStart = [0, 0];   % Starting point of the arrow
arrowEnd = [r_dSR*1e9, r_dSR*1e9];   % Ending point of the arrow
```

```
% Plot the arrow using quiver3
quiver3(arrowStart(1), arrowStart(2), 0, ...
        arrowEnd(1)*5-arrowStart(1), arrowEnd(2)*5-arrowStart(2), 0, ...
        'MaxHeadSize', 0.5, 'Color', 'black', 'LineWidth', 1);
quiver3(arrowStart(1), arrowStart(2), 1e9*l/4, ...
        arrowEnd(1)*5-arrowStart(1), arrowEnd(2)*5-arrowStart(2), 0, ...
        'MaxHeadSize', 0.5, 'Color', 'black', 'LineWidth', 1);
quiver3(arrowStart(1), arrowStart(2), 1e9*l/2, ...
        arrowEnd(1)*5-arrowStart(1), arrowEnd(2)*5-arrowStart(2), 0, ...
        'MaxHeadSize', 0.5, 'Color', 'black', 'LineWidth', 1);
```

```
% Add a label near the end of the arrow
text(arrowEnd(1)*5, arrowEnd(2)*5, 0, 'F1: Middle of SR element', ...
```

```
'FontSize', 14, 'FontName', 'Arial','Color', 'black', 'HorizontalAlignment', 'left');
text(arrowEnd(1)*5, arrowEnd(2)*5, 1e9*l/4, 'F3: SR membrane', ...
'FontSize', 14, 'FontName', 'Arial','Color', 'black', 'HorizontalAlignment', 'left');
text(arrowEnd(1)*5, arrowEnd(2)*5, 1e9*l/2, 'F2: SR terminal cisternae', ...
'FontSize', 14, 'FontName', 'Arial','Color', 'black', 'HorizontalAlignment', 'left');

title('Geometry of the Half Longitudinal SR element', 'FontSize', 16, 'FontName', 'Arial');
xlabel('Distance (nm)','FontSize', 14, 'FontName', 'Arial');
ylabel('Distance (nm)','FontSize', 14, 'FontName', 'Arial');
zlabel('Distance (nm)','FontSize', 14, 'FontName', 'Arial');
xlim([-r_dSR*6e9,r_dSR*6e9])
ylim([-r_dSR*6e9,r_dSR*6e9])
zlim([0,l*0.5*1e9])

% thicker axis and ticks
ax = findall(gcf, 'Type', 'axes'); % Get all axes in the current figure
set(ax, 'LineWidth', 2); % Apply thick axis lines to all subplots
set(gca, 'FontSize', 14,'FontName', 'Arial');

ticks = [-15,15];
xticks(ticks)
yticks(ticks)

% set view angle
view(45, 30);

hold off
savefig('Geometry.fig');
saveas(gcf,'Geometry.pdf');
saveas(gcf,'Geometry.png');

% Generate a mesh on the geometry with properties described above
meshgeom = generateMesh(model,"Hmax",Mesh_Hmax);
noderet=meshgeom.Nodes;
```

```
figure2 = figure;
pdeplot3D(model,"ElementLabels","off");
hold on
```

```
% Fix the view angle
view([45, 30]);  % Set view angle (azimuth and elevation in degrees)
% Set the axis limits (optional to adjust magnification)
axis([-r_dSR, r_dSR, -r_dSR, r_dSR, l*0.49, l/2]);  % Adjust the axis limits
```

```
title('Finite Element Mesh for Analysis', 'FontSize', 16, 'FontName', 'Arial');
hold off
```

```
savefig('Meshing.fig');
saveas(gcf,'Meshing.pdf');
saveas(gcf,'Meshing.png');
```

Definition of constant Coefficients for counterions

```
specifyCoefficients(model, 'm', 0, 'd', 1, 'c', D_Ca, 'a', 0, 'f', 0);
```

Application of Boundary and Initial Conditions for counterions (Forcing Ca2+ out)

```
% % Apply F1 flux for Ca2+ exit(constant flux)
```

```
% applyBoundaryCondition(model,"neumann","Face",2,"g",J_F2_high,"q",0);
applyBoundaryCondition(model,"neumann","Face",2,"g",J_F2_rest,"q",0);
```

```
% applyBoundaryCondition(model,"neumann","Face",2,"g",@(location, state) J_t_efflux(location, state,
J_TSR_0_rest, u_cyt, u_0, a, V_SR_ratio),"q",0);
```

```
% not flux at closed end and sides
applyBoundaryCondition(model,"neumann","Face",1,"g",0,"q",0);
% applyBoundaryCondition(model,"neumann","Face",3,"g",0,"q",0);
applyBoundaryCondition(model,"neumann","Face",3,"g", J_t_influx,"q",0);
```

```
setInitialConditions(model,u_0);
```

SOLUTION

Run model

```
% % %tlist is the initial time : Sampling Interval : end time in seconds
tlist = 0:T_Spacing:T_End;
% t_inf = 0:T_Spacing:20;
%
% results = solvepde(model,t_inf);
%
% sol = results.NodalSolution;
%
% Sol_time = results.NodalSolution;
% timeForSolution = toc
%
% pdeplot3D(model, 'ColorMapData', results.NodalSolution(:,length(t_inf)));
```

Functions

Interpolating the data points in time and in space along the axis of cylinder

```
% extract solutions at specific points (along the axis
function solValues = extractSolutionAtAxis(results, T_Resolution, num_points)
global l;
tic; % Start timing

% Define the coordinates of the points
xPoints = linspace(0, 0, num_points); % X-coordinates (constant at 0)
yPoints = linspace(0, 0, num_points); % Y-coordinates (constant at 0)
zPoints = linspace(0, l/2, num_points); % Z-coordinates (varying from 0 to 1800 nm)

% Preallocate solution values matrix
solValues = zeros(num_points, T_Resolution + 1);

% Extract solution at the desired points for each time step
for i = 1:T_Resolution + 1
        solValues(:, i) = interpolateSolution(results, xPoints, yPoints, zPoints, i);
end
```

```
% Display elapsed time
fprintf('Time taken to extract results: %.4f seconds\n', toc);
end
```

Interpolating the data points in time and in space along the radius of cylinder

```
% extract solutions at specific points (along the axis
function solValues = extractSolutionAtRadius(results, T_Resolution, num_points, z_coord)
global l;
global r_dSR;
tic; % Start timing

% Define the coordinates of the points
xPoints = linspace(0, r_dSR, num_points); % X-coordinates varrying from (0,r_dSR)
yPoints = linspace(0, 0, num_points); % Y-coordinates (constant at 0)
zPoints = linspace(z_coord, z_coord, num_points); % Z-coordinates at z_coord

% Preallocate solution values matrix
solValues = zeros(num_points, T_Resolution + 1);

% Extract solution at the desired points for each time step
for i = 1:T_Resolution + 1
    solValues(:, i) = interpolateSolution(results, xPoints, yPoints, zPoints, i);
end

% Display elapsed time
fprintf('Time taken to extract results: %.4f seconds\n', toc);
end
```

Defining a function to find half life

```
function T_half = computeHalfLife(solValues, u_0, T_Spacing)
% Number of points
numPoints = size(solValues, 1);

% Preallocate T_half
T_half = zeros(numPoints, 1);
```

```
% Loop over each point to compute the half-life
for i = 1:numPoints
    % Find the index where the solution drops below u_0 / 2
    j = find(solValues(i, :) < u_0 / 2, 1);

    % Calculate half-life
    if isempty(j)
        % If no value drops below u_0 / 2, set half-life to NaN
        T_half(i) = NaN;
    else
        T_half(i) = (j - 1) * T_Spacing;
    end
end
end

%T_half1 = computeHalfLife(solValues1, u_0, T_Spacing);
%T_half2 = computeHalfLife(solValues2, u_0, T_Spacing);
%T_half3 = computeHalfLife(solValues3, u_0, T_Spacing);
```

Defining a function that exports the concentration difference between the two ends of cylinder

```
% function that exports the concentration difference through time
function conc_diff = calculateConcDifference(solValues)

% Determine indices for the required positions
idx_bottom = 1;                % Position at (0,0,0)
idx_top = size(solValues, 1);  % Position at (0,0,height)

% Calculate concentration difference for each time point
conc_diff = solValues(idx_bottom, :) - solValues(idx_top, :);

end
```

```
function plotConcDifferenceSamePlot(solValuesArray, tlist, plot_titles, figure_name)

% Create a new figure
figure;
hold on;

% Loop through each solValues dataset and plot on the same graph
for i = 1:length(solValuesArray)
    % Get the corresponding solValues
    solValues = solValuesArray{i};

    % Determine indices for the required positions
    idx_bottom = 1;             % Position at (0,0,0)
    idx_top = size(solValues, 1);  % Position at (0,0,height)

    % Calculate concentration difference for each time point
    conc_diff = solValues(idx_bottom, :) - solValues(idx_top, :);



    % Plot the concentration difference over time
    plot(tlist, conc_diff, 'LineWidth', 1.5);
end

% Add labels, title, and legend
xlabel('Time (s)','FontSize', 14, 'FontName', 'Arial');
ylabel('Concentration Difference (mol/m^3)','FontSize', 16, 'FontName', 'Arial');
title(figure_name, 'Interpreter', 'tex', 'FontSize', 16, 'FontName', 'Arial');
legend(plot_titles, 'Location', 'Best');
grid on;

% thicker axis and ticks
ax = findall(gcf, 'Type', 'axes'); % Get all axes in the current figure
```

```
set(ax, 'LineWidth', 2); % Apply thick axis lines to all subplots
set(gca, 'FontSize', 14,'FontName', 'Arial');

hold off;
```

```
% Adjust the layout to prevent overlap
set(gcf, 'Position', [100, 100, 720, 480]); % Adjust figure size for better visibility
```

```
% Generate filenames based on figure_name
safe_name = regexprep(figure_name, '[^\w]', '_'); % Replace non-word characters with underscores
png_filename = sprintf('%s.png', safe_name);
pdf_filename = sprintf('%s.pdf', safe_name);
fig_filename = sprintf('%s.fig', safe_name);
```

```
% Save the figure as PNG and PDF
exportgraphics(gcf, png_filename, 'Resolution', 300); % Save as PNG with high resolution
exportgraphics(gcf, pdf_filename, 'ContentType', 'vector'); % Save as a vectorized PDF
savefig(fig_filename);
end
```

Exporting the total flux out of lumped SR at release site

```
function efflux = compute_efflux(results, T_Resolution, u_0, J_TSR_0, A_TSR, u_cyt)
```

```
% Initialize efflux array
efflux = zeros(1, T_Resolution + 1);
```

```
% Compute efflux for each time step
for i = 1:T_Resolution + 1
    u_i = interpolateSolution(results, 0, 0, 0, i);
    efflux(i) = J_TSR_0 * A_TSR * (u_i - u_cyt) / (2 * (u_0 - u_cyt));
end
end
```

Plot the solution over time of the whole cylinder (not so good)

```
% % ploting the behaviour of [Ca2+] over t
% num_plots = 10;
% selected_indices = round(linspace(1, length(tlist), num_plots));
%
% % Create a figure with subplots for each selected time step
% figure3 = figure;
% for i = 1:num_plots
%     k = selected_indices(i); % Get the index of the selected time step
%
%     % Create a subplot for the current time step
%     subplot(2, num_plots/2, i); % Arrange subplots in a 2x3 grid (adjust as needed)
%
%     % Plot the solution at the current time step
%     pdeplot3D(model, 'ColorMapData', results.NodalSolution(:, k));
%     colorbar;
%     title(['Time = ', num2str(tlist(k)),'s']);
%     xlabel('X');
%     ylabel('Y');
%     zlabel('Z');
%
%     % Set consistent color scale across subplots
%     caxis([min(results.NodalSolution(:)) max(results.NodalSolution(:))]);
%     colorbar_handle = colorbar;
%     colorbar_handle.Label.String = 'Concentration (mol/m^3)';
%
% end
%
% % Add a main title to the figure
% t = '3D Solution at Selected Time Steps With D_{Ca} =3\times10^{-10}m^2/s';
% sgtitle(t,'FontSize', 20)
```

```
% define a plotting function that plots at constant intervals (not so good)
function plotCalciumBehavior(tlist, results, plot_title, model)
% Define the number of plots
num_plots = 10;
selected_indices = round(linspace(1, length(tlist), num_plots));
```

```
% Create a larger figure
figure;
set(gcf, 'Position', [100, 100, 1400, 600]); % Adjust figure size
tiledlayout(2, num_plots/2, 'Padding', 'compact', 'TileSpacing', 'compact');

% Set a consistent color scale
clim = [min(results.NodalSolution(:)) max(results.NodalSolution(:))];

for i = 1:num_plots
    k = selected_indices(i); % Get the index of the selected time step

    % Create a subplot for the current time step
    nexttile;

    % Plot the solution at the current time step
    pdeplot3D(model, 'ColorMapData', results.NodalSolution(:, k));
    title(['Time = ', num2str(tlist(k)), ' s']);
    xlabel('X');
    ylabel('Y');
    zlabel('Z');
    caxis(clim); % Apply consistent color limits
end

% Add a main title to the figure
sgtitle(plot_title,'FontSize', 12);

% Generate filenames based on title_name
safe_title = regexprep(plot_title, '[^\w]', '_'); % Replace non-word characters with underscores
png_filename = sprintf('%s.png', safe_title);
pdf_filename = sprintf('%s.pdf', safe_title);
fig_filename = sprintf('%s.fig', safe_title);

% Save the figure
```

```
exportgraphics(gcf, png_filename, 'Resolution', 300); % Save as PNG with high resolution
exportgraphics(gcf, pdf_filename, 'ContentType', 'vector'); % Save as a vectorized PDF
savefig(fig_filename);

end


% Define the specific time points to plot (not so good)

selected_times = [6.25e-4, 0.00125, 0.0025, 0.005, 0.01, 0.02];

% define a plotter function at specific times
function plotPDEResults(selected_times, tlist, results, plot_title, model)
% Find the closest indices in tlist for the selected times
selected_indices = arrayfun(@(t) find(abs(tlist - t) == min(abs(tlist - t)), 1), selected_times);

% Create a larger figure
figure;
set(gcf, 'Position', [100, 100, 1400, 600]); % Adjust figure size
tiledlayout(1, numel(selected_times), 'Padding', 'compact', 'TileSpacing', 'compact');

% Set a consistent color scale
clim = [min(results.NodalSolution(:)) max(results.NodalSolution(:))];
for i = 1:numel(selected_times)
    k = selected_indices(i); % Get the index of the selected time step

    % Create a subplot for the current time step
    nexttile;

    % Plot the solution at the current time step
    pdeplot3D(model, 'ColorMapData', results.NodalSolution(:, k));
    title(['Time = ', num2str(selected_times(i)), ' s']);
    xlabel('X');
    ylabel('Y');
```

```
        zlabel('Z');
        caxis(clim); % Apply consistent color limits
    end
```

```
    % Add a main title to the figure
    sgtitle(plot_title,'FontSize', 12);
```

```
    % Generate filenames based on title_name
    safe_title = regexprep(plot_title, '[^\w]', '_'); % Replace non-word characters with underscores
    png_filename = sprintf('%s.png', safe_title);
    pdf_filename = sprintf('%s.pdf', safe_title);
    fig_filename = sprintf('%s.fig', safe_title);
```

```
    % Save the figure
    exportgraphics(gcf, png_filename, 'Resolution', 300); % Save as PNG with high resolution
    exportgraphics(gcf, pdf_filename, 'ContentType', 'vector'); % Save as a vectorized PDF
    savefig(fig_filename);
    end
```

Plot along the z-axis of SR over time

```
    % Visualize the solution in over time
    function visualizeCt(tlist, num_points, solValues, plot_title)
```

```
    zPoints = linspace(0, 1800e-9, num_points);
```

```
    figure; % Create a new figure
    imagesc(tlist, zPoints, solValues); % Use imagesc for a 2D heatmap

    % Customize the color map and color bar
    colormap(jet); % Choose a colormap (e.g., jet, hot, parula)
    colorbar; % Add a color bar to indicate concentration values
```

```
% Add labels and title
xlabel('Time (s)');
ylabel('Location (m)');
title(plot_title, 'Interpreter', 'tex','FontSize', 12); % Use TeX interpreter for formatting
```

```
% Improve visualization
set(gca, 'YDir', 'normal'); % Ensure the Y-axis direction is correct
```

```
% Generate filenames based on title_name
safe_title = regexprep(plot_title, '[^\w]', '_'); % Replace non-word characters with underscores
png_filename = sprintf('%s.png', safe_title);
pdf_filename = sprintf('%s.pdf', safe_title);
fig_filename = sprintf('%s.fig', safe_title);
```

```
% Save the figure
exportgraphics(gcf, png_filename, 'Resolution', 300); % Save as PNG with high resolution
exportgraphics(gcf, pdf_filename, 'ContentType', 'vector'); % Save as a vectorized PDF
savefig(fig_filename);
end
```

Creating a collection of color plots for different conditions

```
function visualizeMultipleCt_axis(tlist, num_points, solValuesArray, plot_titles, figure_name)
global l;
```

```
% Determine the number of solutions
n = length(solValuesArray);
```

```
% Calculate subplot grid size
rows = ceil(sqrt(n));
cols = ceil(n / rows);
```

```
% Find the global minimum and maximum values across all solutions
allValues = cell2mat(cellfun(@(x) x(:), solValuesArray, 'UniformOutput', false));
clim_min = min(allValues)
clim_max = max(allValues)

% Create a new figure
figure;

% Loop through each solution and create a subplot
for i = 1:n
    % Select the subplot position
    subplot(rows, cols, i);

    % Get the corresponding solValues and title for this subplot
    solValues = solValuesArray{i};
    plot_title = plot_titles{i};

    % Define zPoints for the current solution
    zPoints = linspace(0, l/2, num_points);

    % Plot the current solution
    imagesc(tlist, zPoints, solValues);

    % Set consistent color limits for all subplots
    caxis([min(clim_min) max(clim_max)]);

    % Customize the color map and color bar
    colormap(jet);
    cb = colorbar();
    ylabel(cb,'Concentration (mol/m^3)','FontSize',14,'Rotation',90)

    % Add labels and title for this subplot
    xlabel('Time (s)','FontSize', 14, 'FontName', 'Arial');
```

```
        ylabel('Location on Axis (m)', 'FontSize', 14, 'FontName', 'Arial');
        title(plot_title, 'Interpreter', 'tex', 'FontSize', 16,'FontName', 'Arial');

        % Improve visualization
        set(gca, 'YDir', 'normal');
    end

    % Add an overall title to the figure
    sgtitle(figure_name, 'FontSize', 20, 'FontName', 'Arial');

    % Adjust the layout to prevent overlap
    set(gcf, 'Position', [100, 100, 1080, 720]); % Adjust figure size for better visibility

    % thicker axis and ticks
    ax = findall(gcf, 'Type', 'axes'); % Get all axes in the current figure
    set(ax, 'LineWidth', 2); % Apply thick axis lines to all subplots
    set(gca, 'FontSize', 14,'FontName', 'Arial');

    % Generate filenames based on figure_name
    safe_name = regexprep(figure_name, '[^\w]', '_'); % Replace non-word characters with underscores
    png_filename = sprintf('%s.png', safe_name);
    pdf_filename = sprintf('%s.pdf', safe_name);
    fig_filename = sprintf('%s.fig', safe_name);

    % Save the figure as PNG and PDF
    exportgraphics(gcf, png_filename, 'Resolution', 300); % Save as PNG with high resolution
    exportgraphics(gcf, pdf_filename, 'ContentType', 'vector'); % Save as a vectorized PDF
    savefig(fig_filename);
    end


    function visualizeMultipleCt_radius(tlist, num_points, solValuesArray, plot_titles, figure_name)
    global l;
    global r_dSR;
```

```
global u_0;

% Determine the number of solutions
n = length(solValuesArray);

% Calculate subplot grid size
rows = ceil(sqrt(n));
cols = ceil(n / rows);

% Find the global minimum and maximum values across all solutions
allValues = cell2mat(cellfun(@(x) x(:), solValuesArray, 'UniformOutput', false));
clim_min = min(allValues)
clim_max = max(allValues)

% Create a new figure
figure;

% Loop through each solution and create a subplot
for i = 1:n
    % Select the subplot position
    subplot(rows, cols, i);

    % Get the corresponding solValues and title for this subplot
    solValues = solValuesArray{i};
    plot_title = plot_titles{i};

    % Define zPoints for the current solution
    xPoints = linspace(0, r_dSR, num_points);

    % Plot the current solution
    imagesc(tlist, xPoints, solValues);

    % Set consistent color limits for all subplots
```

```
            caxis([min(clim_min) max(clim_max)]);

            % Customize the color map and color bar
            colormap(jet);
            cb = colorbar();
            ylabel(cb,'Concentration (mol/m^3)','FontSize',14,'Rotation',90)

            % Add labels and title for this subplot
            xlabel('Time (s)','FontSize', 14, 'FontName', 'Arial');
            ylabel('Location on Radius (m)', 'FontSize', 14, 'FontName', 'Arial');
            title(plot_title, 'Interpreter', 'tex', 'FontSize', 16,'FontName', 'Arial');

            % Improve visualization
            set(gca, 'YDir', 'normal');
    end

    % Add an overall title to the figure
    sgtitle(figure_name, 'FontSize', 20, 'FontName', 'Arial');

    % Adjust the layout to prevent overlap
    set(gcf, 'Position', [100, 100, 1080, 720]); % Adjust figure size for better visibility

    % thicker axis and ticks
    ax = findall(gcf, 'Type', 'axes'); % Get all axes in the current figure
    set(ax, 'LineWidth', 2); % Apply thick axis lines to all subplots
    set(gca, 'FontSize', 14,'FontName', 'Arial');

    % Generate filenames based on figure_name
    safe_name = regexprep(figure_name, '[^\w]', '_'); % Replace non-word characters with underscores
    png_filename = sprintf('%s.png', safe_name);
    pdf_filename = sprintf('%s.pdf', safe_name);
    fig_filename = sprintf('%s.fig', safe_name);
```

```
% Save the figure as PNG and PDF
exportgraphics(gcf, png_filename, 'Resolution', 300); % Save as PNG with high resolution
exportgraphics(gcf, pdf_filename, 'ContentType', 'vector'); % Save as a vectorized PDF
savefig(fig_filename);
end
```

Visualize the solution at N positions

```
function plotNLines(solValues, tlist, u_0, plot_title, N)

figure; % Create a new figure

% Determine indices for N evenly spaced points
num_points = size(solValues, 1);
indices = round(linspace(1, num_points, N));

% Plot the concentration at the selected positions
hold on;
lines = gobjects(1, N); % Preallocate for line handles
colors = cool(N);

for i = 1:N
    lines(i) = plot(tlist, solValues(indices(i), :), 'LineWidth', 1.5, ...
                    'Color', colors(i, :));
end

position_names = arrayfun(@(x) sprintf('Position %d', N+1-x), 1:N, 'UniformOutput', false);

% Add labels and title
xlabel('Time (s)','FontSize', 14, 'FontName', 'Arial');
ylabel('Concentration (mol/m^3)','FontSize', 14, 'FontName', 'Arial');
title(plot_title, 'Interpreter', 'tex', 'FontSize', 16,'FontName', 'Arial');

% Add a legend
```

```
legend(flip(lines), flip(position_names), 'Location', 'Best');

% Add grid
grid on;

hold off;

% Generate filenames based on title_name
safe_title = regexprep(plot_title, '[^\w]', '_'); % Replace non-word characters with underscores
png_filename = sprintf('%s.png', safe_title);
pdf_filename = sprintf('%s.pdf', safe_title);
fig_filename = sprintf('%s.fig', safe_title);

% Save the figure
exportgraphics(gcf, png_filename, 'Resolution', 300); % Save as PNG with high resolution
exportgraphics(gcf, pdf_filename, 'ContentType', 'vector'); % Save as a vectorized PDF
savefig(fig_filename);
end


% plot all six in one
function plotMultipleNLines(solValuesArray, tlist, u_0, plot_titles, N, figure_name)
% Determine the number of solutions
n = length(solValuesArray);

% Calculate subplot grid size
rows = ceil(sqrt(n));
cols = ceil(n / rows);

% Find the global minimum and maximum values across all solutions
allValues = cell2mat(cellfun(@(x) x(:), solValuesArray, 'UniformOutput', false));
clim_min = min(allValues);

% Create a new figure
figure;
```

```
% Loop through each solution and create a subplot
for i = 1:n
    % Select the subplot position
    subplot(rows, cols, i);

    % Get the corresponding solValues and title for this subplot
    solValues = solValuesArray{i};
    plot_title = plot_titles{i};

    % Determine indices for N evenly spaced points
    num_points = size(solValues, 1);
    indices = round(linspace(1, num_points, N));

    % Plot the concentration at the selected positions
    hold on;
    lines = gobjects(1, N); % Preallocate for line handles
    colors = cool(N);

    for i = 1:N
        lines(i) = plot(tlist, solValues(indices(i), :), 'LineWidth', 1.5, ...
                      'Color', colors(i, :));
    end

    position_names = arrayfun(@(x) sprintf('Position %d', N+1-x), 1:N, 'UniformOutput', false);

    % Add labels and title
    xlabel('Time (s)','FontSize', 14, 'FontName', 'Arial');
    ylabel('Concentration (mol/m^3)','FontSize', 14, 'FontName', 'Arial');
    title(plot_title, 'Interpreter', 'tex', 'FontSize', 16,'FontName', 'Arial');
    ylim([min(clim_min),u_0])

    % Add a legend
```

```
        legend(flip(lines), flip(position_names), 'Location', 'Best','FontSize', 14,'FontName', 'Arial');

        % Add grid
        hold off;
    end

    % Add an overall title to the figure
    sgtitle(figure_name, 'FontSize', 20, 'FontName', 'Arial');

    % Adjust the layout to prevent overlap
    set(gcf, 'Position', [100, 100, 1080, 720]); % Adjust figure size for better visibility

    % thicker axis and ticks
    ax = findall(gcf, 'Type', 'axes'); % Get all axes in the current figure
    set(ax, 'LineWidth', 2); % Apply thick axis lines to all subplots
    set(gca, 'FontSize', 14,'FontName', 'Arial');

    % Generate filenames based on figure_name
    safe_name = regexprep(figure_name, '[^\w]', '_'); % Replace non-word characters with underscores
    png_filename = sprintf('%s.png', safe_name);
    pdf_filename = sprintf('%s.pdf', safe_name);
    fig_filename = sprintf('%s.fig', safe_name);

    % Save the figure as PNG and PDF
    exportgraphics(gcf, png_filename, 'Resolution', 300); % Save as PNG with high resolution
    exportgraphics(gcf, pdf_filename, 'ContentType', 'vector'); % Save as a vectorized PDF
    savefig(fig_filename);
    end


    function plotConcentrationProfiles(solValues, N, figure_name)
    global T_Spacing;
    % Determine the number of time points available
    num_time_points = size(solValues, 2);
```

```
% Calculate indices for N equally spaced time points
time_indices = round(linspace(1, num_time_points, N));
```

```
% Create the position array (assuming evenly spaced points along the height)
num_points = size(solValues, 1);
zPoints = linspace(0, 1800e-9, num_points); % Example height range
```

```
% Create a new figure
figure;
hold on;
```

```
% Generate a colormap for consistent coloring
colors = parula(N);
```

```
% Plot the concentration profile for each selected time point
for i = 1:N
    idx = time_indices(i);
    plot(zPoints, solValues(:, idx), 'LineWidth', 1.5, 'Color', colors(i, :));
end
```

```
% Add labels and legend
xlabel('Position (m)');
ylabel('Concentration (mol/m^3)');
title('Concentration Profiles at Selected Time Points');
legend_labels = arrayfun(@(x) sprintf('t = %.4f s', round((x-1)*T_Spacing, 4)), time_indices, 'UniformOutput',
false);
legend(legend_labels, 'Location', 'Best');
grid on;
hold off;
```

```
% Adjust the layout for better visibility
set(gcf, 'Position', [100, 100, 1200, 800]); % Adjust figure size
```

```
% Generate filenames for saving
safe_name = regexprep(figure_name, '[^\w]', '_'); % Replace non-word characters with underscores
png_filename = sprintf('%s.png', safe_name);
pdf_filename = sprintf('%s.pdf', safe_name);
fig_filename = sprintf('%s.fig', safe_name);

% Save the figure as PNG and PDF
exportgraphics(gcf, png_filename, 'Resolution', 300); % Save as PNG with high resolution
exportgraphics(gcf, pdf_filename, 'ContentType', 'vector'); % Save as a vectorized PDF
savefig(fig_filename);
end


% plot all six in one
function plotMultipleConcentrationProfiles(solValuesArray, u_0, plot_titles, N, figure_name)
global T_Spacing;
global l;

% Determine the number of solutions
n = length(solValuesArray);

% Calculate subplot grid size
rows = ceil(sqrt(n));
cols = ceil(n / rows);

% Find the global minimum and maximum values across all solutions
allValues = cell2mat(cellfun(@(x) x(:), solValuesArray, 'UniformOutput', false));
clim_min = min(allValues);

% Create a new figure
figure;

% Loop through each solution and create a subplot
for i = 1:n
```

```
% Select the subplot position
subplot(rows, cols, i);

% Get the corresponding solValues and title for this subplot
solValues = solValuesArray{i};
plot_title = plot_titles{i};

% Determine the number of time points available
num_time_points = size(solValues, 2);

% Calculate indices for N equally spaced time points
time_indices = round(linspace(1, num_time_points, N));

% Create the position array (assuming evenly spaced points along the height)
num_points = size(solValues, 1);
zPoints = linspace(0, 1800e-9, num_points); % Example height range

% Plot the concentration at the selected positions
hold on;

% Generate a colormap for consistent coloring
colors = parula(N);

% Plot the concentration profile for each selected time point
for i = 1:N
    idx = time_indices(i);
    plot(zPoints, solValues(:, idx), 'LineWidth', 1.5, 'Color', colors(i, :));
end

% Add labels and legend
xlabel('Position (m)','FontSize', 14, 'FontName', 'Arial');
ylabel('Concentration (mol/m^3)','FontSize', 14, 'FontName', 'Arial');
title(plot_title, 'Interpreter', 'tex', 'FontSize', 16,'FontName', 'Arial');
```

```
        xlim([0,l/2])
        ylim([min(clim_min),u_0])

        % Add a legend
        legend_labels = arrayfun(@(x) sprintf('t = %.4f s', round((x-1)*T_Spacing, 4)), time_indices,
        'UniformOutput', false);
        legend(legend_labels, 'Location', 'southwest','FontSize', 14,'FontName', 'Arial');

        % Add grid
        grid on;
        hold off;

    end

    % Add an overall title to the figure
    sgtitle(figure_name, 'FontSize', 20, 'FontName', 'Arial');

    % Adjust the layout to prevent overlap
    set(gcf, 'Position', [100, 100, 1080, 720]); % Adjust figure size for better visibility

    % thicker axis and ticks
    ax = findall(gcf, 'Type', 'axes'); % Get all axes in the current figure
    set(ax, 'LineWidth', 2); % Apply thick axis lines to all subplots
    set(gca, 'FontSize', 14,'FontName', 'Arial');

    % Generate filenames based on figure_name
    safe_name = regexprep(figure_name, '[^\w]', '_'); % Replace non-word characters with underscores
    png_filename = sprintf('%s.png', safe_name);
    pdf_filename = sprintf('%s.pdf', safe_name);
    fig_filename = sprintf('%s.fig', safe_name);

    % Save the figure as PNG and PDF
    exportgraphics(gcf, png_filename, 'Resolution', 300); % Save as PNG with high resolution
```

```
exportgraphics(gcf, pdf_filename, 'ContentType', 'vector'); % Save as a vectorized PDF
savefig(fig_filename);
end
```

Plotting graphs for indicating interpolated points on the cylinder

```
% Parameters for the cylinder
theta = linspace(0, pi, 100);  % Half cylinder (0 to pi)
z = linspace(0, 1e9 * l/2, 100);
[Theta, Z] = meshgrid(theta, z);
X = r_dSR * cos(Theta) * 1e9;
Y = r_dSR * sin(Theta) * 1e9;
```

```
% Plot the half-cylinder
figure;
surf(X, Y, Z, 'FaceColor', [0.7, 0.7, 0.7], 'EdgeColor', 'none', 'FaceAlpha', 0.5);
hold on;
```

```
% Plot the circular base and top
fill3(r_dSR * cos(theta) * 1e9, r_dSR * sin(theta) * 1e9, zeros(size(theta)), [0.7, 0.7, 0.7], 'FaceAlpha', 0.5);
fill3(r_dSR * cos(theta) * 1e9, r_dSR * sin(theta) * 1e9, 1e9 * l/2 * ones(size(theta)), [0.7, 0.7, 0.7],
'FaceAlpha', 0.5);
```

```
% Plot the axis along the Z direction
plot3([0, 0], [0, 0], [0, 1e9 * l/2], 'r-', 'LineWidth', 3);
```

```
% Label the axis
text(0, 0, 1e9 * l/2, 'Axis', 'FontSize', 14,'FontName', 'Arial', 'Color', 'red', ...
'HorizontalAlignment', 'right', 'VerticalAlignment', 'bottom', ...
'FontWeight', 'bold');
```

```
% Adjust the aspect ratio
```

```
ax = gca; % Get the current axes handle
ax.DataAspectRatio = [1, 1, 12]; % Make Z axis units twice as long as X and Y
```

```
% Enhance the view
grid on;
title('Positions of Extracted Axial Solutions','FontSize', 16,'FontName', 'Arial');
xlabel('X(nm)','FontSize', 14,'FontName', 'Arial');
ylabel('Y(nm)','FontSize', 14,'FontName', 'Arial');
zlabel('Z(nm)','FontSize', 14,'FontName', 'Arial');
zlim([0,l*0.5*1e9])
view([-30, 30]);  % View angle for better visualization
```

```
% thicker axis and ticks
ax = findall(gcf, 'Type', 'axes'); % Get all axes in the current figure
set(ax, 'LineWidth', 2); % Apply thick axis lines to all subplots
set(gca, 'FontSize', 14,'FontName', 'Arial');
```

```
xticks([-15,0,15])
yticks([0,15])
```

```
hold off;
savefig('Axial_slice.fig');
saveas(gcf,'Axial_slice.pdf');
saveas(gcf,'Axial_slice.png');
```

```
% Parameters for the cylinder
theta = linspace(0, 2*pi, 100);  % Half cylinder (0 to pi)
z = linspace(0, 1e9 * l/2, 100);
[Theta, Z] = meshgrid(theta, z);
X = r_dSR * cos(Theta) * 1e9;
Y = r_dSR * sin(Theta) * 1e9;
```

```
% Plot the half-cylinder
figure;
```

```
surf(X, Y, Z, 'FaceColor', [0.7, 0.7, 0.7], 'EdgeColor', 'none', 'FaceAlpha', 0.5);
hold on;
```

```
% Plot the circular base and top
fill3(r_dSR * cos(theta) * 1e9, r_dSR * sin(theta) * 1e9, zeros(size(theta)), [0.7, 0.7, 0.7], 'FaceAlpha', 0.5);
fill3(r_dSR * cos(theta) * 1e9, r_dSR * sin(theta) * 1e9, 1e9 * l/2 * ones(size(theta)), [0.7, 0.7, 0.7],
'FaceAlpha', 0.5);
```

```
% Plot the radius along the positive X-axis
plot3([0, 0], [-1e9 * r_dSR, 0], [1e9 * l/2 , 1e9 * l/2], 'r-', 'LineWidth', 3);  % Radius in red
plot3([0, 0], [-1e9 * r_dSR, 0], [0 , 0], 'r-', 'LineWidth', 3);  % Radius in red
```

```
% Label the radius
text(1e9 * r_dSR * 4, -1e9 * r_dSR * 2, 1e9 * l * 0.50, 'Radius at Terminal Cisternae', ...
'FontSize', 14, 'FontName', 'Arial', 'Color', 'red', ...
'HorizontalAlignment', 'center', ...
'VerticalAlignment', 'bottom', ...
'FontWeight', 'bold');
text(1e9 * r_dSR * 4, -1e9 * r_dSR * 2, 0, 'Radius at SR Centre', ...
'FontSize', 14, 'FontName', 'Arial', 'Color', 'red', ...
'HorizontalAlignment', 'center', ...
'VerticalAlignment', 'bottom', ...
'FontWeight', 'bold');
```

```
% Adjust the aspect ratio
ax = gca; % Get the current axes handle
ax.DataAspectRatio = [1, 1, 12]; % Make Z axis units twice as long as X and Y
```

```
% Enhance the view
grid on;
title('Positions of Extracted Axial Solutions','FontSize', 16,'FontName', 'Arial');
xlabel('X(nm)','FontSize', 14,'FontName', 'Arial');
ylabel('Y(nm)','FontSize', 14,'FontName', 'Arial');
zlabel('Z(nm)','FontSize', 14,'FontName', 'Arial');
```

```
zlim([0,l*0.5*1e9])
view([-30, 30]);  % View angle for better visualization
```

```
% thicker axis and ticks
ax = findall(gcf, 'Type', 'axes'); % Get all axes in the current figure
set(ax, 'LineWidth', 2); % Apply thick axis lines to all subplots
set(gca, 'FontSize', 14,'FontName', 'Arial');
```

```
xticks([-15,0,15])
yticks([0,15])
```

```
hold off;
savefig('Radial_slice.fig');
saveas(gcf,'Radial_slice.pdf');
saveas(gcf,'Radial_slice.png');
```

```
% Parameters for the cylinder
theta = linspace(0, pi, 100);  % Half cylinder (0 to pi)
z = linspace(0, 1e9 * l/2, 100);
[Theta, Z] = meshgrid(theta, z);
X = r_dSR * cos(Theta) * 1e9;
Y = r_dSR * sin(Theta) * 1e9;
```

```
% Plot the half-cylinder
figure;
surf(X, Y, Z, 'FaceColor', [0.7, 0.7, 0.7], 'EdgeColor', 'none', 'FaceAlpha', 0.5);
hold on;
```

```
% Plot the circular base and top
fill3(r_dSR * cos(theta) * 1e9, r_dSR * sin(theta) * 1e9, zeros(size(theta)), [0.7, 0.7, 0.7], 'FaceAlpha', 0.5);
fill3(r_dSR * cos(theta) * 1e9, r_dSR * sin(theta) * 1e9, 1e9 * l/2 * ones(size(theta)), [0.7, 0.7, 0.7],
'FaceAlpha', 0.5);
```

```
% Plot the axis along the Z direction
plot3([0, 0], [0, 0], [0, 1e9 * l/2], 'b-', 'LineWidth', 1);

% Select 5 evenly spaced points along the axis
n_points = 5;
z_points = linspace(0, 1e9 * l/2, n_points);
x_points = zeros(1, n_points);
y_points = zeros(1, n_points);

% Get colors from the 'cool' colormap
cmap = cool(n_points);

% Plot the points with colors
scatter3(x_points, y_points, z_points, 100, cmap, 'filled'); % 100 sets marker size

% Add labels to each point
for i = 1:n_points
text(x_points(i), y_points(i), z_points(i), sprintf('Position %d', 6-i), ...
        'FontSize', 12, 'FontWeight', 'bold', 'FontName', 'Arial', 'Color', 'k', ...
        'HorizontalAlignment', 'left', 'VerticalAlignment', 'bottom');
end

% Adjust the aspect ratio
ax = gca; % Get the current axes handle
ax.DataAspectRatio = [1, 1, 12]; % Make Z axis units twice as long as X and Y

% Enhance the view
grid on;
title('Positions of Extracted Axial Solutions', 'FontSize', 16, 'FontName', 'Arial');
xlabel('X (nm)', 'FontSize', 14, 'FontName', 'Arial');
ylabel('Y (nm)', 'FontSize', 14, 'FontName', 'Arial');
zlabel('Z (nm)', 'FontSize', 14, 'FontName', 'Arial');
zlim([0, l * 0.5 * 1e9])
view([-30, 30]);  % View angle for better visualization
```

```
% Thicker axis and ticks
ax = findall(gcf, 'Type', 'axes'); % Get all axes in the current figure
set(ax, 'LineWidth', 2); % Apply thick axis lines to all subplots
set(gca, 'FontSize', 14, 'FontName', 'Arial');
```

```
xticks([-15, 0, 15])
yticks([0, 15])
```

```
hold off;
```

```
savefig('Axial_slice_5_positions.fig');
saveas(gcf,'Axial_slice_5_positions.pdf');
saveas(gcf,'Axial_slice_5_positions.png');
```

Finding the time for [Ca++] to reach 0 or half life at each point

```
% i_1 = find(solValues(1, :) < 0, 1);
% T_empty_1 = (i_1-1) * T_Spacing;
%
% i_2 = find(solValues(2, :) < 0, 1);
% T_empty_2 = (i_2-1) * T_Spacing;
%
% i_3 = find(solValues(3, :) < 0, 1);
% T_empty_3 = (i_3-1) * T_Spacing;
%
% j_1 = find(solValues(1, :) < u_0/2, 1);
% T_half_1 = (j_1-1) * T_Spacing;
%
% j_2 = find(solValues(2, :) < u_0/2, 1);
% T_half_2 = (j_2-1) * T_Spacing;
%
% j_3 = find(solValues(3, :) < u_0/2, 1);
% T_half_3 = (j_3-1) * T_Spacing;
```

Running the program in terms of [Ca2+] free

```
tic
% model with constant Ca2+ efflux and no other special conditions
model1f = createpde(1);
model1f.Geometry = gm;
meshgeom1f = generateMesh(model1f,"Hmax",Mesh_Hmax);
```

```
specifyCoefficients(model1f, 'm', 0, 'd', 1, 'c', D_Ca, 'a', 0, 'f', 0);
```

```
% % Apply F1 flux for Ca2+ exit(constant flux)
applyBoundaryCondition(model1f,"neumann","Face",2,"g",J_F2_high,"q",0);
```

```
% not flux at closed end and sides
applyBoundaryCondition(model1f,"neumann","Face",1,"g",0,"q",0);
applyBoundaryCondition(model1f,"neumann","Face",3,"g",0,"q",0);
```

```
setInitialConditions(model1f,u_0);
```

```
results1f = solvepde(model1f,tlist)
```

```
sol1f = results1f.NodalSolution;
```

```
Sol_time1f = results1f.NodalSolution;
timeForSolution1f = toc
```

```
tic
% model with non-constant Ca2+ efflux without influx without calsequetrin
model3f = createpde(1);
model3f.Geometry = gm;
meshgeom3f = generateMesh(model3f,"Hmax",Mesh_Hmax);
```

```
specifyCoefficients(model3f, 'm', 0, 'd', 1, 'c', D_Ca, 'a', 0, 'f', 0);
```

```
applyBoundaryCondition(model3f,"neumann","Face",2,"g",@(location, state) J_t_efflux(location, state,
J_TSR_0_high, u_cyt, u_0, a, V_SR_ratio),"q",0);

% not flux at closed end and sides
applyBoundaryCondition(model3f,"neumann","Face",1,"g",0,"q",0);
applyBoundaryCondition(model3f,"neumann","Face",3,"g",0,"q",0);

setInitialConditions(model3f,u_0);

results3f = solvepde(model3f,tlist)

sol3f = results3f.NodalSolution;

Sol_time3f = results3f.NodalSolution;
timeForSolution3f = toc

% apply the new DCa to the model
tic
% model with non-constant Ca2+ efflux with influx without calsequestrin
model4f = createpde(1);
model4f.Geometry = gm;
meshgeom4f = generateMesh(model4f,"Hmax",Mesh_Hmax);

specifyCoefficients(model4f, 'm', 0, 'd', 1, 'c', D_Ca, 'a', 0, 'f', 0);

applyBoundaryCondition(model4f,"neumann","Face",2,"g",@(location, state) J_t_efflux(location, state,
J_TSR_0_high, u_cyt, u_0, a, V_SR_ratio),"q",0);

% not flux at closed end and sides
applyBoundaryCondition(model4f,"neumann","Face",1,"g",0,"q",0);
```

```
applyBoundaryCondition(model4f,"neumann","Face",3,"g", J_t_influx,"q",0);
```

```
setInitialConditions(model4f,u_0);
```

```
results4f = solvepde(model4f,tlist)
```

```
sol4f = results4f.NodalSolution;
```

```
Sol_time4f = results4f.NodalSolution;
timeForSolution4f = toc
%T_half4 = computeHalfLife(solValues4, u_0, T_Spacing);
```

Run model at multiple boundary conditions and plot them together (Six cases in total)

```
tic
% model with constant Ca2+ efflux and no other special conditions
model1 = createpde(1);
model1.Geometry = gm;
meshgeom1 = generateMesh(model1,"Hmax",Mesh_Hmax);
```

```
specifyCoefficients(model1, 'm', 0, 'd', 1, 'c', D_Ca, 'a', 0, 'f', 0);
```

```
% % Apply F1 flux for Ca2+ exit(constant flux)
applyBoundaryCondition(model1,"neumann","Face",2,"g",J_F2_high,"q",0);
```

```
% not flux at closed end and sides
applyBoundaryCondition(model1,"neumann","Face",1,"g",0,"q",0);
applyBoundaryCondition(model1,"neumann","Face",3,"g",0,"q",0);
```

```
setInitialConditions(model1,u_total);
```

```
results1 = solvepde(model1,tlist)
```

```
sol1 = results1.NodalSolution;

Sol_time1 = results1.NodalSolution;
timeForSolution1 = toc

tic
% model with constant Ca2+ efflux with calsequestrin
model2 = createpde(1);
model2.Geometry = gm;
meshgeom2 = generateMesh(model2,"Hmax",Mesh_Hmax);

cCoeff = @(location, state) nonlinearDiffusionCoeff(location,state,D_Ca, Kd, u_Casq);
specifyCoefficients(model2, 'm', 0, 'd', 1, 'c', cCoeff, 'a', 0, 'f', 0);

% % Apply F1 flux for Ca2+ exit(constant flux)
applyBoundaryCondition(model2,"neumann","Face",2,"g",J_F2_high,"q",0);

% not flux at closed end and sides
applyBoundaryCondition(model2,"neumann","Face",1,"g",0,"q",0);
applyBoundaryCondition(model2,"neumann","Face",3,"g",0,"q",0);

setInitialConditions(model2,u_total);

results2 = solvepde(model2,tlist)

sol2 = results2.NodalSolution;

Sol_time2 = results2.NodalSolution;
timeForSolution2 = toc
```

```
tic
% model with non-constant Ca2+ efflux without influx without calsequetrin
model3 = createpde(1);
model3.Geometry = gm;
meshgeom3 = generateMesh(model3,"Hmax",Mesh_Hmax);
```

```
specifyCoefficients(model3, 'm', 0, 'd', 1, 'c', D_Ca, 'a', 0, 'f', 0);
```

```
applyBoundaryCondition(model3,"neumann","Face",2,"g",@(location, state) J_t_efflux(location, state,
J_TSR_0_high, u_cyt, u_total, a, V_SR_ratio),"q",0);
```

```
% not flux at closed end and sides
applyBoundaryCondition(model3,"neumann","Face",1,"g",0,"q",0);
applyBoundaryCondition(model3,"neumann","Face",3,"g",0,"q",0);
```

```
setInitialConditions(model3,u_total);
```

```
results3 = solvepde(model3,tlist)
```

```
sol3 = results3.NodalSolution;
```

```
Sol_time3 = results3.NodalSolution;
timeForSolution3 = toc
```

```
% apply the new DCa to the model
tic
% model with non-constant Ca2+ efflux with influx without calsequestrin
model4 = createpde(1);
model4.Geometry = gm;
meshgeom4 = generateMesh(model4,"Hmax",Mesh_Hmax);
```

```
specifyCoefficients(model4, 'm', 0, 'd', 1, 'c', D_Ca, 'a', 0, 'f', 0);
```

```
applyBoundaryCondition(model4,"neumann","Face",2,"g",@(location, state) J_t_efflux(location, state,
J_TSR_0_high, u_cyt, u_total, a, V_SR_ratio),"q",0);

% not flux at closed end and sides
applyBoundaryCondition(model4,"neumann","Face",1,"g",0,"q",0);
applyBoundaryCondition(model4,"neumann","Face",3,"g", J_t_influx,"q",0);

setInitialConditions(model4,u_total);

results4 = solvepde(model4,tlist)

sol4 = results4.NodalSolution;

Sol_time4 = results4.NodalSolution;
timeForSolution4 = toc
%T_half4 = computeHalfLife(solValues4, u_0, T_Spacing);

% model with non-constant Ca2+ efflux without influx with calsequestrin
model5 = createpde(1);
model5.Geometry = gm;
meshgeom5 = generateMesh(model5,"Hmax",Mesh_Hmax);

cCoeff = @(location, state) nonlinearDiffusionCoeff(location,state,D_Ca, Kd, u_Casq);
specifyCoefficients(model5, 'm', 0, 'd', 1, 'c', cCoeff, 'a', 0, 'f', 0);

applyBoundaryCondition(model5,"neumann","Face",2,"g",@(location, state) J_t_efflux_withcasq(location,
state, J_TSR_0_high, u_cyt, u_total, a, V_SR_ratio, u_Casq),"q",0);

% not flux at closed end and sides
applyBoundaryCondition(model5,"neumann","Face",1,"g",0,"q",0);
```

```
applyBoundaryCondition(model5,"neumann","Face",3,"g",0,"q",0);

setInitialConditions(model5,u_total);

results5 = solvepde(model5,tlist)

sol5 = results5.NodalSolution;

Sol_time5 = results5.NodalSolution;
timeForSolution5 = toc

%T_half5 = computeHalfLife(solValues5, u_0, T_Spacing);

% model with non-constant Ca2+ efflux with influx with calsequestrin
model6 = createpde(1);
model6.Geometry = gm;
meshgeom6 = generateMesh(model6,"Hmax",Mesh_Hmax);

cCoeff = @(location, state) nonlinearDiffusionCoeff(location,state,D_Ca, Kd, u_Casq);
specifyCoefficients(model6, 'm', 0, 'd', 1, 'c', cCoeff, 'a', 0, 'f', 0);

applyBoundaryCondition(model6,"neumann","Face",2,"g",@(location, state) J_t_efflux_withcasq(location,
state, J_TSR_0_high, u_cyt, u_total, a, V_SR_ratio, u_Casq),"q",0);

% not flux at closed end and sides
applyBoundaryCondition(model6,"neumann","Face",1,"g",0,"q",0);
applyBoundaryCondition(model6,"neumann","Face",3,"g",J_t_influx,"q",0);

setInitialConditions(model6,u_total);

results6 = solvepde(model6,tlist)
```

```
sol6 = results6.NodalSolution;

Sol_time6 = results6.NodalSolution;
timeForSolution6 = toc

%T_half6 = computeHalfLife(solValues6, u_0, T_Spacing);

% selected_times = [6.25e-4, 0.00125, 0.0025, 0.005, 0.01, 0.02];

% plotting Ca2+ at cylinder for different runs
% plotPDEResults(selected_times, tlist, results1, '3D Solution With D_{Ca} =3\times10^{-10}m^2/s and Constant Ca2+ Efflux', model1)
% plotCalciumBehavior(tlist, results1, '3D Solution at Selected Time Steps With D_{Ca} =3\times10^{-10}m^2/s and Constant Ca2+ Efflux', model1)

% plotting Ca2+ at cylinder for different runs
% plotPDEResults(selected_times, tlist, results2, '3D Solution With D_{Ca} =3\times10^{-10}m^2/s, Constant Ca2+ Efflux and CS present, Without Influx', model2)
% plotCalciumBehavior(tlist, results2, '3D Solution at Selected Time Steps With D_{Ca} =3\times10^{-10}m^2/s, Constant Ca2+ Efflux and CS present, Without Influx', model2)

% plotting Ca2+ at cylinder for different runs
% plotPDEResults(selected_times, tlist, results3, '3D Solution With D_{Ca} =3\times10^{-10}m^2/s and Non-constant Ca2+ Efflux Without Influx and CS', model3)
% plotCalciumBehavior(tlist, results3, '3D Solution at Selected Time Steps With D_{Ca} =3\times10^{-10}m^2/s and Non-constant Ca2+ Efflux Without Influx and CS', model3)

% plotting Ca2+ at cylinder for different runs
% plotPDEResults(selected_times, tlist, results4, '3D Solution With D_{Ca} =3\times10^{-10}m^2/s and Non-constant Ca2+ Efflux, Influx Without CS', model4)
% plotCalciumBehavior(tlist, results4, '3D Solution at Selected Time Steps With D_{Ca} =3\times10^{-10}m^2/s and Non-constant Ca2+ Efflux, Influx Without CS', model4)
```

```
% plotting Ca2+ at cylinder for different runs
% plotPDEResults(selected_times, tlist, results5, '3D Solution With D_{Ca} =3\times10^{-10}m^2/s and Non-constant Ca2+ Efflux and CS Without Influx', model5)
% plotCalciumBehavior(tlist, results5, '3D Solution at Selected Time Steps With D_{Ca} =3\times10^{-10}m^2/s and Non-constant Ca2+ Efflux and CS Without Influx', model5)
```

```
% plotting Ca2+ at cylinder for different runs
% plotPDEResults(selected_times, tlist, results6, '3D Solution With D_{Ca} =3\times10^{-10}m^2/s and Non-constant Ca2+ Efflux, Influx and CS', model6)
% plotCalciumBehavior(tlist, results6, '3D Solution at Selected Time Steps With D_{Ca} =3\times10^{-10}m^2/s and Non-constant Ca2+ Efflux, Influx and CS', model6)
```

```
num_points = 37;
Axis_names = {'At the Middle of SR', 'At the Middle of Cylinder', 'At the Terminal Cisternae'};
Radius_names = {'At the Centre of Cross-section', 'Halfway of Radius', 'At the SR Membrane'};
```

```
%
solAxis1 = extractSolutionAtAxis(results1, T_Resolution, num_points);
solRad1_TC = extractSolutionAtRadius(results1, T_Resolution, num_points, 0);
solRad1_mp = extractSolutionAtRadius(results1, T_Resolution, num_points, l/4);
solRad1_mid = extractSolutionAtRadius(results1, T_Resolution, num_points, l/2); % middle point of SR
efflux1 = compute_efflux(results1, T_Resolution, u_total, J_TSR_0_high, A_TSR, u_cyt)
```

```
% visualizeCt(tlist, num_points, solAxis1, '[Ca^{2+}]-t Plot With D_{Ca} =3\times10^{-10}m^2/s and Constant Ca2+ Efflux')
% plotNLines(solAxis1, tlist, u_0, '[Ca^{2+}]-t Line Plot With D_{Ca} =3\times10^{-10}m^2/s and Constant Ca2+ Efflux',5)
% plotConcentrationProfiles(solAxis1, 5, 'Total SR Element Ca^{2+} Concentration Profile with Constant Ca2+ Efflux')
```

```
solAxis2 = extractSolutionAtAxis(results2, T_Resolution, num_points);
solRad2_TC = extractSolutionAtRadius(results2, T_Resolution, num_points, 0);
solRad2_mp = extractSolutionAtRadius(results2, T_Resolution, num_points, l/4);
```

```
solRad2_mid = extractSolutionAtRadius(results2, T_Resolution, num_points, l/2); % middle point of SR
efflux2 = compute_efflux(results2, T_Resolution, u_total, J_TSR_0_high, A_TSR, u_cyt)
```

```
% visualizeCt(tlist, num_points, solAxis2, '[Ca^{2+}]-t Plot With D_{Ca} =3\times10^{-10}m^2/s, Constant Ca2+ Efflux and CS present, Without Influx')
% plotConcentrationProfiles(solAxis2, 5, 'Total SR Element Ca^{2+} Concentration Profile with Constant Ca2+ Efflux and CS present, Without Influx')
% plotNLines(solAxis2, tlist, u_0, '[Ca^{2+}]-t Line Plot With D_{Ca} =3\times10^{-10}m^2/s, Constant Ca2+ Efflux and CS present, Without Influx',5)
```

```
solAxis3 = extractSolutionAtAxis(results3, T_Resolution, num_points);
solRad3_TC = extractSolutionAtRadius(results3, T_Resolution, num_points, 0);
solRad3_mp = extractSolutionAtRadius(results3, T_Resolution, num_points, l/4);
solRad3_mid = extractSolutionAtRadius(results3, T_Resolution, num_points, l/2); % middle point of SR
efflux3 = compute_efflux(results3, T_Resolution, u_total, J_TSR_0_high, A_TSR, u_cyt)
```

```
% visualizeCt(tlist, num_points, solAxis3, '[Ca^{2+}]-t Plot With D_{Ca} =3\times10^{-10}m^2/s and Non-constant Ca2+ Efflux Without Influx and CS')
% plotConcentrationProfiles(solAxis3, 5, 'Total SR Element Ca^{2+} Concentration Profile with Non-constant Ca2+ Efflux Without Influx and CS')
% plotNLines(solAxis3, tlist, u_0, '[Ca^{2+}]-t Line Plot With D_{Ca} =3\times10^{-10}m^2/s and Non-constant Ca2+ Efflux Without Influx and CS',5)
```

```
solAxis4 = extractSolutionAtAxis(results4, T_Resolution, num_points);
solRad4_TC = extractSolutionAtRadius(results4, T_Resolution, num_points, 0);
solRad4_mp = extractSolutionAtRadius(results4, T_Resolution, num_points, l/4);
solRad4_mid = extractSolutionAtRadius(results4, T_Resolution, num_points, l/2); % middle point of SR
efflux4 = compute_efflux(results4, T_Resolution, u_total, J_TSR_0_high, A_TSR, u_cyt)
```

```
% visualizeCt(tlist, num_points, solAxis4, '[Ca^{2+}]-t Plot With D_{Ca} =3\times10^{-10}m^2/s and Non-constant Ca2+ Efflux, Influx Without CS')
% plotConcentrationProfiles(solAxis4, 5, 'Total SR Element Ca^{2+} Concentration Profile with Non-constant Ca2+ Efflux, Influx Without CS')
```

```
% plotNLines(solAxis4, tlist, u_0, '[Ca^{2+}]-t Line Plot With D_{Ca} =3\times10^{-10}m^2/s and Non-constant Ca2+ Efflux, Influx Without CS',5)
```

```
solAxis5 = extractSolutionAtAxis(results5, T_Resolution, num_points);
solRad5_TC = extractSolutionAtRadius(results5, T_Resolution, num_points, 0);
solRad5_mp = extractSolutionAtRadius(results5, T_Resolution, num_points, l/4);
solRad5_mid = extractSolutionAtRadius(results5, T_Resolution, num_points, l/2); % middle point of SR
efflux5 = compute_efflux(results5, T_Resolution, u_total, J_TSR_0_high, A_TSR, u_cyt)
```

```
% visualizeCt(tlist, num_points, solAxis5, '[Ca^{2+}]-t Plot With D_{Ca} =3\times10^{-10}m^2/s and Non-constant Ca2+ Efflux and CS Without Influx')
% plotConcentrationProfiles(solAxis5, 5, 'Total SR Element Ca^{2+} Concentration Profile with Non-constant Ca2+ Efflux and CS Without Influx')
% plotNLines(solAxis5, tlist, u_0, '[Ca^{2+}]-t Line Plot With D_{Ca} =3\times10^{-10}m^2/s and Non-constant Ca2+ Efflux and CS Without Influx',5)
```

```
solAxis6 = extractSolutionAtAxis(results6, T_Resolution, num_points);
solRad6_TC = extractSolutionAtRadius(results6, T_Resolution, num_points, 0);
solRad6_mp = extractSolutionAtRadius(results6, T_Resolution, num_points, l/4);
solRad6_mid = extractSolutionAtRadius(results6, T_Resolution, num_points, l/2); % middle point of SR
efflux6 = compute_efflux(results6, T_Resolution, u_total, J_TSR_0_high, A_TSR, u_cyt)
```

```
% visualizeCt(tlist, num_points, solAxis6, '[Ca^{2+}]-t Plot With D_{Ca} =3\times10^{-10}m^2/s and Non-constant Ca2+ Efflux, Influx and CS')
% plotConcentrationProfiles(solAxis6, 5, 'Total SR Element Ca^{2+} Concentration Profile with Non-constant Ca2+ Efflux, Influx and CS')
% plotNLines(solAxis6, tlist, u_0, '[Ca^{2+}]-t Line Plot With D_{Ca} =3\times10^{-10}m^2/s and Non-constant Ca2+ Efflux, Influx and CS',5)
```

```
% plotting 6 plots in one
solValuesArray_axis = {solAxis1,solAxis2,solAxis3,solAxis5,solAxis4,solAxis6};
plot_titles = {'Constant release efflux without Casq', 'Constant release efflux with Casq','Non-constant release efflux without pump influx without Casq', 'Non-constant release efflux without pump influx with Casq', 'Non-
```

```
constant release efflux with pump influx without Casq', 'Non-constant release efflux with pump influx and Casq'};
visualizeMultipleCt_axis(tlist, num_points, solValuesArray_axis, plot_titles, 'Total [Ca^{2+}] Diffusion Behaviour Along the Axis of SR Element Over Time')

% plotMultipleThreeLines(solValuesArray_axis, tlist, u_0, plot_titles, Axis_names, 'Line Plot of [Ca^{2+}] Diffusion Behaviour Along the Axis of SR Element Over Time')


conc_diff = zeros(length(solValuesArray_axis),1 + T_Resolution);
for i = 1:length(solValuesArray_axis)
        % Get the corresponding solValues
        solValues = solValuesArray_axis{i};

        % Determine indices for the required positions
        idx_bottom = 1;                % Position at (0,0,0)
        idx_top = size(solValues, 1);  % Position at (0,0,height)

        % Calculate concentration difference for each time point
        conc_diff(i,:) = solValues(idx_bottom, :) - solValues(idx_top, :);
end

conc_diff_end = conc_diff(:,end)
writematrix(conc_diff_end, 'conc_diff_end_20ms.csv');



first_reach_time = zeros(6, 1);

for row = 1:6
final_value = conc_diff(row, end); % Get the target value for this row
for col = 1:1001
        if isequal(round(conc_diff(row, col), 4, 'significant'), round(final_value, 4, 'significant'))
                first_reach_time(row) = tlist(col) ; % Save the first index where it matches
                break;
```

```
    end
end
end
writematrix(first_reach_time, 'first_reach_time_20ms.csv');


solValuesArray_TC = {solRad1_TC,solRad2_TC,solRad3_TC,solRad5_TC,solRad4_TC,solRad6_TC};
visualizeMultipleCt_radius(tlist, num_points, solValuesArray_TC, plot_titles, 'Total [Ca^{2+}] Diffusion Behaviour Along the Radius of SR Element at Terminal Cisternae Over Time')

solRad_diff_TC = zeros(6,1);
for i = 1:6
diff = solValuesArray_TC{i}(1,end)-solValuesArray_TC{i}(end,end);
solRad_diff_TC(i) = diff;
end
solRad_diff_TC
writematrix(solRad_diff_TC, 'solRad_diff_TC_20ms.csv');

% plotMultipleThreeLines(solValuesArray_TC, tlist, u_0, plot_titles, Radius_names, 'Line Plot of [Ca^{2+}] Diffusion Behaviour Along the Radius of SR Element at Terminal Cisternae Over Time')


solValuesArray_mp = {solRad1_mp,solRad2_mp,solRad3_mp,solRad5_mp,solRad4_mp,solRad6_mp};
visualizeMultipleCt_radius(tlist, num_points, solValuesArray_mp, plot_titles, 'Total [Ca^{2+}] Diffusion Behaviour Along the SR Radius at Middle of Half SR Element Over Time')
% plotMultipleThreeLines(solValuesArray_mp, tlist, u_0, plot_titles, Radius_names, 'Line Plot of [Ca^{2+}] Diffusion Behaviour Along the Radius at Middle of Half SR Over Time')


solValuesArray_mid = {solRad1_mid,solRad2_mid,solRad3_mid,solRad5_mid,solRad4_mid,solRad6_mid};
visualizeMultipleCt_radius(tlist, num_points, solValuesArray_mid, plot_titles, 'Total [Ca^{2+}] Diffusion Behaviour Along the Radius at the Center of SR Element Over Time')



solRad_diff_mid = zeros(6,1);
```

```
for i = 1:6
diff = solValuesArray_mid{i}(1,end)-solValuesArray_mid{i}(end,end);
solRad_diff_mid(i) = diff;
end
solRad_diff_mid
writematrix(solRad_diff_mid, 'solRad_diff_mid_20ms.csv');
```

```
plotMultipleNLines(solValuesArray_axis, tlist, u_total, plot_titles, 5, '[Ca^{2+}]_{total}-t Line Plot of Calcium Diffusion Behaviour Along the SR Axis Over Time')
```

```
plotMultipleConcentrationProfiles(solValuesArray_axis, u_total, plot_titles, 5,'Total SR Element Ca^{2+} Concentration Profiles Along the SR Axis Over Time')
```

Plotting

```
figure;
```

```
y1=solAxis1(end,:);
y1(y1 < 0) = NaN;
l1 = plot(tlist,y1,'LineWidth', 2);
hold on;
```

```
y2=solAxis2(end,:);
y2(y2 < 0) = NaN;
l2 = plot(tlist,y2,'LineWidth', 2);
hold on;
```

```
l3 = plot(tlist,solAxis3(end,:),'LineWidth', 2);
hold on
l4 = plot(tlist,solAxis4(end,:),'LineWidth', 2);
hold on
l5 = plot(tlist,solAxis5(end,:),'LineWidth', 2);
hold on
```

```
l6 = plot(tlist,solAxis6(end,:),'LineWidth', 2);
hold on

% plot lines indicating time to empty SR
y = [0, u_0]; % Y-range for the line

%plot([T_half2(1), T_half2(1)], y, '--g');
%text(T_half2(1)-0.02, 0, ['t_{half without influx} = ', num2str(T_half2(1)), 's']);

%plot([T_half3(1), T_half3(1)], y, '--r');
%text(T_half3(1)-0.02, u_0, ['t_{half with influx} = ', num2str(T_half3(1)), 's']);

grid on; % Adds a grid to the plot
xlabel('Time(s)', 'FontSize', 14, 'FontName', 'Arial'); % Label for the X-axis
ylabel('Concentration(mol/(m^3)', 'FontSize', 14, 'FontName', 'Arial'); % Label for the Y-axis
t = 'Plot of Total [Ca^{2+}] Over Time at the Centre of Release Site';
title(t, 'FontSize', 16, 'FontName', 'Arial');
legend([l1, l2, l3, l4, l5, l6], {'Constant efflux without Casq','Constant efflux with Casq', 'Non-constant efflux without influx without Casq', 'Non-constant efflux with influx without Casq','Non-constant efflux without influx with Casq', 'Non-constant efflux with influx with Casq'});

% Thicker axis and ticks
ax = findall(gcf, 'Type', 'axes'); % Get all axes in the current figure
set(ax, 'LineWidth', 2); % Apply thick axis lines to all subplots
set(gca, 'FontSize', 14, 'FontName', 'Arial');

hold off

safe_name = regexprep(t, '[^\w]', '_'); % Replace non-word characters with underscores
png_filename = sprintf('%s.png', safe_name);
pdf_filename = sprintf('%s.pdf', safe_name);
fig_filename = sprintf('%s.fig', safe_name);
```

```
exportgraphics(gcf, png_filename, 'Resolution', 300); % Save as PNG with high resolution
exportgraphics(gcf, pdf_filename, 'ContentType', 'vector'); % Save as a vectorized PDF
savefig(fig_filename);
```

```
plotConcDifferenceSamePlot(solValuesArray_axis, tlist, plot_titles, '[Ca^{2+}]_{total} Difference Between Centre and Release Site of SR Element Over Time')
```

Tranforming the [Ca2+] total in calsequestrin cases to [Ca2+] free

```
function Ca_free = convertConcentration(solValues, Kd, u_Casq)

% Ensure solValues is non-negative to avoid complex results
solValues(solValues < 0) = 0;

% Compute free [Ca2+] using the given equation
Ca_free = (-Kd + solValues - u_Casq + sqrt((Kd - solValues + u_Casq).^2 + 4 .* solValues .* Kd)) / 2;
end
```

```
solAxis1f = extractSolutionAtAxis(results1f, T_Resolution, num_points);
solRad1f_TC = extractSolutionAtRadius(results1f, T_Resolution, num_points, 0);
solRad1f_mp = extractSolutionAtRadius(results1f, T_Resolution, num_points, l/4);
solRad1f_mid = extractSolutionAtRadius(results1f, T_Resolution, num_points, l/2); % middle point of SR
efflux1f = compute_efflux(results1f, T_Resolution, u_0, J_TSR_0_high, A_TSR, u_cyt)

% visualizeCt(tlist, num_points, solAxis1f, '[Ca^{2+}]_{free}-t Plot With D_{Ca} =3\times10^{-10}m^2/s and Constant Ca2+ Efflux')
% plotNLines(solAxis1f, tlist, u_0, '[Ca^{2+}]_{free}-t Line Plot With D_{Ca} =3\times10^{-10}m^2/s and Constant Ca2+ Efflux',5)
% plotConcentrationProfiles(solAxis1f, 5, 'SR Element Free Ca^{2+} Concentration Profile with Constant Ca2+ Efflux')
```

```
solAxis2f = convertConcentration(solAxis2, Kd, u_Casq);
solRad2f_TC = convertConcentration(solRad2_TC, Kd, u_Casq);
solRad2f_mp = convertConcentration(solRad2_mp, Kd, u_Casq);
```

```
solRad2f_mid = convertConcentration(solRad2_mid, Kd, u_Casq); % middle point of SR
```

```
% visualizeCt(tlist, num_points, solAxis2f, '[Ca^{2+}]_{free}-t Plot With D_{Ca} =3\times10^{-10}m^2/s, Constant Ca2+ Efflux and CS present, Without Influx')
% plotConcentrationProfiles(solAxis2f, 5, 'SR Element Free Ca^{2+} Concentration Profile with Constant Ca2+ Efflux and CS present, Without Influx')
% plotNLines(solAxis2f, tlist, u_0, '[Ca^{2+}]_{free}-t Line Plot With D_{Ca} =3\times10^{-10}m^2/s, Constant Ca2+ Efflux and CS present, Without Influx',5)
```

```
solAxis3f = extractSolutionAtAxis(results3f, T_Resolution, num_points);
solRad3f_TC = extractSolutionAtRadius(results3f, T_Resolution, num_points, 0);
solRad3f_mp = extractSolutionAtRadius(results3f, T_Resolution, num_points, l/4);
solRad3f_mid = extractSolutionAtRadius(results3f, T_Resolution, num_points, l/2); % middle point of SR
efflux3f = compute_efflux(results3f, T_Resolution, u_0, J_TSR_0_high, A_TSR, u_cyt)
```

```
% visualizeCt(tlist, num_points, solAxis3f, '[Ca^{2+}]_{free}-t Plot With D_{Ca} =3\times10^{-10}m^2/s and Non-constant Ca2+ Efflux Without Influx and CS')
% plotConcentrationProfiles(solAxis3f, 5, 'SR Element Free Ca^{2+} Concentration Profile with Non-constant Ca2+ Efflux Without Influx and CS')
% plotNLines(solAxis3f, tlist, u_0, '[Ca^{2+}]_{free}-t Line Plot With D_{Ca} =3\times10^{-10}m^2/s and Non-constant Ca2+ Efflux Without Influx and CS',5)
```

```
solAxis4f = extractSolutionAtAxis(results4f, T_Resolution, num_points);
solRad4f_TC = extractSolutionAtRadius(results4f, T_Resolution, num_points, 0);
solRad4f_mp = extractSolutionAtRadius(results4f, T_Resolution, num_points, l/4);
solRad4f_mid = extractSolutionAtRadius(results4f, T_Resolution, num_points, l/2); % middle point of SR
efflux4f = compute_efflux(results4f, T_Resolution, u_0, J_TSR_0_high, A_TSR, u_cyt)
```

```
% visualizeCt(tlist, num_points, solAxis4f, '[Ca^{2+}]_{free}-t Plot With D_{Ca} =3\times10^{-10}m^2/s and Non-constant Ca2+ Efflux, Influx Without CS')
% plotConcentrationProfiles(solAxis4f, 5, 'SR Element Free Ca^{2+} Concentration Profile with Non-constant Ca2+ Efflux, Influx Without CS')
% plotNLines(solAxis4f, tlist, u_0, '[Ca^{2+}]_{free}-t Line Plot With D_{Ca} =3\times10^{-10}m^2/s and Non-constant Ca2+ Efflux, Influx Without CS',5)
```

```
solAxis5f = convertConcentration(solAxis5, Kd, u_Casq);
solRad5f_TC = convertConcentration(solRad5_TC, Kd, u_Casq);
solRad5f_mp = convertConcentration(solRad5_mp, Kd, u_Casq);
solRad5f_mid = convertConcentration(solRad5_mid, Kd, u_Casq); % middle point of SR
% visualizeCt(tlist, num_points, solAxis5f, '[Ca^{2+}]_{free}-t Plot With D_{Ca} =3\times10^{-10}m^2/s and Non-constant Ca2+ Efflux and CS Without Influx')
% plotConcentrationProfiles(solAxis5f, 5, 'SR Element Free Ca^{2+} Concentration Profile with Non-constant Ca2+ Efflux and CS Without Influx')
% plotNLines(solAxis5f, tlist, u_0, '[Ca^{2+}]_{free}-t Line Plot With D_{Ca} =3\times10^{-10}m^2/s and Non-constant Ca2+ Efflux and CS Without Influx',5)
```

```
solAxis6f = convertConcentration(solAxis6, Kd, u_Casq);
solRad6f_TC = convertConcentration(solRad6_TC, Kd, u_Casq);
solRad6f_mp = convertConcentration(solRad6_mp, Kd, u_Casq);
solRad6f_mid = convertConcentration(solRad6_mid, Kd, u_Casq); % middle point of SR
% visualizeCt(tlist, num_points, solAxis6f, '[Ca^{2+}]_{free}-t Plot With D_{Ca} =3\times10^{-10}m^2/s and Non-constant Ca2+ Efflux, Influx and CS')
% plotConcentrationProfiles(solAxis6f, 5, 'SR Element Free Ca^{2+} Concentration Profile with Non-constant Ca2+ Efflux, Influx and CS')
% plotNLines(solAxis6f, tlist, u_0, '[Ca^{2+}]_{free}-t Line Plot With D_{Ca} =3\times10^{-10}m^2/s and Non-constant Ca2+ Efflux, Influx and CS',5)
```

```
% plotting 6 plots in one
solValuesArrayf_axis = {solAxis1f,solAxis2f,solAxis3f,solAxis5f,solAxis4f,solAxis6f};
plot_titles = {'Constant release efflux without Casq', 'Constant release efflux with Casq','Non-constant release efflux without pump influx without Casq', 'Non-constant release efflux without pump influx with Casq', 'Non-constant release efflux with pump influx without Casq', 'Non-constant release efflux with pump influx and Casq'};
visualizeMultipleCt_axis(tlist, num_points, solValuesArrayf_axis, plot_titles, '[Ca^{2+}]_{free} Diffusion Behaviour Along the Axis of SR Element Over Time')
% plotMultipleThreeLines(solValuesArray_axis, tlist, u_0, plot_titles, Axis_names, 'Line Plot of [Ca^{2+}] Diffusion Behaviour Along the Axis of SR Element Over Time')
```

```
conc_diff_f = zeros(length(solValuesArrayf_axis),1 + T_Resolution);
```

```
for i = 1:length(solValuesArrayf_axis)
    % Get the corresponding solValues
    solValues = solValuesArrayf_axis{i};

    % Determine indices for the required positions
    idx_bottom = 1;                % Position at (0,0,0)
    idx_top = size(solValues, 1);  % Position at (0,0,height)

    % Calculate concentration difference for each time point
    conc_diff_f(i,:) = solValues(idx_bottom, :) - solValues(idx_top, :);
end

conc_diff_end_f = conc_diff_f(:,end)

writematrix(conc_diff_end_f, 'conc_diff_end_f_20ms.csv');

first_reach_time_f = zeros(6, 1);

for row = 1:6
final_value = conc_diff_f(row, end); % Get the target value for this row
for col = 1:1001
    if isequal(round(conc_diff_f(row, col), 4, 'significant'), round(final_value, 4, 'significant'))
            first_reach_time_f(row) = tlist(col) ; % Save the first index where it matches
            break;
    end
end
end
writematrix(first_reach_time_f, 'first_reach_time_f_20ms.csv');


solValuesArrayf_TC = {solRad1f_TC,solRad2f_TC,solRad3f_TC,solRad5f_TC,solRad4f_TC,solRad6f_TC};
visualizeMultipleCt_radius(tlist, num_points, solValuesArrayf_TC, plot_titles, '[Ca^{2+}]_{free} Diffusion Behaviour Along the Radius of SR Element at Terminal Cisternae Over Time')
```

```
% plotMultipleThreeLines(solValuesArray_TC, tlist, u_0, plot_titles, Radius_names, 'Line Plot of [Ca^{2+}] Diffusion Behaviour Along the Radius of SR Element at Terminal Cisternae Over Time')
```

```
solRad_diff_TCf = zeros(6,1);
for i = 1:6
diff = solValuesArrayf_TC{i}(1,end)-solValuesArrayf_TC{i}(end,end);
solRad_diff_TCf(i) = diff;
end
solRad_diff_TCf
writematrix(solRad_diff_TCf, 'solRad_diff_TCf_20ms.csv');
```

```
solValuesArrayf_mp = {solRad1f_mp,solRad2f_mp,solRad3f_mp,solRad5f_mp,solRad4f_mp,solRad6f_mp};
visualizeMultipleCt_radius(tlist, num_points, solValuesArrayf_mp, plot_titles, '[Ca^{2+}]_{free} Diffusion Behaviour Along the SR Radius at Middle of Half SR Element Over Time')
% plotMultipleThreeLines(solValuesArray_mp, tlist, u_0, plot_titles, Radius_names, 'Line Plot of [Ca^{2+}] Diffusion Behaviour Along the Radius at Middle of Half SR Over Time')
```

```
solValuesArrayf_mid = {solRad1f_mid,solRad2f_mid,solRad3f_mid,solRad5f_mid,solRad4f_mid,solRad6f_mid};
visualizeMultipleCt_radius(tlist, num_points, solValuesArrayf_mid, plot_titles, '[Ca^{2+}]_{free} Diffusion Behaviour Along the Radius at the Center of SR Element Over Time')
```

```
solRad_diff_midf = zeros(6,1);
for i = 1:6
diff = solValuesArrayf_mid{i}(1,end)-solValuesArrayf_mid{i}(end,end);
solRad_diff_midf(i) = diff;
end
solRad_diff_midf
writematrix(solRad_diff_midf, 'solRad_diff_midf_20ms.csv');
```

```
plotMultipleNLines(solValuesArrayf_axis, tlist, u_0, plot_titles, 5, '[Ca^{2+}]_{free}-t Line Plot of Calcium Diffusion Behaviour Along the SR Axis Over Time')
```

```
plotMultipleConcentrationProfiles(solValuesArrayf_axis, u_0, plot_titles, 5, 'Free SR Element Ca^{2+}
Concentration Profiles Along the SR Axis Over Time')
```

```
figure;
```

```
y1=solAxis1f(end,:);
y1(y1 < 0) = NaN;
l1 = plot(tlist,y1,'LineWidth', 2);
hold on;
```

```
y2=solAxis2f(end,:);
y2(y2 < 0) = NaN;
l2 = plot(tlist,y2,'LineWidth', 2);
hold on;
```

```
l3 = plot(tlist,solAxis3f(end,:),'LineWidth', 2);
hold on
l4 = plot(tlist,solAxis4f(end,:),'LineWidth', 2);
hold on
l5 = plot(tlist,solAxis5f(end,:),'LineWidth', 2);
hold on
l6 = plot(tlist,solAxis6f(end,:),'LineWidth', 2);
hold on
```

```
% plot lines indicating time to empty SR
y = [0, u_0]; % Y-range for the line
```

```
%plot([T_half2(1), T_half2(1)], y, '--g');
%text(T_half2(1)-0.02, 0, ['t_{half without influx} = ', num2str(T_half2(1)), 's']);
```

```
%plot([T_half3(1), T_half3(1)], y, '--r');
%text(T_half3(1)-0.02, u_0, ['t_{half with influx} = ', num2str(T_half3(1)), 's']);
```

```
grid on; % Adds a grid to the plot
xlabel('Time(s)', 'FontSize', 14, 'FontName', 'Arial'); % Label for the X-axis
ylabel('Concentration(mol/(m^3)', 'FontSize', 14, 'FontName', 'Arial'); % Label for the Y-axis
t = 'Plot of Free [Ca^{2+}] Over Time at the Centre of Release Site';
title(t, 'FontSize', 16, 'FontName', 'Arial');
legend([l1, l2, l3, l4, l5, l6], {'Constant efflux without Casq','Constant efflux with Casq', 'Non-constant efflux without influx without Casq', 'Non-constant efflux with influx without Casq','Non-constant efflux without influx with Casq', 'Non-constant efflux with influx with Casq'});

% Thicker axis and ticks
ax = findall(gcf, 'Type', 'axes'); % Get all axes in the current figure
set(ax, 'LineWidth', 2); % Apply thick axis lines to all subplots
set(gca, 'FontSize', 14, 'FontName', 'Arial');

hold off

safe_name = regexprep(t, '[^\w]', '_'); % Replace non-word characters with underscores
png_filename = sprintf('%s.png', safe_name);
pdf_filename = sprintf('%s.pdf', safe_name);
fig_filename = sprintf('%s.fig', safe_name);

exportgraphics(gcf, png_filename, 'Resolution', 300); % Save as PNG with high resolution
exportgraphics(gcf, pdf_filename, 'ContentType', 'vector'); % Save as a vectorized PDF
savefig(fig_filename);

plotConcDifferenceSamePlot(solValuesArrayf_axis, tlist, plot_titles, '[Ca^{2+}]_{free} Difference Between Centre and Release Site of SR Element Over Time')

figure;

hold on;
plot(tlist, efflux3, 'DisplayName', plot_titles{3},'LineWidth', 1.5);
plot(tlist, efflux5, 'DisplayName', plot_titles{4},'LineWidth', 1.5);
```

```
plot(tlist, efflux4, 'DisplayName', plot_titles{5},'LineWidth', 1.5);
plot(tlist, efflux6, 'DisplayName', plot_titles{6},'LineWidth', 1.5);

legend('show');

t = 'Plot of Total Rate of Total Ca^{2+} Efflux Over Time at Terminal Cisternae';
title(t, 'FontSize', 16, 'FontName', 'Arial');
xlabel('Time(s)', 'FontSize', 14, 'FontName', 'Arial');
ylabel('Rate of Efflux(mol/s)', 'FontSize', 14, 'FontName', 'Arial');

% Thicker axis and ticks
ax = findall(gcf, 'Type', 'axes'); % Get all axes in the current figure
set(ax, 'LineWidth', 2); % Apply thick axis lines to all subplots
set(gca, 'FontSize', 14, 'FontName', 'Arial');

grid on;

hold off;
safe_name = regexprep(t, '[^\w]', '_'); % Replace non-word characters with underscores
png_filename = sprintf('%s.png', safe_name);
pdf_filename = sprintf('%s.pdf', safe_name);
fig_filename = sprintf('%s.fig', safe_name);

exportgraphics(gcf, png_filename, 'Resolution', 300); % Save as PNG with high resolution
exportgraphics(gcf, pdf_filename, 'ContentType', 'vector'); % Save as a vectorized PDF
savefig(fig_filename);

figure;

hold on;
plot(tlist, efflux3f, 'DisplayName', plot_titles{3},'LineWidth', 1.5);
```

```
plot(tlist, efflux5, 'DisplayName', plot_titles{4},'LineWidth', 1.5);
plot(tlist, efflux4f, 'DisplayName', plot_titles{5},'LineWidth', 1.5);
plot(tlist, efflux6, 'DisplayName', plot_titles{6},'LineWidth', 1.5);

legend('show');

t = 'Plot of Total Rate of Free Ca^{2+} Efflux Over Time at Terminal Cisternae';
title(t, 'FontSize', 16, 'FontName', 'Arial');
xlabel('Time(s)', 'FontSize', 14, 'FontName', 'Arial');
ylabel('Rate of Efflux(mol/s)', 'FontSize', 14, 'FontName', 'Arial');

% Thicker axis and ticks
ax = findall(gcf, 'Type', 'axes'); % Get all axes in the current figure
set(ax, 'LineWidth', 2); % Apply thick axis lines to all subplots
set(gca, 'FontSize', 14, 'FontName', 'Arial');

grid on;

hold off;
safe_name = regexprep(t, '[^\w]', '_'); % Replace non-word characters with underscores
png_filename = sprintf('%s.png', safe_name);
pdf_filename = sprintf('%s.pdf', safe_name);
fig_filename = sprintf('%s.fig', safe_name);

exportgraphics(gcf, png_filename, 'Resolution', 300); % Save as PNG with high resolution
exportgraphics(gcf, pdf_filename, 'ContentType', 'vector'); % Save as a vectorized PDF
savefig(fig_filename);

zPoints = linspace(0, 1800e-9, num_points);

figure; % Create a new figure
```

```
imagesc(tlist, zPoints, solAxis6); % Use imagesc for a 2D heatmap

% Customize the color map and color bar
colormap(jet);
cb = colorbar();
ylabel(cb,'Concentration (mol/m^3)','FontSize',14,'Rotation',90)

% Add labels and title
xlabel('Time (s)', 'FontSize', 14, 'FontName', 'Arial');
ylabel('Location (m)', 'FontSize', 14, 'FontName', 'Arial');

% Improve visualization
set(gca, 'YDir', 'normal'); % Ensure the Y-axis direction is correct

% Thicker axis and ticks
ax = findall(gcf, 'Type', 'axes'); % Get all axes in the current figure
set(ax, 'LineWidth', 2); % Apply thick axis lines to all subplots
set(gca, 'FontSize', 14, 'FontName', 'Arial');

% Add five evenly spaced vertical lines using parula colormap
num_lines = 5;
x_positions = linspace(0, max(tlist), num_lines); % Create positions

colors = parula(num_lines); % Get five colors from parula

hold on;
for i = 1:num_lines
xline(x_positions(i), 'Color', [1, 1, 1], 'LineWidth', 2);

end
```

```
% Add custom colored x-tick labels
y_max = max(zPoints); % Place labels slightly below the axis
for i = 1:num_lines
text(x_positions(i), y_max + 0.05 * (max(zPoints) - min(zPoints)), ...
        sprintf('%.4f s', x_positions(i)), ...
        'Color', colors(i, :), 'FontSize', 14, 'FontWeight', 'bold', ...
        'HorizontalAlignment', 'center');
end
hold off;
```

```
% Save figures
savefig('Vertical_Slice_CM.fig');
saveas(gcf, 'Vertical_Slice_CM.pdf');
saveas(gcf, 'Vertical_Slice_CM.png');
```

```
zPoints = linspace(0, 1800e-9, num_points);
figure; % Create a new figure
imagesc(tlist, zPoints, solAxis6); % Use imagesc for a 2D heatmap

% Customize the color map and color bar
colormap(jet);
cb = colorbar();
ylabel(cb,'Concentration (mol/m^3)','FontSize',14,'Rotation',90)
```

```
% Add labels and title
xlabel('Time (s)', 'FontSize', 14, 'FontName', 'Arial');
ylabel('Location (m)', 'FontSize', 14, 'FontName', 'Arial');
```

```
% Improve visualization
set(gca, 'YDir', 'normal'); % Ensure the Y-axis direction is correct
```

```
% Thicker axis and ticks
ax = findall(gcf, 'Type', 'axes'); % Get all axes in the current figure
set(ax, 'LineWidth', 2); % Apply thick axis lines to all subplots
```

```
set(gca, 'FontSize', 14, 'FontName', 'Arial');

% Add five evenly spaced vertical lines using parula colormap
num_lines = 5;
y_positions = linspace(0, max(l/2), num_lines); % Create positions

colors = cool(num_lines); % Get five colors from parula

hold on;
for i = 1:num_lines
yline(y_positions(i), 'Color', [1, 1, 1], 'LineWidth', 2);

end

% Add custom colored y-tick labels
x_max = max(tlist);
for i = 1:num_lines
text(3*x_max/5, y_positions(i), ...
        sprintf('%.0f nm', 1e9*y_positions(i)), ...
        'Color', colors(i, :), 'FontSize', 14, 'FontWeight', 'bold', ...
        'HorizontalAlignment', 'center');

text(2*x_max/5, y_positions(i), ...
        sprintf('Position %.0f :', num_lines-i+1), ...
        'Color', colors(i, :), 'FontSize', 14, 'FontWeight', 'bold', ...
        'HorizontalAlignment', 'center');
end
hold off;

% Save figures
savefig('Horizontal_Slice_CM.fig');
saveas(gcf, 'Horizontal_Slice_CM.pdf');
```

```
saveas(gcf, 'Horizontal_Slice_CM.png');
```

Exporting concentration difference

```
% Define the list of solValues matrices
% solValuesArray_axis
% solValuesArrayf_axis
% Row labels: plot_titles

% Initialize storage for concentration differences
num_experiments = length(solValuesArray_axis);
conc_diff_data = [];

% Compute concentration differences
for i = 1:num_experiments
conc_diff = calculateConcDifference(solValuesArray_axis{i});
conc_diff_data = [conc_diff_data; conc_diff];
end

% Create a cell array for writing into CSV
csv_data = ["Experiment/Time(s)", arrayfun(@num2str, tlist, 'UniformOutput', false)];
csv_data = [csv_data; [plot_titles', num2cell(conc_diff_data)]];

% Define output filename
filename = 'Total_concentration_differences.csv';

% Write cell array to CSV file
writematrix(csv_data, filename);

disp(['CSV file saved as ', filename]);

% Initialize storage for concentration differences
num_experiments = length(solValuesArrayf_axis);
```

```
conc_diff_data = [];
```

```
% Compute concentration differences
for i = 1:num_experiments
conc_diff = calculateConcDifference(solValuesArrayf_axis{i});
conc_diff_data = [conc_diff_data; conc_diff];
end
```

```
% Create a cell array for writing into CSV
csv_data = ["Experiment/Time(s)", arrayfun(@num2str, tlist, 'UniformOutput', false)];
csv_data = [csv_data; [plot_titles', num2cell(conc_diff_data)]];
```

```
% Define output filename
filename = 'Free_concentration_differences.csv';
```

```
% Write cell array to CSV file
writematrix(csv_data, filename);
```

```
disp(['CSV file saved as ', filename]);
```

Exporting efflux rate

```
% results
results_list = {results3, results5, results4, results6};
titles = {'Non-constant release efflux without pump influx without Casq', 'Non-constant release efflux without pump influx with Casq', 'Non-constant release efflux with pump influx without Casq', 'Non-constant release efflux with pump influx and Casq'};
```

```
% Initialize storage for concentration differences
num_experiments = length(results_list);
conc_diff_data = [];
```

```
efflux_data = [];
```

```
for i = 1:num_experiments
efflux = compute_efflux(results3f, T_Resolution, u_0, J_TSR_0_high, A_TSR, u_cyt);
efflux_data = [efflux_data; efflux];
end

% Create a cell array for writing efflux into CSV
csv_data_efflux = ["Experiment/Efflux Rate(mol/s)", arrayfun(@num2str, tlist, 'UniformOutput', false)];
csv_data_efflux = [csv_data_efflux; [titles', num2cell(efflux_data)]];

% Define output filename for efflux
filename_efflux = 'Total_efflux_data.csv';

% Write efflux data to CSV file
writematrix(csv_data_efflux, filename_efflux);

disp(['CSV file saved as ', filename_efflux]);

% Parameters for the cylinder
theta = linspace(0, 2 * pi, 100);  % Half cylinder (0 to pi)
z = linspace(0, 1e9 * l/2, 100);
[Theta, Z] = meshgrid(theta, z);
X = r_dSR * cos(Theta) * 1e9;
Y = r_dSR * sin(Theta) * 1e9;
z_split = 1e9 * l * 0.44;

% Plot the cylinder
figure;
surf(X, Y, Z, 'FaceColor', [0.7, 0.7, 0.7], 'EdgeColor', 'none', 'FaceAlpha', 0.5);
hold on;

% Plot the circular base and top
fill3(r_dSR * cos(theta) * 1e9, r_dSR * sin(theta) * 1e9, zeros(size(theta)), [0.3, 0.3, 0.3], 'FaceAlpha', 0.5);
```

```
fill3(r_dSR * cos(theta) * 1e9, r_dSR * sin(theta) * 1e9, 1e9 * l/2 * ones(size(theta)), [0.7, 0.7, 0.7],
'FaceAlpha', 0.5);
fill3(r_dSR * cos(theta) * 1e9, r_dSR * sin(theta) * 1e9, z_split * ones(size(theta)), [0, 0, 0], 'FaceAlpha', 0.5);

text(15, -15, 1700, sprintf('Terminal Cisternae'), ...
        'FontSize', 12, 'FontWeight', 'bold', 'FontName', 'Arial', 'Color', 'k', ...
        'HorizontalAlignment', 'left', 'VerticalAlignment', 'bottom');

text(15, -15, 800, sprintf('Longitudinal SR'), ...
        'FontSize', 12, 'FontWeight', 'bold', 'FontName', 'Arial', 'Color', 'k', ...
        'HorizontalAlignment', 'left', 'VerticalAlignment', 'bottom');

% Adjust the aspect ratio
ax = gca; % Get the current axes handle
ax.DataAspectRatio = [1, 1, 12]; % Make Z axis units twice as long as X and Y

% Enhance the view
grid on;
title('Improved geometry', 'FontSize', 16, 'FontName', 'Arial');
xlabel('X (nm)', 'FontSize', 14, 'FontName', 'Arial');
ylabel('Y (nm)', 'FontSize', 14, 'FontName', 'Arial');
zlabel('Z (nm)', 'FontSize', 14, 'FontName', 'Arial');
zlim([0, l * 0.5 * 1e9])
view([-30, 30]);  % View angle for better visualization

% Thicker axis and ticks
ax = findall(gcf, 'Type', 'axes'); % Get all axes in the current figure
set(ax, 'LineWidth', 2); % Apply thick axis lines to all subplots
set(gca, 'FontSize', 14, 'FontName', 'Arial');

xticks([-15, 0, 15])
yticks([-15, 0, 15])
```

```
hold off
savefig('NewGeometry.fig');
saveas(gcf,'NewGeometry.pdf');
saveas(gcf,'NewGeometry.png');
```

Back checking cases with casq from free to total (2 5 6)

Tranforming the [Ca2+] free in calsequestrin cases to [Ca2+] total

```
function Ca_total = invertConcentration(solValues, Kd, u_Casq)

% Ensure solValues is non-negative to avoid complex results
solValues(solValues < 0) = 0;

% Compute free [Ca2+] using the given equation
Ca_total = (Kd + solValues + u_Casq) .* solValues ./ (solValues + Kd);
end
```

```
solAxis2t = invertConcentration(solAxis2f, Kd, u_Casq);
solRad2t_TC = invertConcentration(solRad2f_TC, Kd, u_Casq);
solRad2t_mp = invertConcentration(solRad2f_mp, Kd, u_Casq);
solRad2t_mid = invertConcentration(solRad2f_mid, Kd, u_Casq);

solAxis5t = invertConcentration(solAxis5f, Kd, u_Casq);
solRad5t_TC = invertConcentration(solRad5f_TC, Kd, u_Casq);
solRad5t_mp = invertConcentration(solRad5f_mp, Kd, u_Casq);
solRad5t_mid = invertConcentration(solRad5f_mid, Kd, u_Casq);

solAxis6t = invertConcentration(solAxis6f, Kd, u_Casq);
solRad6t_TC = invertConcentration(solRad6f_TC, Kd, u_Casq);
solRad6t_mp = invertConcentration(solRad6f_mp, Kd, u_Casq);
solRad6t_mid = invertConcentration(solRad6f_mid, Kd, u_Casq);
```

```
solValuesArray_Axischeck = {solAxis2,solAxis2t,solAxis5,solAxis5t,solAxis6,solAxis6t};
check_titles = {'Constant release efflux with Casq','Constant release efflux with Casq(inversed)','Non-constant release efflux without pump influx with Casq', 'Non-constant release efflux without pump influx with Casq(inversed)','Non-constant release efflux with pump influx and Casq','Non-constant release efflux without pump influx with Casq(inversed)','Non-constant release efflux with pump influx and Casq(inversed)'};
visualizeMultipleCt_radius(tlist, num_points,solValuesArray_Axischeck, check_titles, 'Check axis')
```
